# High Gas-Phase Methanesulfonic Acid Production in the OH-Initiated Oxidation of Dimethyl Sulfide at Low Temperatures

**DOI:** 10.1021/acs.est.2c05154

**Published:** 2022-09-22

**Authors:** Jiali Shen, Wiebke Scholz, Xu-Cheng He, Putian Zhou, Guillaume Marie, Mingyi Wang, Ruby Marten, Mihnea Surdu, Birte Rörup, Rima Baalbaki, Antonio Amorim, Farnoush Ataei, David M. Bell, Barbara Bertozzi, Zoé Brasseur, Lucía Caudillo, Dexian Chen, Biwu Chu, Lubna Dada, Jonathan Duplissy, Henning Finkenzeller, Manuel Granzin, Roberto Guida, Martin Heinritzi, Victoria Hofbauer, Siddharth Iyer, Deniz Kemppainen, Weimeng Kong, Jordan E. Krechmer, Andreas Kürten, Houssni Lamkaddam, Chuan Ping Lee, Brandon Lopez, Naser G. A. Mahfouz, Hanna E. Manninen, Dario Massabò, Roy L. Mauldin, Bernhard Mentler, Tatjana Müller, Joschka Pfeifer, Maxim Philippov, Ana A. Piedehierro, Pontus Roldin, Siegfried Schobesberger, Mario Simon, Dominik Stolzenburg, Yee Jun Tham, António Tomé, Nsikanabasi Silas Umo, Dongyu Wang, Yonghong Wang, Stefan K. Weber, André Welti, Robin Wollesen de Jonge, Yusheng Wu, Marcel Zauner-Wieczorek, Felix Zust, Urs Baltensperger, Joachim Curtius, Richard C. Flagan, Armin Hansel, Ottmar Möhler, Tuukka Petäjä, Rainer Volkamer, Markku Kulmala, Katrianne Lehtipalo, Matti Rissanen, Jasper Kirkby, Imad El-Haddad, Federico Bianchi, Mikko Sipilä, Neil M. Donahue, Douglas R. Worsnop

**Affiliations:** † Institute for Atmospheric and Earth System Research/Physics, Faculty of Science, University of Helsinki, 00014 Helsinki, Finland; ‡ Institute of Ion Physics and Applied Physics, University of Innsbruck, 6020 Innsbruck, Austria; § Institute for Atmospheric and Environmental Sciences, Goethe University Frankfurt, 60438 Frankfurt am Main, Germany; ∥ Center for Atmospheric Particle Studies, Carnegie Mellon University, Pittsburgh, Pennsylvania 15213, United States; ⊥ Laboratory of Atmospheric Chemistry, Paul Scherrer Institute, CH-5232 Villigen, Switzerland; ⬢ CENTRA and Faculdade de Ciências da Universidade de Lisboa, 1749-016 Campo Grande, Lisboa, Portugal; ∇ Leibniz Institute for Tropospheric Research, Permoserstrasse 15, 04318 Leipzig, Germany; ○ Institute of Meteorology and Climate Research, Karlsruhe Institute of Technology, 76344 Karlsruhe, Germany; ◆ Helsinki Institute of Physics, University of Helsinki, 00014 Helsinki, Finland; ¶ Department of Chemistry and Cooperative Institute for Research in the Environmental Sciences, University of Colorado Boulder, Boulder, Colorado 80309, United States; †† CERN, the European Organization for Nuclear Research, CH-1211 Geneva 23, Switzerland; ‡‡ Aerosol Physics Laboratory, Physics Unit, Faculty of Engineering and Natural Sciences, Tampere University, 33014 Tampere, Finland; §§ Division of Chemistry and Chemical Engineering, California Institute of Technology, Pasadena, California 91125, United States; ∥∥ Aerodyne Research, Inc., Billerica, Massachusetts 01821, United States; ⊥⊥ Atmospheric and Oceanic Sciences, Princeton University, Princeton, New Jersey 08540, United States; ## Department of Physics, University of Genoa & INFN, 16146 Genoa, Italy; ∇∇ Department of Chemistry, Carnegie Mellon University, Pittsburgh, Pennsylvania 15213, United States; ○○ Department of Atmospheric and Oceanic Sciences, University of Colorado Boulder, Boulder, Colorado 80309, United States; ◆◆ P.N. Lebedev Physical Institute of the Russian Academy of Sciences, 119991 Moscow, Russia; ¶¶ Finnish Meteorological Institute, Erik Palmenin aukio 1, 00560 Helsinki, Finland; ††† Division of Nuclear Physics, Lund University, 22100 Lund, Sweden; ‡‡‡ Department of Applied Physics, University of Eastern Finland, 70211 Kuopio, Finland; §§§ School of Marine Sciences, Sun Yat-sen University, 519082 Zhuhai, China; ∥∥∥ Institute Infante Dom Luíz, University of Beira Interior, 6200-001 Covilhã, Portugal; ### Joint International Research Laboratory of Atmospheric and Earth System Sciences, School of Atmospheric Sciences, Nanjing University, 210023 Nanjing, China; ∇∇∇ Aerosol and Haze Laboratory, Beijing Advanced Innovation Center for Soft Matter Science and Engineering, Beijing University of Chemical Technology, 100029 Beijing, China; ◆◆◆ Department of Chemical Engineering, Carnegie Mellon University, Pittsburgh, Pennsylvania 15213, United States; ¶¶¶ Department of Engineering and Public Policy, Carnegie Mellon University, Pittsburgh, Pennsylvania 15213, United States

**Keywords:** dimethyl sulfide (DMS), OH-initiated oxidation, methanesulfonic acid (MSA), methanesulfinic acid (CH_3_S(O)OH, MSIA), low temperatures

## Abstract

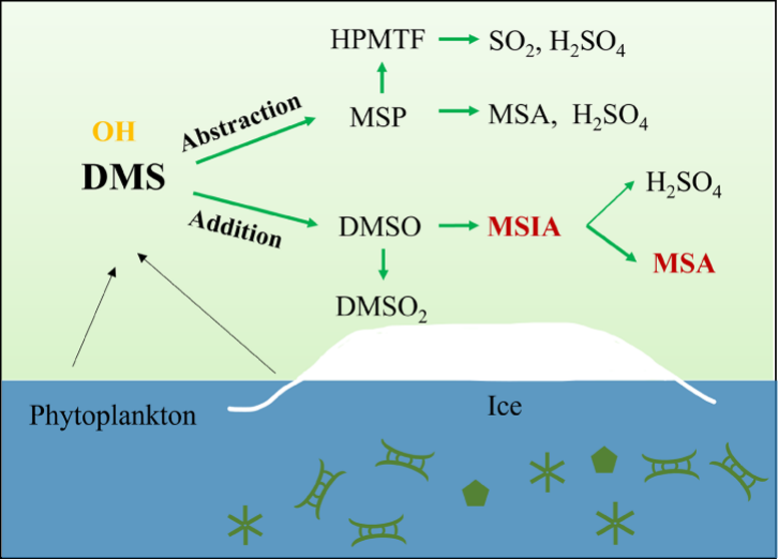

Dimethyl sulfide (DMS) influences climate via cloud condensation nuclei (CCN) formation resulting from its oxidation products (mainly methanesulfonic acid, MSA, and sulfuric acid, H_2_SO_4_). Despite their importance, accurate prediction of MSA and H_2_SO_4_ from DMS oxidation remains challenging. With comprehensive experiments carried out in the Cosmics Leaving Outdoor Droplets (CLOUD) chamber at CERN, we show that decreasing the temperature from +25 to −10 °C enhances the gas-phase MSA production by an order of magnitude from OH-initiated DMS oxidation, while H_2_SO_4_ production is modestly affected. This leads to a gas-phase H_2_SO_4_-to-MSA ratio (H_2_SO_4_/MSA) smaller than one at low temperatures, consistent with field observations in polar regions. With an updated DMS oxidation mechanism, we find that methanesulfinic acid, CH_3_S(O)OH, MSIA, forms large amounts of MSA. Overall, our results reveal that MSA yields are a factor of 2–10 higher than those predicted by the widely used Master Chemical Mechanism (MCMv3.3.1), and the NO*_x_* effect is less significant than that of temperature. Our updated mechanism explains the high MSA production rates observed in field observations, especially at low temperatures, thus, substantiating the greater importance of MSA in the natural sulfur cycle and natural CCN formation. Our mechanism will improve the interpretation of present-day and historical gas-phase H_2_SO_4_/MSA measurements.

## Abstract 2

Understanding the OH-initiated DMS oxidation process in the atmosphere is essential to properly describe the process chain from phytoplankton DMS emissions to climate effects.

## Introduction

Dimethyl sulfide (DMS) is emitted into the atmosphere by marine bacteria and as a result of the degradation of dimethylsulfoniopropionate (DMSP) produced from phytoplankton.^1−3^ These emissions are the most abundant biological source of sulfur,^3^ contributing between 18 and 42% of the global atmospheric sulfate aerosol.^4^ Sulfur-containing oxidation products from DMS—specifically, sulfuric acid (H_2_SO_4_) and methanesulfonic acid (CH_3_S(O)(O)OH, MSA) that formed in the gas phase and condensed phase—take part in the formation of sulfur-containing aerosols and cloud condensation nuclei (CCN) in the marine atmosphere.^3,5−7^ They initially form small (ca. nanometer-sized) molecular clusters through nucleation,^8−13^ after which they grow by further condensation to CCN sizes. They thus affect the formation, optical properties, and lifetime of marine clouds,^8^ influencing cloud radiative properties and therefore climate.^5,14−16^

The ratio of particulate MSA to non-sea-salt sulfate varies between 0.05 and 0.75 and is usually below 0.5.^6,7,17,18^ Understanding how gaseous MSA contributes to this ratio is important in understanding the impact of DMS oceanic emissions leading to the formation of low-volatility species via oxidation. The multiphase chemical mechanism is complex, and the yields of H_2_SO_4_ and MSA depend on temperature^19^ as well as atmospheric composition. Moreover, field measurements of gas-phase MSA and H_2_SO_4_ show a wide range of concentrations.^12,20−23^

Atmospheric MSA is formed primarily from DMS oxidation, with high concentrations observed in polar regions.^12^ Atmospheric H_2_SO_4_ is formed from sulfur dioxide (SO_2_) oxidation, which comes from anthropogenic and volcanic emissions as well as DMS oxidation. It follows that the ratio of H_2_SO_4_ to MSA (H_2_SO_4_/MSA) is an important indicator of both regional variations and anthropogenic perturbation in both current and historical samples. Furthermore, both species may participate in new particle formation^8−13^ and CCN formation processes together with iodine oxoacids and ammonia in polar regions.^24,25^ Consequently, a quantitative, mechanistic understanding of the DMS oxidation mechanism(s), especially the resulting H_2_SO_4_/MSA, is essential to further our understanding of their roles in climate change.

DMS oxidation has been studied extensively with a wide array of instrumentation. However, chamber and flow-tube experiments have had difficulties reproducing DMS oxidation under atmospherically representative conditions, leaving us with significant gaps in our chemical understanding.^26−28^ Recent experimental work and in situ field observations have shown that autoxidation plays an important role in the oxidation of DMS at higher temperatures (>0 °C), proceeding through the formation of hydroperoxymethyl thioformate (HPMTF; HOOCH_2_SCHO) via isomerization of the methylthiomethylperoxy radical (CH_3_SCH_2_OO, MSP), which is the primary product of DMS hydrogen abstraction.^29−32^ However, subzero conditions (<0 °C) are challenging to investigate experimentally and so have received less attention. Hydrogen abstraction, both by OH and via isomerization (autoxidation), is suppressed at a lower temperature, and the OH addition pathway is known to dominate, as shown in previous studies.^33−35^ Finally, wall reactions can be problematic in the laboratory, and their effects on the DMS oxidation experiments have been unclear so far.^27^

Model studies have addressed the importance of halogen chemistry^36−39^ and aqueous-phase processes^28,37,38,40^ to close the gaps between the modeled and measured oxidation products, suggesting reduced importance for OH-initiated DMS oxidation. However, even with recent revisions based on experimental findings, models with current state-of-the-art gas-phase mechanisms underestimate ambient MSA.^37,40^ Here, we show that those mechanisms overestimate H_2_SO_4_/MSA by a factor of 2–10 throughout the ambient temperature range. The mechanism adopted in this study is similar to the gas-phase mechanism in the study of Wollesen de Jonge et al.,^41^ which revised the gas-phase chemistry (MCM) to explain their high particulate MSA.

We performed experiments in the CLOUD chamber at CERN^13^ under conditions that closely match the marine boundary layer (MBL) with an extensive suite of instruments. We investigated the gas-phase OH-initiated DMS oxidation over a wide temperature range (−10 to +25 °C) and present a revised mechanism describing gas-phase DMS oxidation. In addition to H_2_SO_4_ and MSA, we quantified numerous intermediate products, including the key intermediate methanesulfinic acid (CH_3_S(O)OH, MSIA), and used them to evaluate and constrain the mechanism. In addition to the gas-phase chemistry, we quantified semiempirical rate coefficients for the heterogeneous formation of the important products dimethyl sulfoxide (CH_3_S(O)CH_3_, DMSO) and dimethyl sulfone (CH_3_S(O)_2_CH_3_, DMSO_2_). We designed our experiments such that the particle condensation sink (CS) is negligible. In CLOUD and most experiments, these multiphase processes occur on the chamber walls,^27,41^ but they can also occur on or within aerosols and cloud droplets in the atmosphere. We investigated the effects of temperature and NO*_x_* on gas-phase OH-initiated DMS oxidation. Both our experiments and our revised oxidation mechanism closely match ambient gas-phase H_2_SO_4_/MSA, and together they provide a quantitative understanding of this critical natural biogeochemical process.

## Materials and Methods

### CLOUD Chamber and Experiments

The CLOUD chamber is an electropolished stainless steel cylinder with a volume of 26.1 m^3^. The chamber is surrounded by an insulated thermal housing, which maintains high-temperature uniformity and stability, and it can be operated over a wide range of temperatures (from −70 to +100 °C) and relative humidity (from below 1% to above 90%). Ultrapure synthetic, humidified air (cryogenic 79% N_2_ and 21% O_2_) and a slight overpressure minimize contaminants in the chamber. All trace gases (e.g., SO_2_, O_3_, CO, NH_3_, NO*_x_*, and DMS) have independent gas lines connected to the chamber to avoid reactions caused by mixing in the gas lines before injecting into the chamber. Dry nitrogen was used to dilute DMS, NO_2_, and O_3_ from standard high-concentration gas bottles before reaching the chamber to obtain close to atmospheric concentrations.

Four 200 W Hg–Xe UV lamps (UVH LC8, Hamamatsu Photonics K.K., Japan) at wavelengths between 250 and 450 nm with adjustable power and a xenon fluoride excimer laser (UVX) at 248 nm were used to generate OH radicals via photolysis of ozone (O_3_). The distribution of UV lamps and laser in the chamber make the gas-phase oxidation products (e.g., OH) form uniformly in a few minutes. An LED sabre (LS3) at 385 nm was used to photolyze NO_2_ producing NO. Sulfur dioxide (SO_2_) was measured with a high-sensitivity pulse fluorescence analyser (model 43i-TLE; Thermo Fisher Scientific), ozone with a UV photometric ozone analyser (model 49C; Thermo Environmental Instruments), carbon monoxide using a nondispersion cross-modulation infrared analysis method (model APMA-370; Horiba), and nitrogen oxides NO with an ECO Physics CLD 780TR and NO_2_ with a Cavity Enhanced Differential Optical Absorption Spectroscopy (CE-DOAS). The SO_2_ monitor employed at CLOUD was unfortunately not able to measure the formed SO_2_ from DMS oxidation because of its high detection limit (0.5 ppb). A chilled dew-point mirror (EdgeTech Instruments) and a direct tunable diode laser absorption spectrometer (TDL hygrometer, Werle et al.^42^) were used to continuously monitor the water vapor concentration.

The OH-initiated DMS oxidation experiment was conducted as follows: Before the oxidation experiments, the mixing fan speed was set to 100% to enhance turbulent mixing in the chamber, thus reducing the concentrations of oxidation products due to faster wall loss. The injection rates of ozone, DMS, and NO*_x_* were set to reach the desired values. The experiment was then initiated by switching the UV lights on and setting the fan speed to 12% to initiate DMS oxidation (by OH radicals) and to reduce wall loss rates of DMS oxidation products to establish a new steady-state condition. Temperature-ramping experiments were conducted at two temperature ranges (+25 to +10 and +10 to −10 °C) with different initial concentrations of DMS, OH, and CO. The temperature ramps took a few hours each, which is only slightly longer than the residence time in the chamber. Therefore, unlike in a typical oxidation experiment, steady states were not reached for each temperature in the temperature-ramping experiments. As such, H_2_SO_4_-to-MSA ratios in these experiments should not be used directly in other contexts because they are not measured in steady state. They can, however, be used for investigating the temperature dependence by simulating the full concentration profile at each point in time and then comparing the model results with the experimental results.

### Measurements of DMS and its Oxidation Products

State-of-the-art instruments were operated simultaneously to measure the gas-phase concentrations of DMS and its oxidation products. They were measured by a suite of advanced mass spectrometers including a nitrate-ion-based chemical ionization mass spectrometer (NO_3_^–^-CIMS), a bromide chemical ionization mass spectrometer coupled with a multischeme chemical ionization inlet (Br^–^-MION-CIMS), the gas-phase channel of a bromide chemical ionization mass spectrometer equipped with a Filter Inlet for Gases and AEROsols inlet (Br^–^-FIGAERO-CIMS), selective reagent ionization mass spectrometers (SRI-TOF-MS), and proton-transfer reaction time-of-flight mass spectrometer (H_3_O^+^-CIMS, NH_4_^+^-CIMS). The experiments were grouped into three sets depending on the availability of instruments and temperature. We conducted experiment set 1 at −10 °C, experiment set 2 at +10 and −10 °C, and experiment set 3 at +25 and +10 °C, with relative humidity ranging from 20 to 70%. NO_3_^–^-CIMS and PTR3 worked during all of the experiments. However, the mode of PTR3 was changed from a regular H_3_O^+^ mode to NH_4_^+^ mode in experiment set 3. Br^–^-MION-CIMS measurements were only available in experiment set 1 and Br^–^-FIGAERO-CIMS measurements were only in experiment set 2. The conditions of the experiments and relevant instruments are listed in Table S1.

### DMS

A proton-transfer reaction time-of-flight mass spectrometer (H_3_O^+^-CIMS) provided the DMS concentration. The instrument is based on the design of the proton-transfer reaction time-of-flight mass spectrometer (PTR3) described in Breitenlechner et al.^43^ DMS concentrations were calibrated with a gas standard at specific conditions between experiments. The concentrations of DMS for the second experiment set were provided by the selective reagent ionization mass spectrometer (SRI-TOF-MS) described in detail by Canaval et al.^44^ We calibrated both instruments regularly between experiments with a standard gas mixture containing multiple volatile organic compounds to account for any possible drifts in transmission efficiency or absolute humidity dependency.

### H_2_SO_4_, MSA, and CH_3_S(O)_2_OOH

The concentrations of H_2_SO_4_, MSA, and CH_3_S(O)_2_OOH were measured with a nitrate-ion-based chemical ionization mass spectrometer (NO_3_^–^-CIMS; Tofwerk AG, Thun, Switzerland; Jokinen et al.^45^). The specially designed inlet for chemical ionization at the ambient pressure system and its calibration and quantification procedures are well described by previous studies.^46,47^ We applied the same calibration coefficient *C*_H_2_SO_4__ = 4.13 × 10^10^ cm^–3^ per normalized signal (cps cps^–1^; cps denotes counts per second) for the experiments carried out at +10 and −10 °C since charging efficiency does not vary significantly in this temperature range. The uncertainty for NO_3_^–^-CIMS is mainly caused by H_2_SO_4_ calibration, below ∼50%; this uncertainty includes the systematic error from calibration setup and statistic error. All uncertainties for each species from the different instruments are listed in Table S2. We estimate that MSA has a collision-limited charging efficiency and strong binding energy with less fragmentation than H_2_SO_4_ based on the cluster binding enthalpy calculated by quantum chemical methods (see Text S1.3 and Tables S3 and S4 for details). The measurement sensitivity of methanesulfonic peroxide (CH_3_S(O)_2_OOH) is lower than the maximum sensitivity, and the given concentrations represent its lower limits. The detailed estimation for MSA and CH_3_S(O)_2_OOH can be found in Text S1.3.

### DMSO, DMSO_2_, CH_3_SCHO, and CH_3_SOH

The concentrations of DMSO, DMSO_2_, methyl thioformate (CH_3_SCHO), and methanesulfenic acid (CH_3_SOH) were measured either by an H_3_O^+^- or NH_4_^+^-CIMS.^48^ Different ionization schemes (H_3_O^+^- or NH_4_^+^-chemical ionization) were used in different experiments as shown in Table S1. We determined the instrument’s collision limit calibration factor by ionizing 1 ppbv of hexanone from a gas standard diluted in air. Applying the collision limit calibration factor to all compounds ensures lower-limit estimates for their concentrations. As shown in Figure S2, the independence of the concentration ratios determined by both ionization methods (H_3_O^+^- and NH_4_^+^-chemical ionization) with the collision limit calibration factor on sample gas humidity and temperature suggests that both DMSO and DMSO_2_ are very likely ionized at the collision limit in both ionization modes. The lower-limit estimates are therefore most likely their true concentrations. The comparison between the two ionization methods at the same time can be realized with additional experiments performed with high ammonia concentration in the chamber. These additional experiments have much higher DMS, O_3_, and NH_3_ concentrations than the OH-initiated DMS oxidation experiments to investigate the formed particles at larger sizes of around 100 nm. In contrast to the OH oxidation experiments whose goal is to investigate the chemical mechanism, the goal of the additional experiments with high NH_3_ is to investigate the CCN potential of the formed particles. That is, the much higher concentrations of DMS, O_3_, and NH_3_ facilitate sustained condensation that leads to larger particles. Additionally, the conducted voltage scan experiment in Figure S3 shows that the DMSO and DMSO_2_ hydronium-water and ammonium clusters are stable against fragmentation at the chosen settings and that their proton affinity is high enough to even keep the charge upon collision-induced fragmentation at higher voltages. Their uncertainties are estimated at around 20% in H_3_O^+^-CIMS and 50–80% in NH_4_^+^-CIMS with the larger uncertainty of the collision limit sensitivity in the latter. The reported concentrations of CH_3_SCHO and CH_3_SOH are lower-limit estimates also based on the results of the voltage scans. See Text S1.4 for the detailed description.

### HPMTF and MSIA

A bromide chemical ionization mass spectrometer coupled with a multischeme chemical ionization inlet (Br^–^-MION-CIMS) and the gas-phase measurement of bromide chemical ionization mass spectrometer equipped with a Filter Inlet for Gases and AEROsols (Br^–^-FIGAERO-CIMS)^49^ were the primary instruments to detect MSIA and HPMTF. The peaks of MSIA and HPMTF measured by Br^–^-MION-CIMS can be found in Figure S4. If neither instrument was available due to instrument malfunction or absence in some experiments, NO_3_^–^-CIMS was used to measure MSIA and HPMTF. The instrument setup and operation of Br^–^-MION-CIMS and FIGAERO are described in Rissanen et al.^50^ and Lopez-Hilfiker et al.,^49^ respectively. In addition to ionization with Br^–^, we also detected both MSIA and HPMTF with NH_4_^+^ and H_3_O^+^-CIMS. While direct calibrations of MSIA and HPMTF were not available, the intercomparison between the three ionization modes and the use of quantum chemical calculations provide reasonable constraints on the ionization efficiency of the two species and hence their concentrations.

We first derived MSA calibration coefficients for Br^–^-MION-CIMS and Br^–^-FIGAERO_(g)_-CIMS using the MSA concentration measured by NO_3_^–^-CIMS. Afterward, MSA calibration factors in both instruments are used to estimate the lower limits of HPMTF and MSIA concentrations with high uncertainty. The traces shown in Figure S7 for MSIA from NH_4_^+^- and H_3_O^+^-CIMS are calibrated using the collision limit assumption. It is very likely that if ionization does not occur at the collision limit with all primary ion clusters with water, a strong water dependence would be observed between the different modes. The apparent humidity independence of the ratio of the MSIA concentration derived from the three different ionization schemes (NH_4_^+^, H_3_O^+^, and Br^–^) suggests that the compound is detected at the collision limit in these ionization schemes. However, the fragmentation caused by the electric field in the vacuum chamber also affects the detection efficiency. The signals of MSIA and HPMTF in Br^–^-MION-CIMS might underly some fragmentation or other unaccounted losses in the instrument or inlet. Yet, the voltage scan results in Figure S8 show that MSIA is detected at maximum sensitivity in both H_3_O^+^ and NH_4_^+^-CIMS. Therefore, we use the MSIA concentration from H_3_O^+^-CIMS with around 28% uncertainty due to the calibration and inlet and instrumental loss correction. The signal of HPMTF is influenced by neighboring peaks and is divided into many water clusters in H_3_O^+^-CIMS, while this is not the case in NH_4_^+^-CIMS. Unfortunately, NH_4_^+^-CIMS was not available for many of the experiments, and the limit of detection in H_3_O^+^-CIMS was too high to use the data during the herein analyzed experiments with low concentrations. As such, we use the lower-limit estimation for HPMTF concentrations from Br^–^-FIGAERO_(g)_-CIMS. See Section S1.5 for the detailed description and estimations.

### Steady-State Measurement and Production Rate Calculation

The average residence time in the CLOUD chamber was ∼1.3 h for experiment sets 1 and 2. The reaction rates of oxidation products were derived from steady-state measurements, which means that the inflow of all gases, the concentration of oxidants, and oxidation products were kept constant. Under steady-state conditions, the rate of change of oxidation products equals zero, and we can write
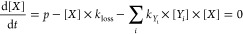
1

Therefore, we can get the production rate *p*
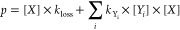
2where *X* is the oxidation product, *Y* typically is OH or O_3_, *k*_*Y*_i__ are the reaction rate coefficients for the reaction of *X* and *Y*, and *k*_loss_ is either the ventilation loss (2.1 × 10^–4^ s^–1^ in experiment sets 1 and 2) or wall loss that is listed in Table S2. All of the oxidation product concentrations were measured at the steady-state condition. The OH concentration used in this study is estimated from our box model, and its reactions involved in forming OH radicals are listed in Table S7.

### Modeling

In this study, a numerical model with zero dimension was set up and used to simulate DMS oxidation processes at different initial conditions (Table S1). We combine the simulation and experimental results to evaluate and constrain the role of different DMS gas-phase oxidation pathways in MSA and H_2_SO_4_ formation. The chemistry mechanism was generated with the Kinetic PreProcessor (KPP)^51^ and solved with the ordinary differential equation solver RODAS3 (Rosenbrock method of order 3).^52^ The concentrations of oxidation products are determined by the chemical production and loss from gaseous chemistry, wall reactions, ventilation loss, wall loss, and the condensation sink (CS) to generate particles in the CLOUD chamber. The measured temperature, relative humidity, DMS, O_3_, CO, NO*_x_* concentrations (Table S1), and O_3_ photolysis rate with high time resolution were used as input to the model. The photolysis rate of O_3_ for UVH at full intensity or 5 W of UVX is around 6 × 10^–5^ s^–1^. The OH concentrations are adjusted by changing the UV light intensity, O_3_, and H_2_O concentrations. The CLOUD chamber ventilation loss is mainly determined by the flush-out flow (∼330 lpm in experiment sets 1 and 2, 250 lpm in experiment set 3). Species like H_2_SO_4_, MSA, and hydrogen oxide radicals are readily lost to the wall surface. We measured the lifetime of all species (Table S2) in the dark decay (lights off, fan 12%) and cleaning stage (lights off, fan 100%) to estimate the losses and applied them to our box model. With the increase of fan speed from 12 to 100%, the wall losses increased by a factor of 1.5–4.6 for different species. The measured loss of species like methyl thioformate (CH_3_SCHO) in the cleaning stage is slightly larger than the ventilation loss caused by the flush-out flow. It suggests that the loss of CH_3_SCHO is dominated by ventilation whose loss increases mildly with the increased fan speed. Therefore, we added extra loss terms for dimethyl sulfoxide (CH_3_S(O)CH_3_, DMSO), dimethyl sulfone (CH_3_S(O)_2_CH_3_, DMSO_2_), and CH_3_SCHO to fit their time evolutions in the cleaning stage. The particle condensation sink measured in this study is smaller than 1 × 10^–5^ s^–1^, which is negligible compared to other loss processes. The detailed description of chemistry mechanisms used in the box model can be found in Text S2.

## Results

### Identification of Oxidation Products from OH-Initiated DMS Oxidation

**Figure 1:**
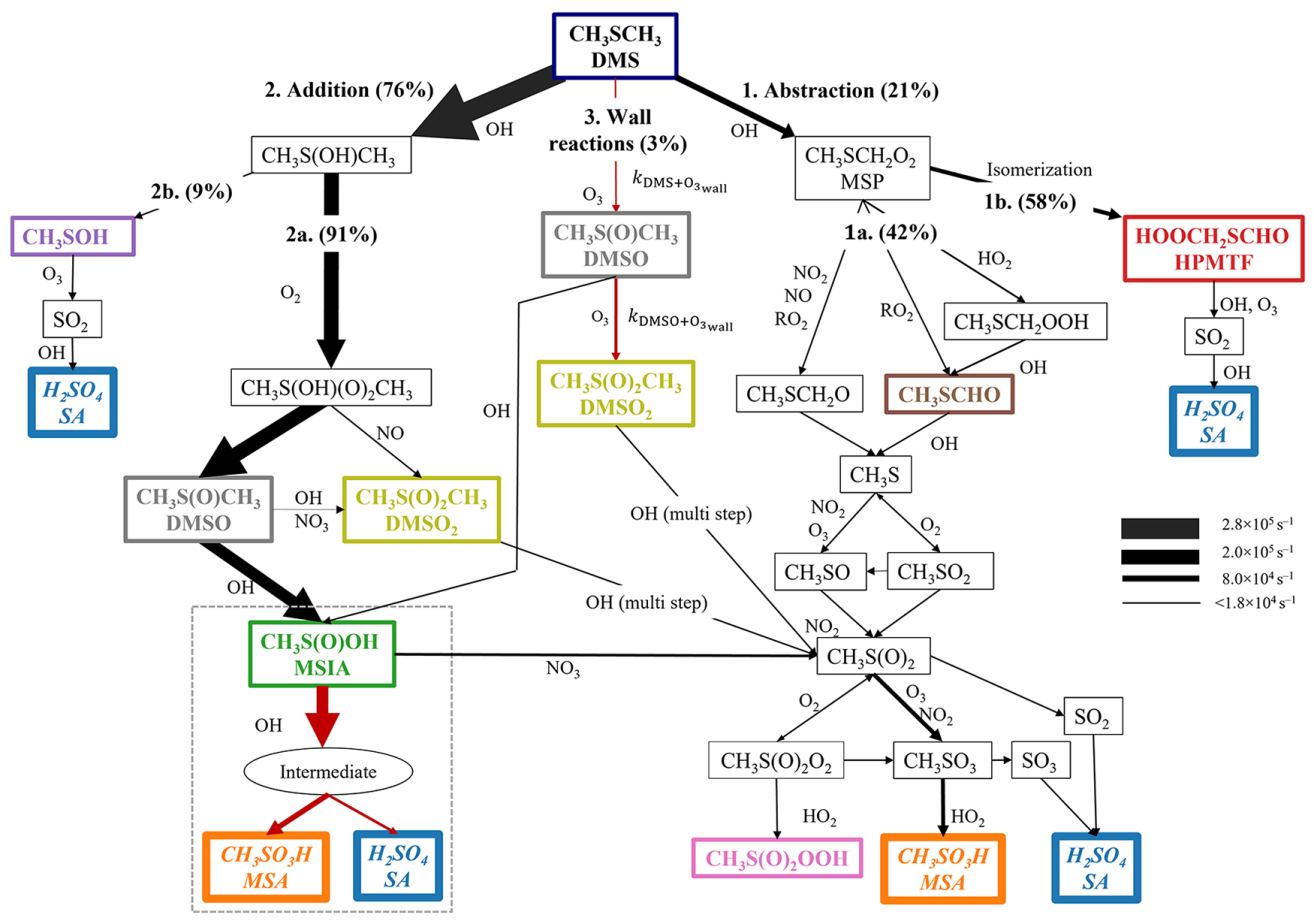
Schematic representation of DMS oxidation with OH radicals in this study. Most reactions are taken from MCMv3.3.1, Hoffman et al., and recent publications.^29,31,32,41^ The widths of the arrows indicate production rates (s^–1^) on a linear scale, which arecalculated at −10 °C with 100 pptv DMS, 7 × 10^6^ cm^–3^ OH, 40 ppbv O_3_, 2 pptv NO, and 200 pptv NO_2_. We set the same width for reaction rates when they are below 1.8 × 10^4^ s^–1^. The percentage given for each pathway indicates the branching ratio of the production rates at this condition. The precentage of MSP leads to pathways 1b is temperature-dependent. At +22 °C, the reaction follows pathway 1b to 97%. The reaction rate coefficients of the isomerization of MSP are discussed in Text S2. Red arrows highlight the important reactions proposed in this study. The mechanism of MSIA reacts with OH surrounded by the thin, dashed gray line is the possible alternative formation mechanism we suggest but their reaction rate coefficients are not fully studied. Therefore, we treat the MSIA converting to CH_3_S(O)_2_ in our box model. *k*_DMS + O_3wall__ and *k*_DMS + O_3wall__ are the semiempirical reaction rate coefficients for heterogeneous wall reactions (see Text S4 for details). The species in colored boxes in this figure are those we quantify, and the color of each box outline matches the color of each time trace in Figure 2. Species CH_3_S(OH)CH_3_, CH_3_S(O_2_)(OH)CH_3_, CH_3_SCH_2_O, CH_3_SO, and CH_3_SO_2_ have not been measured before but are included here based on modeling studies.^33,35,40^

**Figure 2:**
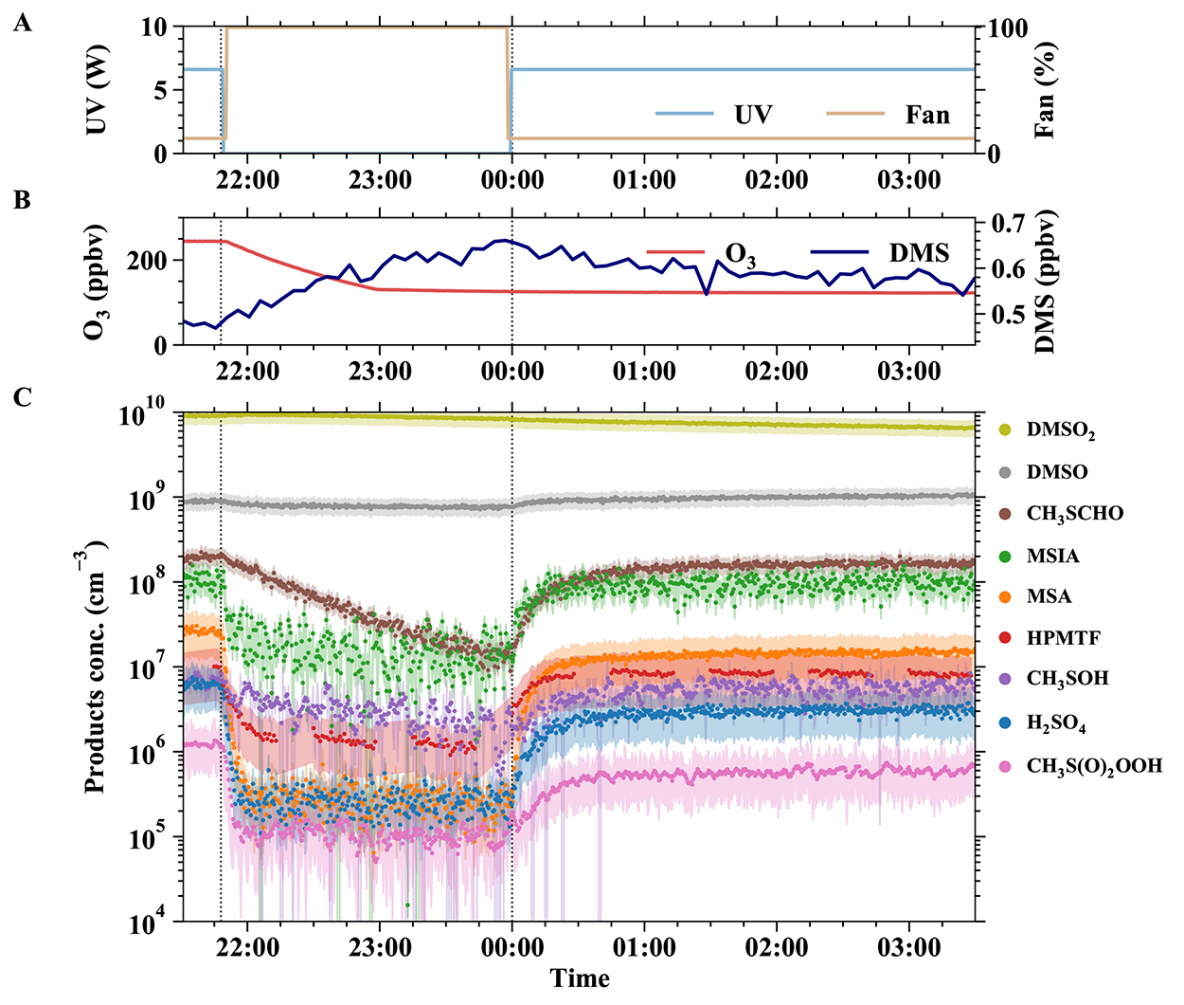
Experiment of OH-initiated DMS oxidation at −10 °C. (A) Time series of ultraviolet light intensity (blue line, left axis; used to photolyze O_3_ to produce OH radicals) and mixing fan speed to accelerate wall losses (orange line, right axis). (B) O_3_ (red line, left axis) and DMS (navy line, right axis) concentrations. (C) Measured DMS oxidation products (solid dots, colors correspond to box outlines in Figure 1). After a brief period ended at 21:48, UV lights were turned off and the fan speed was increased to full intensity to encourage wall losses. Most species were removed efficiently and showed a sharp drop. However, the loss of CH_3_SCHO was mainly due to the chamber ventilation (with a dilution lifetime of 1.3 h). DMSO_2_ was relatively insensitive to fan change since it was primarily produced from heterogeneous production on the walls, while DMSO was less affected by multiphase reactions (primarily from gas-phase reactions). When the fan speed was lowered and UV lights were turned on, the concentrations of most species increased due to OH oxidation of DMS. The shade for each time series presents the uncertainty of each species except for the species (HPMTF, CH_3_S(O_2_)OOH, and CH_3_SOH) whose concentrations are lower-limit estimates; their uncertainty only includes the instrumental loss without correction from sensitivity.

Figure 1 shows an oxidation mechanism initiated by the DMS + OH reaction and informed by the experiments we describe here, and Figure 2 shows an example time series of measured species for an experiment conducted at −10 °C with NO*_x_* below the instrument detection limit, ≲2 pptv. With sensitive chemical ionization mass spectrometers, we are able to identify and quantify many intermediates and terminal products in this mechanism. The species we measure include MSA and H_2_SO_4_, as well as DMSO, DMSO_2_, MSIA, and HPMTF. These are the major sulfur-containing products also previously identified and measured from DMS oxidation.^26,27,29,32,53^ We also identify CH_3_S(O)_2_OOH, CH_3_SCHO, and CH_3_SOH, as shown in the figures. Some species are quantified with proper calibration factors, while species like CH_3_S(O)_2_OOH, HPMTF, CH_3_SOH, and CH_3_SCHO (by NH_4_^+^-CIMS) are lower-limit estimates due to the lack of authentic standards or generation methods. A more detailed description is given in the Materials and Methods section and Text S1.

The time traces in Figure 2 show the sequence of a typical experiment, starting with the characterization of wall losses in the dark, followed by the initiation of photochemistry. UV lights, the trigger of OH radicals, were switched on at midnight (00:00) after DMS and ozone (O_3_) concentrations stabilized. The time sequences of different DMS oxidation products show different behaviors. The evolution of H_2_SO_4_, MSA, MSIA, HPMTF, CH_3_S(O)_2_OOH, and CH_3_SOH is consistent with the expected time response of gas-phase production through OH oxidation (with lights on) and wall loss. The measured concentration reaches their maxima in the order of a few minutes rather than a few hours like aqueous-phase reactions. The CH_3_SCHO evolution is consistent with gas-phase production and loss to ventilation rather than the walls, which has a timescale of ∼1.3 h (26.1 m^3^/330 lpm). DMSO shows a small fractional, but large absolute, increase with photochemistry, yet only a modest fractional wall loss. DMSO rapidly reaches a steady state, while DMSO_2_ decreases slowly and continuously from the onset of the run, associated with the slowly decreasing product of the DMS and O_3_ concentrations.

### Mechanism Treatment via the Box Model

Figure S11 reveals that DMSO and DMSO_2_ have a source from the wall, which has also been shown in a previous study.^27^ As described in the Methods section, we first constrain wall effects to delineate gas-phase chemistry; however, the gas-phase photochemistry is dominant for all species other than DMSO_2_ when the UV lights are on. In turn, DMSO_2_ is long-lived in the gas phase and so has minimal influence on the chemistry. DMSO is also produced on the walls, but once OH is present, both gas-phase production and loss of DMSO via OH far exceed the wall production rate (see Text S4 and Figure S11).

As shown in Figure 2, the terminal products MSA and H_2_SO_4_ are far less abundant than the first-generation oxidation product CH_3_SCHO, but MSA and H_2_SO_4_ have rapid wall loss, as do their precursors, such as MSIA, CH_3_SOH, and HPMTF. Considering the wall loss rate, we find that the production rates for MSA (3.2 × 10^4^ s^–1^) and CH_3_SCHO (4.5 × 10^4^ s^–1^) are similar, with uncertainties at around 50 and 20%, mainly caused by their measured concentrations. We seek these production rates along with molar yields and branching ratios. To constrain the production rates, we need a photochemical model of the chamber and the DMS oxidation sequence, including the walls.

The measured wall losses in Table S2 show that MSA and H_2_SO_4_ have the same values, allowing a direct comparison of the two species’ concentrations. As shown in Figure 2, the measured MSA is high and 3.7 times larger than H_2_SO_4_, which cannot be explained when assuming that MSA is exclusively formed from the hydrogen abstraction channel (pathway 1 in Figure 1). We observe a positive relationship between MSIA and MSA, shown in Figures S12 and S13. Previous studies^54,55^ have concluded that SO_2_ is the lone major product of the reaction of OH with MSIA. However, the abstraction of an acidic H-atom by OH radicals is typically slow because acidity implies a deficiency in electron density.^56^ By contrast, OH addition to the S-atom in MSIA is more likely to produce an intermediate product that may decompose to form sulfurous acid (H_2_SO_3_) and CH_3,_^55^ but may also react with O_2_ to produce MSA. This pathway and its reaction rate coefficients are not yet well understood. Therefore, in the box model, we add the pathway proposed by Yin et al.,^57^ Lucas et al.,^58^ and Hoffmann et al.^40^ that MSIA reacts with OH or NO_3_ radicals forming methyl sulfonyl radicals (CH_3_S(O)_2_) as an intermediate, which then either decomposes to SO_2_ or reacts with O_3_ to form MSA (via CH_3_SO_3_ reaction with HO_2_) and SO_3_, instead of producing SO_2_ only. A recent study also applied this pathway to a multiphase model^41^ to explain the observed high particulate MSA/sulfate at 0 and 20 °C.

Lv et al.^55^ suggested that the reaction of MSIA and O_3_ is unlikely to be competitive with MSIA and OH because of the large discrepancy between these two reaction rate coefficients. Even at high O_3_ concentrations (ca. 100 ppbv), the first-order reaction rate coefficient of MSIA by O_3_ is 4.4 × 10^–10^ s^–1^, which is much smaller than the first-order reaction rate coefficient of MSIA by OH at 2.8 × 10^–4^ s^–1^ (with lowest OH concentrations, ∼1 × 10^6^ cm^–3^). Therefore, the critical conclusion is that we would suggest that MSIA reacts with OH, producing both MSA and H_2_SO_4_. Thus, both the addition and abstraction pathways of DMS oxidation form MSA in the gas phase, explaining the higher MSA concentrations observed in field observations and CLOUD experiments.

**Figure 3:**
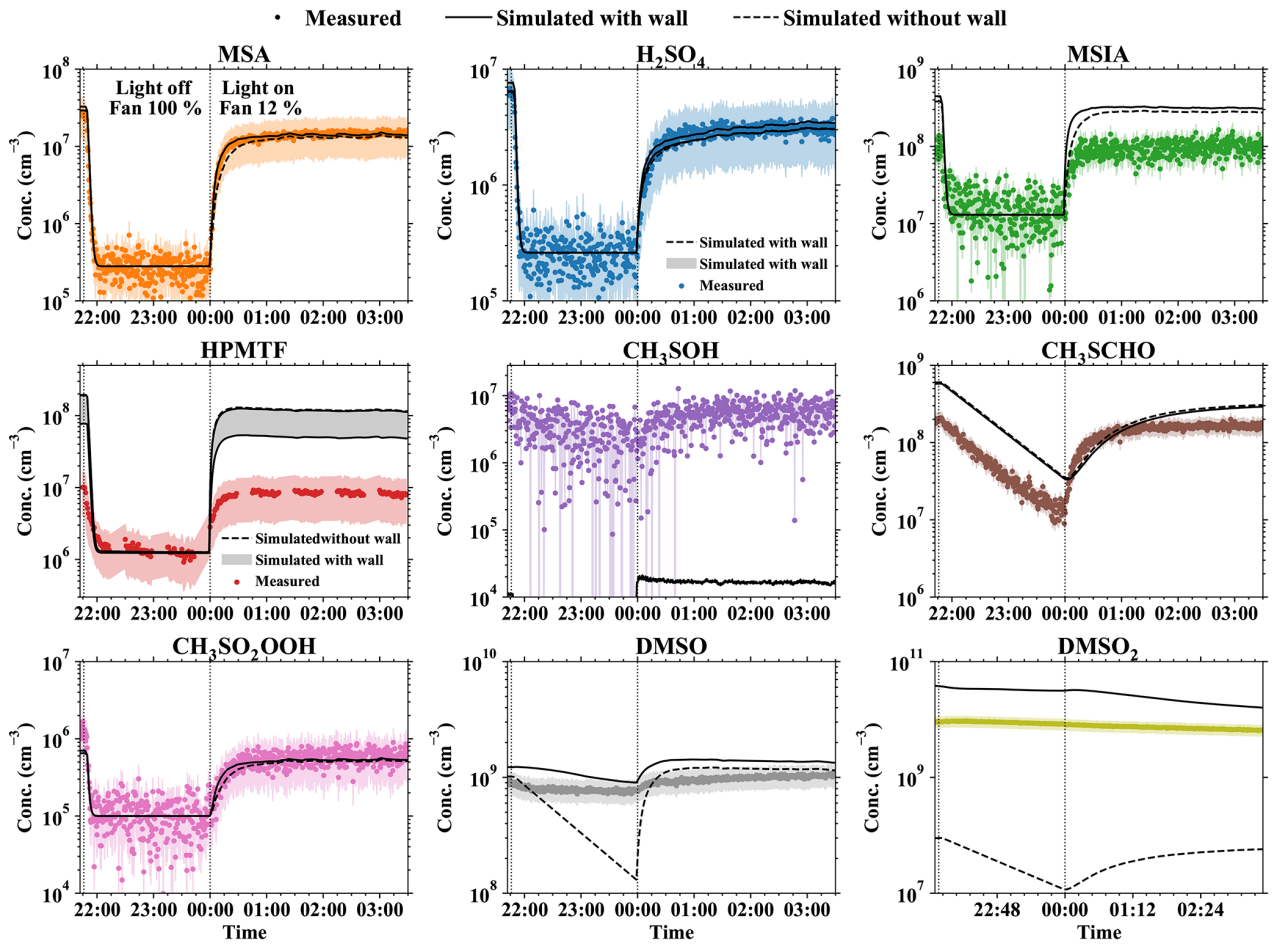
Measured and modeled gas-phase concentrations of identified species at an OH-initiated DMS oxidation experiment. Circles are the identified species measured by NO_3_^–^-CIMS, H_3_O^+^-CIMS, and Br^–^-FIGAERO_(g)_. Solid lines represent simulation results, including wall reactions of DMSO and DMSO_2_ and dashed lines represent simulation results excluding wall reactions. Wall loss is included in our box model; therefore, we can compare the measured and simulated values directly. The differences between the modeled and measured HPMTF and CH_3_SCHO are mainly because they are lower-limit estimates (see Texts S1.4 and S1.5 for quantitative measurement), which means their concentrations are underestimated in this case. The shadow area in the subplot of HPMTF presents the variation caused by applying different reaction rate coefficients of isomerization of MSP. Since the production rate of CH_3_SOH is unclear, the simulated CH_3_SOH concentrations are much lower than the measured values. It is a coincidence for the agreement between the measured and simulated CH_3_SO_2_OOH concentrations (lower-limit estimates), but we do not investigate it in detail because it is not important for understanding MSA and H_2_SO_4_ formation. The shade for each time series presents the uncertainty of each species except for the species (HPMTF, CH_3_S(O_2_)OOH, and CH_3_SOH) whose concentrations are lower-limit estimates; their uncertainty only includes the instrumental loss without correction from sensitivity.

To treat wall effects, we add wall reactions as pathway (3) to our DMS oxidation mechanism (Figure 1) by directly applying two semiempirical rate coefficients *k*_DMS + O_3wall__ and *k*_DMSO + O_3wall__. Using a numerical model (see Material and Methods for the configuration and mechanism), we then evaluate the proposed mechanism herein. We compare the measured oxidation products (circles) with their modeled values, including wall reactions (solid lines) in Figure 3 for the same OH-initiated DMS oxidation experiment as Figure 2.

The modeled MSA, H_2_SO_4_, CH_3_S(O)_2_OOH, and DMSO concentrations in Figure 3 are within 50% uncertainty of the measured values. The time evolution of HPMTF and MSIA in the simulation follows the same trend as the measured values. The modeled MSIA concentrations exceed the measured value (within an uncertainty of 28%) by a factor of 3, which is believed to be reasonable due to the uncertainties in DMS chemistry, chamber loss parameterizations, and systematic measurement errors. The smallest discrepancy between the modeled and measured HPMTF is a factor of 6. This difference can be explained by the uncertainty and the reduced sensitivity of the gas-phase measurement of Br^–^-FIGAERO-CIMS to HPMTF due to a stronger declustering process compared to the maximum sensitivity compounds (see Text S1.5 for details). The discrepancy between the modeled and measured HPMTF can be varied from 6 to 14 by applying different reaction rate coefficients of the isomerization of MSP. As shown in Figure 3, the variation of HPMTF at low temperatures has a limited effect on DMS oxidation.

When we exclude the wall reaction from the model, the resulting vast difference between the modeled and measured DMSO_2_ concentrations and their qualitative behaviors (Figure 3) indicates that wall reactions are essential to understanding the DMSO_2_ formation. On the other hand, the simulation without wall interactions captures the time evolution of the other species except for DMSO, which indicates that OH-initiated DMS oxidation is their dominant source, and DMSO_2_ is unlikely to be their primary precursor. The model suggests that DMSO is formed for the most part from OH-initiated DMS oxidation in the gas phase but interacts strongly with the walls. The modeled DMSO concentrations are 54 and 46 pptv, with and without wall reactions, respectively. However, the temporal evolution of the concentration cannot be reliably predicted without wall interactions. As shown in Table S2, the uncertainties of measured MSA, H_2_SO4, MSIA, CH_3_SCHO, DMSO, and DMSO_2_ are below 50%. Although the uncertainties of HPMTF, CH_3_SOH, and CH_3_S(O)_2_OOH are high, their influences on our main conclusion—that MSA likely forms substantially via gas-phase oxidation of MSIA by OH—are negligible since they are not the determining species in this argument.

Overall, the underestimated calibration factor can explain the discrepancy between the modeled and simulated HPMTF. Based on the close agreement between the model and measurements for major oxidation products (DMSO, DMSO_2_, MSIA, MSA, and H_2_SO_4_), we conclude that the mechanism presented here can represent DMS oxidation by OH radicals in the marine atmosphere.

### Temperature Dependence for OH-Initiated DMS Oxidation

**Figure 4:**
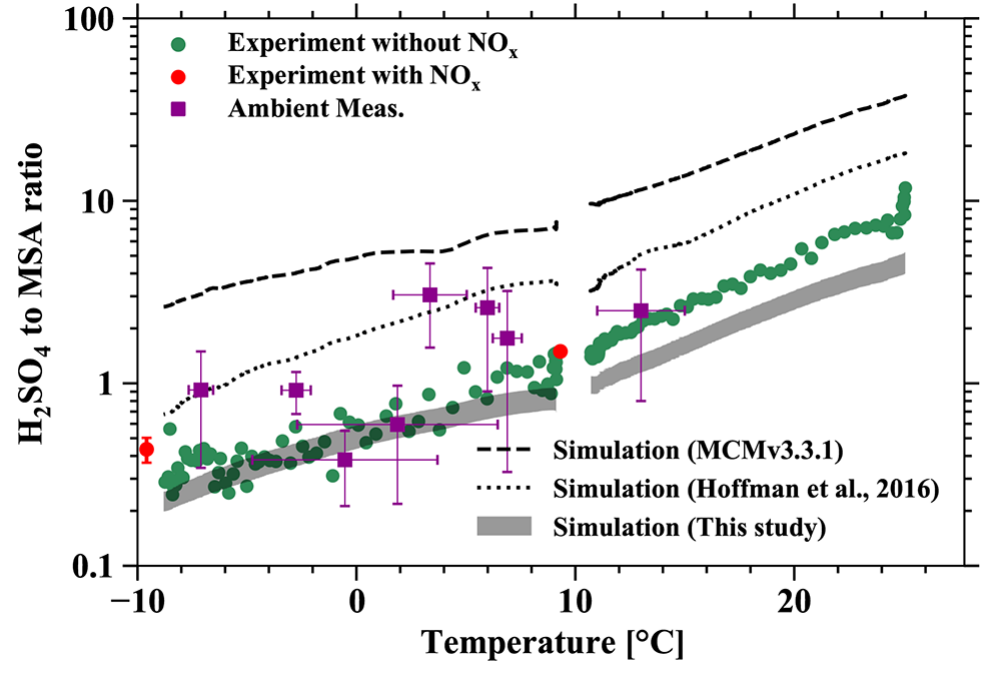
Temperature dependence of the H_2_SO_4_-to-MSA ratio. Green circles represent the H_2_SO_4_/MSA ratio without NO*_x_* (green) from two temperature-ramping experiments measured by NO_3_^–^-CIMS; red circles are the experimental H_2_SO_4_/MSA with NO*_x_*. The NO_2_ and NO differences between the red and green circles are around 400 and 8 pptv. The error bars represent the standard deviation of temperature and H_2_SO_4_/MSA. Purple square symbols are the daytime average of ambient measurement. They are the ambient measurements from 4 and 5 May, 5 and 6 August 2017 in Ny-Ålesund station, 20 August 2015 in Villum,^12^ 16 and 17 December 2014 in ABOA station,^68^ and 17 June 1999 in Mace head.^21^ We decreased the temperature to study the temperature dependence, in contrast to increasing the temperature, to avoid the emission of contamination from the wall. Lines represent the simulation results using the OH-initiated gas-phase oxidation mechanism from MCMv3.3.1 (dashed line, with constant NO*_x_* concentration) and Hoffman et al.^40^ (dotted line). The simulation results in this study are presented as gray rectangles by varying the reaction rate coefficient of the isomerization of MSP.

Temperature has a strong effect on (1) the branching ratio of addition/abstraction at the first step of DMS oxidation by OH radicals, (2) thermal decomposition of CH_3_SO_2_ and CH_3_SO_3_ in abstraction pathway 1a, (3), degradation of MSIA (which is not fully understood), and (4) peroxy radical isomerization of MSP in abstraction pathway 1b. All of these, especially the thermal decomposition, influence H_2_SO_4_/MSA. We plot H_2_SO_4_/MSA versus temperature in Figure 4 to present the temperature dependence for OH-initiated DMS oxidation. The green and red circles are from our CLOUD experiments with and without NO*_x_*, respectively. The purple symbols are ambient data, and the black lines are box-model simulations with various mechanisms. The CLOUD data come from two separate temperature-ramping experiments, one cooling from +25 to +10 °C and the other from +10 to −10 °C, both without NO*_x_*. In Figure 4, H_2_SO_4_/MSA ranges from 0.3 to 1.3 (−10 to +10 °C) and 1.4 to ∼11 (+10 to +25 °C), decreasing exponentially with decreasing temperature. Ambient observations of gas-phase H_2_SO_4_/MSA vary from 1 to 17 at Mace Head, Ireland (during Summer)^21^ and from 0.3 to 3 in polar regions (from February to August).^12^ These observations can be reproduced by the inferred experimental temperature dependence. The ambient concentrations of DMS, O_3_, OH, and NO*_x_* during the measurements of H_2_SO_4_/MSA from Beck et al., presented in Figure 4, were, unfortunately, unknown and likely differed from our experimental conditions. The differences will inevitably influence the absolute MSA and H_2_SO_4_ concentrations reported in Beck et al.’s observation and our experiments. However, we find that the ratio of H_2_SO_4_/MSA is mainly determined by temperature (Figure 4) instead of precursor vapor concentrations, and therefore the H_2_SO_4_/MSA from our experiments is nonetheless used to compare with field observations. MSA formation has a stronger temperature dependence than H_2_SO_4_. A detailed discussion of the temperature dependence for all of the oxidation products is given in Text S5 and Figure S14.

We modeled the temperature dependence of H_2_SO_4_/MSA with the OH-initiated DMS oxidation mechanism developed here (solid line) and compare it to the gas-phase mechanism from MCMv3.3.1^59^ (dashed line) and Hoffmann et al.^40^ (dotted line) in Figure 4. These model results differ by up to an order of magnitude compared to the CLOUD measurements, but all show a similar exponential temperature dependence. This is because they all include thermal decomposition for CH_3_S(O)_2_ and CH_3_SO_3_ in abstraction pathway 1a, which contains an exponential temperature dependence that largely determines H_2_SO_4_/MSA. However, our mechanism shows much better agreement with the observed H_2_SO_4_/MSA compared to the other two mechanisms. This is because the reaction of MSIA with OH radicals contributes more to MSA production and consequently reduces H_2_SO_4_/MSA rather than only contributing to SO_2_ formation, as e.g., in MCMv3.3.1. Overall, the observed temperature effect suggests a potentially greater role for MSA in colder regions in important processes such as new particle formation and growth, as our mechanism and observations show that the H_2_SO_4_/MSA ratio is smaller than 0.5 when the temperature is below roughly +2 °C.

### Effect of NO*_x_* on OH-Initiated DMS Oxidation

In addition to temperature, NO*_x_* (i.e., NO_2_ and NO) influences OH-initiated DMS oxidation, for example, through bimolecular reactions between peroxy radicals (RO_2_) and NO. As shown in Figure 4, H_2_SO_4_/MSA from two experiments with NO*_x_* (red circles) agrees with the temperature-ramping experimental results (green circles), indicating that NO*_x_* has a negligible effect compared to the temperature sensitivity over this same range. The NO*_x_*-ramping experiments in Figure S15 confirm that NO_2_ and NO only slightly enhance H_2_SO_4_/MSA in contrast to the strong effect from temperature change. The measured H_2_SO_4_/MSA at +10 °C (Figure S15A) and −10 °C (Figure S15B) increase by 54 and 100% as NO_2_ increases from 0 to 400 pptv. The effect of NO*_x_* on HPMTF formation is not clear (Figure S15). We observe a slight drop of HPMTF (17%) and almost constant DMS in Figure S15A. However, Figure S15B shows a 100% increase in HPMTF and a 20% drop in DMS because DMS reacts with NO_3_ radicals. We cannot quantify the effect of NO*_x_* on HPMTF formation because of the interference from N_2_O_5_ formed from the reaction of O_3_ and NO_2_, which has a close molecular weight to HPMTF.

In MCMv3.3.1, the modeled H_2_SO_4_/MSA (Figure S16) is very sensitive to NO_2_ concentration varies dramatically as NO_2_ increases from 0 to 160 pptv. However, the modeled H_2_SO_4_/MSA from the other two mechanisms (Figure 4) are insensitive to NO*_x_*. In MCMv3.3.1, NO_2_ constrains the formation of MSA through the reaction of CH_3_SO with NO_2_, which forms CH_3_S(O)_2_, because it excludes the isomerization reaction of CH_3_SO_2_ producing CH_3_S(O)_2_ (Hoffmann et al.^40^). The isomerization reaction is estimated with a constant value of ∼1 s^–1^ in previous studies,^60,61^ which is higher than the bimolecular reaction rate of CH_3_SO with NO_2_ (∼1.2 × 10^–1^ s^–1^ with a maximum 400 pptv NO_2_), although it lacks the temperature dependence.

Overall, the suite of simultaneously measured key intermediates helped us to adjust the previous DMS oxidation schemes. Our mechanism has two major improvements. First, the influence of NO*_x_* on product distribution is greatly diminished compared to the MCMv3.3.1. Second, the key ratio H_2_SO_4_/MSA is substantially reduced (MSA is increased), with the absolute ratio and temperature dependence in the mechanism agreeing with both the experimental data and ambient observations.

## Discussion and Atmospheric Implication

DMS oxidation in the atmosphere is affected by temperature, NO*_x_*, the distribution of oxidants including OH, NO_3_, halogen compounds,^36,37,62^ and available water for multiphase oxidation in aerosols and droplets.^40,63^ In this study, we followed the kinetics of a suite of gas-phase reaction intermediates and final products in DMS oxidation experiments by OH to investigate the effects of temperature and NO*_x_*. Our results show a strong temperature dependence for DMS oxidation, especially the yields of oxidation products such as MSA.

Our experimental results show high MSA concentrations and reduced H_2_SO_4_/MSA from OH-initiated DMS oxidation, especially at low temperatures (<0 °C). From the box-model results, it is clear that the inclusion of the gas-phase MSA formation mechanism via oxidation of MSIA by OH improves the agreement between modeled and experimental results at low temperatures. When MSIA reacts with OH, it may produce CH_3_S(O)_2_ or another adduct intermediate; regardless, both CH_3_S(O)_2_ and the adduct intermediate decompose to SO_2_ or SO_3_ to form H_2_SO_4_ or react with O_3_ or O_2_ to form MSA. For both pathways, we can expect a similar temperature dependence.

At low temperatures, the oxidation of DMS with OH proceeds more via the OH addition channel, forming abundant DMSO and MSIA, and consequently, a large amount of MSA. At high temperatures, the reaction rate of the OH addition channel decreases, which lowers MSIA and MSA formation, while the hydrogen abstraction channel becomes more important. Also, the predominance of MSP isomerization (≥95, at 295 K)^29^ leads to the HPMTF formation, which increases H_2_SO_4_ production substantially through oxidation of HPMTF.^29,30^

Therefore, we propose that MSIA is important in MSA formation at low temperatures, which can also be connected to the observed high particulate MSA/sulfate in laboratory^41^ and field observations^7,17^ since both MSA and H_2_SO_4_ participate in particle growth. Interestingly, NO*_x_* is far less influential than previously thought. At −10 °C, H_2_SO_4_/MSA increases by 100% after ramping up NO_2_ (from 0 to 400 pptv) and NO (from 0 to 8 pptv). But the observed change (red circles) is small (less than 50%) compared to the effect of changing temperature (about an order of magnitude when the temperature decreases from +10 to −10 °C).

As shown in Table S5, our experimental MSA (0.2 to 1.5 × 10^7^ cm^–3^) and H_2_SO_4_ (0.3 to 3 × 10^6^ cm^–3^) concentrations formed from OH-initiated DMS oxidation experiments conducted at −10 °C are in the range of field measurements. For example, the concentrations of MSA and H_2_SO_4_ measured in Ny-Ålesund station (Figure S17, air temperature ranging from −10 to −7 °C) range from 0.1 to 4 × 10^7^ cm^–3^ and 0.2 to 7 × 10^6^ cm^–3^, respectively. Aerosol concentrations also affect gas-phase MSA and H_2_SO_4_ concentrations because of their different aerosol partitioning. However, the particle condensation sink in this study is smaller than 1 × 10^–5^ s^–1^, which is only slightly larger than the values at Ny-Ålesund (∼4 × 10^–4^ s^–1^) and Villum (∼3 × 10^–4^ s^–1^).

DMSO_2_ and DMSO are important sulfur reservoirs in our study. They can also be formed from wall reactions without OH radicals. Our simulations show that wall reactions are the major source of DMSO_2_ but have a limited role for other oxidation products compared to the gas-phase reactions (even DMSO). In the atmosphere, analogous to the wall of the chamber, cloud droplets and aerosol water could play a similar role. Therefore, the observed high concentrations of DMSO_2_ (40–120 pptv) and DMSO (3–18 pptv) at night over the Arabian Sea^64^ and Pacific Ocean^65^ may be associated with multiphase DMS oxidation.

The DMS oxidation lasts only a few hours in the CLOUD chamber, while it can be for a few days in the atmosphere depending on the conditions. To gauge possible atmospheric evolution (a few days), we simulated OH-initiated DMS oxidation over a long timescale (∼1 week) at −10 °C, excluding walls and ventilation. As shown in Figure S18, terminal products MSA and H_2_SO_4_ accumulate in parallel over time, ultimately constituting the dominant reaction products. DMSO, MSIA, CH_3_SCHO, and CH_3_SOH are the precursors for MSA and H_2_SO_4_, reaching a steady state during the first day of simulation. HPMTF is a precursor for SO_2_, and after 1 week, both reach high concentrations and slowly approach steady state. Field measurements indicate high concentrations of HPMTF (up to 50 pptv in the marine atmosphere^32^) and its possible participation in particle formation. Accounting HPMTF chemistry in a global chemistry transport model^66^ results in a significant decrease in boundary layer levels of SO_2_ and H_2_SO_4_. Novak et al.^67^ demonstrate that the rapid loss of HPMTF to clouds terminates DMS oxidation to SO_2_. This is important at high temperatures. On the other hand, at low temperatures, HPMTF production is strongly reduced, and the main product becomes MSA via MSIA, so the fraction of oxidized DMS leading to SO_2_ is significantly lowered. The concentration of DMSO_2_, a terminal product of OH-initiated oxidation of DMS, remains low in the absence of heterogeneous reactions. These simulations suggest that MSA may be the dominant oxidation product of DMS oxidation for conditions found in the high-latitude marine atmosphere with low temperatures.

The agreement between the present chamber study, our model, and ambient observations over a wide temperature range is consistent with OH-initiated DMS oxidation governing the formation of gas-phase MSA and H_2_SO_4_ in the remote atmosphere. Our observation of many intermediate products closes gaps between the measured and modeled MSA. Specifically, MSIA and HPMTF are useful intermediates that serve as markers for OH addition and hydrogen abstraction channels, respectively. Therefore, measurement and proper calibration of these two species become important in future laboratory experiments and field observations for studying DMS. These findings improve our understanding of the atmospheric DMS oxidation process and its contribution to the natural sulfur cycle and potentially the formation of biogenic aerosol and thus CCN.

## Supporting Information Available

Additional description of quantitative measurement of DMS and its oxidation products; quantum chemical calculation method; detailed chemistry mechanism used in the box model; HO_2_ and OH estimation from the box model; heterogeneous wall reactions of DMS and O_3_ in the CLOUD chamber; temperature effect on the DMS oxidation process; and reactions of DMS oxidation chemistry (PDF)
