# Supplementary material for: High Gas-Phase Methanesulfonic Acid Production in the OH-Initiated Oxidation of Dimethyl Sulfide at Low Temperatures
Source: Environ Sci Technol. 2022 Sep 22;56(19):13931–44. doi: 10.1021/acs.est.2c05154 (PMC9535848; doi:10.1021/acs.est.2c05154)
Supplement: Supplementary file 1 — es2c05154_si_001.pdf [file es2c05154_si_001.pdf]

Supplementary Materials for

High Gas-Phase Methanesulfonic Acid Production in the OH-Initiated Oxidation of Dimethyl Sulfide at Low Temperature

Jiali Shen[1], Wiebke Scholz[2], Xu-Cheng He[1], Putian Zhou[1], Guillaume Marie[3], Mingyi Wang[4], Ruby Marten[5], Mihnea Surdu[5], Birte Rörup[1], Rima Baalbaki[1], Antonio Amorim[6], Farnoush Ataei[7], David M. Bell[5], Barbara Bertozzi[8], Zoé Brasseur[1], Lucía Caudillo[3], Dexian Chen[4], Biwu Chu[25,1], Lubna Dada[5], Jonathan Duplissy[1,9], Henning Finkenzeller[10], Manuel Granzin[3], Roberto Guida[11], Martin Heinritzi[3], Victoria Hofbauer[4], Siddharth Iyer[12], Deniz Kemppainen[1], Weimeng Kong[13], Jordan E. Krechmer[14], Andreas Kürten[3], Houssni Lamkaddam[5], Chuan Ping Lee[5], Brandon Lopez[4], Naser G. A. Mahfouz[15], Hanna E. Manninen[11], Dario Massabò[16], Roy L Mauldin[17,18], Bernhard Mentler[2], Tatjana Müller[3], Joschka Pfeifer[11], Maxim Philippov[19], Ana A. Piedehierro[20], Pontus Roldin[21], Siegfried Schobesberger[22], Mario Simon[3], Dominik Stolzenburg[1], Yee Jun Tham[23,1], António Tomé[24], Nsikanabasi Silas Umo[8], Dongyu Wang[5], Yonghong Wang[25,1], Stefan K. Weber[11,3], André Welti[20], Robin Wollesen de Jonge[21], Yusheng Wu[1], Marcel Zauner-Wieczorek[3], *Felix Zust*[2], Urs Baltensperger[5], Joachim Curtius[3], Richard C. Flagan[13], Armin Hansel[2], Ottmar Möhler[8], Tuukka Petäjä[1], Rainer Volkamer[10], Markku Kulmala[1,9,26,27,28], Katrianne Lehtipalo[1,20], Matti Rissanen[12], Jasper Kirkby[11,3], Imad El-Haddad[5], Federico Bianchi[1,*], Mikko Sipilä[1], Neil M. Donahue[4,17,29,30], and Douglas R. Worsnop[1,14]

*Corresponding author. Email: federico.bianchi@helsinki.fi

This PDF file includes:

Supplementary Text
Figs. S1 to S19
Tables S1 to S7
References (1 to 55)

S1. Quantitative measurement for DMS and its oxidation products

S1.1 Quantum chemical calculations

We used quantum chemical calculations to estimate the cluster formation enthalpies of sulfur-containing species and nitrate or bromide ions. The initial conformational sampling was performed using the Spartan'18 program [1]. The cluster geometry was then optimized using density functional theory methods at the ωB97X-D/aug-cc-pVTZ-PP level of theory [2, 3]. We take the bromide pseudo-potential definitions from the Environmental Molecular Sciences Laboratory (EMSL) basis set library [4, 5]. Calculations were carried out using the Gaussian 16 program [6]. An additional coupled-cluster single-point energy correction was carried out on the lowest energy geometry to calculate the final cluster formation enthalpy. The coupled-cluster calculation was performed at the DLPNO-CCSD(T)/def2-QZVPP level using the ORCA program version 4.0.0.2 [7, 8]. We present the calculated cluster formation enthalpies and fragmentation reaction enthalpies in Table S 3 and Table S 4.

S1.2. DMS measurement

A proton transfer reaction time-of-flight mass spectrometer (H_3O^+-CIMS) provided the DMS concentration. The instrument is based on the design of the proton transfer reaction time-of-flight mass spectrometer (PTR3) described in Breitenlechner et al., 2017 [9]. DMS concentrations were calibrated with a gas standard at specific conditions between experiments. The concentrations of DMS for the second experiment set were provided by the selective reagent ionization mass spectrometer (SRI-TOF-MS), described in detail by Canaval et al., 2019 [10]. As opposed to Canaval et al., 2019 [10], the SRI-TOF-MS was applied as a PTR-MS using H_3O^+ ions to ionize the sample gas at 2.5 mbar absolute pressure by proton transfer reactions described by Hansel et al., 1995 [11]. With a drift voltage of 540 V, the collision energy between the H_3O^+ ions and the sample gas molecules was 109 Td (1 Td = 10^{-17} V cm^2) during the experiments. This large collision energy creates an ion water cluster distribution that shifts towards H_3O^+, thereby resulting in an ionization efficiency independent of the humidity or temperature of the sample. During cleaning stages between experiments, we calibrated the instrument regularly with a standard gas mixture containing multiple volatile organic compounds to account for any possible drifts in transmission efficiency. Neither the SRI-TOF-MS nor the H_3O^+-CIMS was available for the third experiment set (CLOUD14). Therefore, we determined the DMS concentration with a numerical model based on the injection rates of the mass flow controller. Fig. S 1 plots the modeled and measured DMS concentration for experiment set 1 (A) and 3 (B) to show the agreement of the method in estimating the DMS

concentration. As shown in Fig. S 1, the difference between modeled DMS concentrations from measured values is small and does not exceed 40 % (A) and 75 % (B).

S1.3. H_2SO_4, MSA, and $CH_3S(O)_2OOH$ detected by NO_3^--CIMS

The concentrations of H_2SO_4, MSA, and $CH_3S(O)_2OOH$ were measured with a nitrate-ion-based chemical ionization mass spectrometer (NO_3^--CIMS; Tofwerk AG, Thun, Switzerland; Jokinen et al., 2012 [12]). The specially designed inlet for chemical ionization at the ambient pressure system and its calibration and quantification procedures are well described by previous studies [13,14]. We applied the same calibration coefficient $C_{H_2SO_4}$= 4.13 × 10^{10} cm^{-3} per normalized signal (cps cps^{-1}; cps, signifies counts per second) for experiments carried out at +10 and –10 °C since charging efficiency does not vary significantly in this temperature range. An inlet loss of 55% has already been included in the calibration factor. We were not able to calibrate other species during the campaign due to limited time and the lack of authentic standards or generation methods for species such as $CH_3S(O)_2OOH$. The cluster binding enthalpy calculated by quantum chemical methods is used to predict the sensitivity of analytes in an iodide-CIMS [15]. Unfortunately, it was not possible for us to establish such a correlation between sensitivities and binding enthalpies at the time for NO_3^--CIMS and the relevant compounds. Here, we predict the tentative critical enthalpy as the binding enthalpy of H_2SO_4 that is detected at the maximum sensitivity [16]. Thereby, we suggest a critical enthalpy not higher than 44.7 kcal mol^{-1}. MSA is detectable as deprotonated anion ($CH_3SO_3^-$, *m/z* = 94.98) and ion-adduct ($CH_3SO_3H{\cdot}NO_3^-$, *m/z* = 157.98), as in the case with H_2SO_4. The dissociation enthalpy of $CH_3SO_3H{\cdot}NO_3^-$ to CH_3SO_3H and NO_3^- is 34.4 kcal mol^{-1}, which is lower than the critical value but higher than the proton transfer pathway (28.5 kcal mol^{-1}). It suggests that dissociation of $CH_3SO_3H{\cdot}NO_3^-$ to CH_3SO_3H and NO_3^- may occur, but dissociation to $CH_3SO_3^-$ and HNO_3 is the dominant pathway. We can thus assume that MSA has a collision-limited charging efficiency like H_2SO_4, the same for uncertainty. $CH_3S(O)_2OOH{\cdot}NO_3^-$ has a low dissociation enthalpy, 27.5 kcal mol^{-1}. This suggests that the dissociation of $CH_3S(O)_2OOH{\cdot}NO_3^-$ to $CH_3S(O)_2OOH$ and NO_3^- does occur. Thus, we would expect that the sensitivity of $CH_3S(O)_2OOH$ is lower than the maximum sensitivity, and its concentrations represent lower limits when applying $C_{H_2SO_4}$.

S1.4. DMSO, $DMSO_2$, CH_3SCHO, and CH_3SOH measurements

We corrected all compounds with the calibration factor found by ionizing 1 ppbv of hexanone from a gas standard diluted in air. Hexanone is ionized at the kinetic limit in H_3O^+-CIMS and at ca. 80% of the kinetic limit in the NH_4^+-CIMS[18], which we corrected for accordingly. In H_3O^+ mode, this factor is the collision limit calibration factor due to the high proton affinity of hexanone compared

to the available $(H_2O)_nH_3O^+$ primary ion clusters. In the case of NH_4^+ chemical ionization, it is somewhat smaller at about 80 % of the collision limit at 20 °C and 10 % RH (dewpoint 3.6 °C), as shown by Zaytsev et al., 2019 [18]. At the humidity of experiment set 3 (25 % RH at 25 °C), its sensitivity is at 50 % of the collision limit in NH_4^+ mode and approaches the collision limit towards lower humidity.

By calibrating the instrument on a regular basis throughout the campaign, variable MS transmission efficiencies were accounted for with varying calibration factors. We used the collision limit calibration factor for all compounds so that the given concentrations are lower-limit estimates. This is especially true for compounds observed by only one of the ionization schemes, as is the case for CH_3SOH, which is only detected in H_3O^+ mode. CH_3SCHO does not show any significant humidity dependence in H_3O^+ mode, suggesting that its ionization is close to the collision limit, but we observed a humidity dependence in NH_4^+ mode. So CH_3SCHO concentrations obtained in the latter mode are lower limit estimates only. The qualitative behavior with temperature obtained from experiment 3 is nonetheless valid as we conducted the experiment (Set 3, 2294.09) at constant absolute humidity to reduce any such influence.

We used the collision limit calibration factor for all compounds to give lower-limit estimates for their concentrations. DMSO and $DMSO_2$, are likely ionized at the collision limit, as shown in Fig. S 2. Due to the high polarities of DMSO and $DMSO_2$, they are detected with multiple water clusters as $((H_2O)_mDMSO_{1,2})H^+$ and $((H_2O)_mDMSO_{1,2})NH_4^+$ clusters in H_3O^+- and NH_4^+-CIMS, respectively. We summed the different peaks after correcting for their different duty cycle transmissions. To test the binding energies of the different compounds, we varied the collision-induced-dissociation of the molecule-ion clusters by steadily increasing the voltage at the transition from the ionization region to the vacuum region of the mass spectrometer as done previously e.g., by Zaytsev et al., 2019[18]. The collision-induced dissociation scan (Fig. S 3), performed by varying the voltage at the transition from the ionization region to the low-pressure mass spectrometer, shows that DMSO and $DMSO_2$ are detected at maximum sensitivity in H_3O^+- and NH_4^+-CIMS. The used voltage setting during all experiments was 21V, shown as a vertical line. The collision-induced dissociation of the clusters sets in at significantly higher voltages than the used 21V and the dissociation of the $NH_3NH_4^+$ cluster, suggesting that the cluster ions involving DMSO and $DMSO_2$ are strongly bound and thus ionized at the kinetic limit. Furthermore, due to their high gas-phase basicity, breaking up the clusters with higher collision energies results in DMSO and $DMSO_2$ keeping the proton. No inlet line losses were applied because these compounds are volatile to semi-volatile. Therefore, the uncertainty of these compounds is mainly caused by the uncertainty of the

calibration factor. Table S 2 lists the detailed uncertainty for each species from different instruments.

S1.5. HPMTF, MSIA measurements

S1.5.1 HPMTF, MSIA measured in Bromide and Nitrate ionization

A bromide chemical ionization mass spectrometer coupled with a Multi-scheme chemical ionization inlet (Br^--MION-CIMS) and the gas phase measurement of bromide chemical ionization mass spectrometer equipped with a Filter Inlet for Gases and AEROsols (Br^--FIGAERO-CIMS) [19] were the primary instruments to detect MSIA and HPMTF. If neither instrument was available due to instrument malfunction or absence in some experiments, a NO_3^--CIMS was used to measure MSIA and HPMTF. The details of instrument setup and operation of the Br^--MION-CIMS and FIGAERO are described in Rissanen et al., 2019 [20] and Lopez-Hilfiker et al., 2014 [19], respectively. As shown in Fig. S 4, MSIA and HPMTF were detected in the NO_3^--CIMS, Br^--MION-CIMS, and gas-phase measurement of the Bromide-FIGAERO-CIMS (Br^--FIGAERO$_{(g)}$-CIMS) as analyte-reagent ion adducts. In this study, the NO_3^--CIMS was running and calibrated well for H_2SO_4 in all experiments. Since H_2SO_4 is detected at the collision limit in both NO_3^--CIMS and Br^--MION-CIMS [14, 16], normalized H_2SO_4 signals measured by Br^--MION-CIMS can be indirectly calibrated from the H_2SO_4 concentrations measured by the NO_3^--CIMS. We applied the same analysis to the Br^--FIGAERO$_{(g)}$-CIMS and NO_3^--CIMS. However, because of its higher detection limit due to substantial interference from neighboring peaks, the H_2SO_4 concentrations measured by the Br^--FIGAERO$_{(g)}$-CIMS are somewhat limited. Therefore, we derived the MSA calibration coefficient for both the Br^--MION-CIMS and Br^--FIGAERO$_{(g)}$-CIMS using the MSA concentration measured by the NO_3^--CIMS because MSA is detected at the maximum sensitivity in the NO_3^--CIMS and the same was assumed to be the case in the Br^--MION-CIMS. The MSA calibration coefficient for Br^--MION-CIMS, 2.0×10^{10} cm^{-3} cps cps^{-1} was derived from a linear fit ($[CH_3SO_3H] = 2.0 \times 10^{10}\ cm^{-3} \times (CH_3SO_3^- / 2 + CH_3SO_3 \cdot {}^{79}Br^-)/({}^{79}Br^- + H_2O \cdot {}^{79}Br^-) - 2.4 \times 10^5$) in Fig. S 5a. The linear fit ($[MSA]= 3.0 \times 10^{10}\ cm^{-3} \times (CH_3SO_3^- / 2 + CH_3SO_3 \cdot {}^{79}Br^-)/({}^{79}Br^- + H_2O \cdot {}^{79}Br^-) - 5.6 \times 10^6$) is depicted in Fig. S 5b. It shows a calibration coefficient of 3×10^{10} cm^{-3} cps cps^{-1} for MSA in the Br^--FIGAERO$_{(g)}$-CIMS.

MSIA and HPMTF are not calibrated directly due to the lack of authentic standards or generation methods and time limitations in this study. H_2SO_4 is believed to be detected at maximum sensitivity in the Br^--MION-CIMS [16], suggesting a critical enthalpy not higher than 33.7 kcal mol^{-1} (As the same instrument with the same tuning was deployed in Wang et al., 2021 [16], and this study, the critical enthalpy should be the same in both studies). On the other hand, the dissociation enthalpy of

$CH_3SOOH{\cdot}Br^-$ and $HOOCH_2SCHO{\cdot}Br^-$ are 25.5 and 27.1 kcal mol^{-1}, respectively; substantially lower than the tentative critical enthalpy, suggesting that they are not detected at the maximum sensitivity. Therefore, their concentrations are very likely underestimated when applying the same calibration coefficient as MSA. Nevertheless, we still report lower limits for MSIA and HPMTF concentrations. Table S6 summarizes the sensitivity of Br^--MION-CIMS and Iodide-CIMS to several species (such as H_2SO_4, N_2O_5) in the literature. As shown in Table S6, Cl_2 is detected at maximum sensitivity in the Iodide-CIMS, while its sensitivity is roughly ten times lower than the maximum sensitivity in Br^--MION-CIMS. In addition, the HNO_3 sensitivity is half the maximum sensitivity in the Iodide-CIMS, while it is five times less than the maximum sensitivity in the Br^--MION-CIMS. These findings indicate that the same analyte may show lower sensitivity in Br^--MION-CIMS compared to Iodide-CIMS. Veres et al., 2020 [21] performed a calibration experiment for HPMTF in an Iodide-CIMS and indicates that its sensitivity is similar to the most sensitive compounds (e.g., Cl_2, $ClNO_2$, N_2O_5). However, Ye et al., 2021 [22] estimated the sensitivity of HPMTF by performing voltage scanning between the skimmer and the second quadruple in the CIMS. By increasing the voltage gradient (ΔV), analyte reagent ion clusters will dissociate, and the degree of dissociation depends on the binding energy. The results [22] show that the sensitivity of HPMTF is around 100 times less than the maximum sensitivity of N_2O_5. Therefore, we assume that the sensitivity of HPMTF is likely much less than the maximum sensitivity in Br^--FIGAERO$_{(g)}$-CIMS. This likely is the primary reason for the large discrepancy between modeled and measured MSIA and HPMTF in Fig. 3.

The dissociation enthalpy of $CH_3SOOH{\cdot}NO_3^-$ and $HOOCH_2SCHO{\cdot}NO_3^-$ are 27.2 and 27.5 kcal mol^{-1}, respectively, lower than the critical value. We would expect their sensitivities are lower than the maximum sensitivity. $HNO_3{\cdot}NO_3^-$ has a larger dissociation enthalpy, 29.8 kcal mol^{-1}, than $CH_3SOOH{\cdot}NO_3^-$ and $HOOCH_2SCHO{\cdot}NO_3^-$. Thus, the sensitivity of CH_3SOOH and $HOOCH_2SCHO$ become even lower than previously thought since the $HNO_3{\cdot}NO_3^-$ cluster is stable, and HNO_3 has dominant concentrations over other species. This suggests that the detection sensitivity of MSIA and HPMTF in NO_3^--CIMS is even lower than in Br^--FIGAERO$_{(g)}$-CIMS, which wants additional corrections. As shown in Fig. S 6, the linear fits between Br^--FIGAERO$_{(g)}$-CIMS and NO_3^--CIMS for MSIA and HPMTF show that MSIA and HPMTF in NO_3^--CIMS should be corrected by a factor of 19 to 154, respectively. Therefore, we implement the corrected calibration coefficients of MSIA (7.8×10^{11} cm^{-3} cps cps^{-1}) and HPMTF (6.4×10^{12} cm^{-3} cps cps^{-1}) for NO_3^--CIMS in this study. Therefore, the concentrations of MSIA and HPMTF from Br^--FIGAERO$_{(g)}$-CIMS, Br^--MION-CIMS, and NO_3^--CIMS are lower-limit estimates.

S1.5.2 HPMTF, MSIA measured by H_3O^+-CIMS and NH_4^+-CIMS

As mentioned above, we assume both MSIA and HPMTF are not detected at maximum sensitivity by Br^--MION-CIMS, Br^--FIGAERO$_{(g)}$-CIMS, and NO_3^--CIMS. Thus, the given concentrations are lower-limit estimates with high uncertainties. Expect for Br^--MION-CIMS, Br^--FIGAERO$_{(g)}$-CIMS, and NO_3^--CIMS, both H_3O^+ and NH_4^+ -CIMS also measured MSIA and HPMTF. As shown in Fig. S 7, the ratio of measured MSIA in the H_3O^+ and NH_4^+ -CIMS is humidity independent, indicating that MSIA is likely detected at the collision limit in both modes. Having humidity dependence or not is a strong indicator to determine whether the species is detected at the collision limit because detection at the collision limit in these ionization schemes requires the species to perform ligand-switching with all of the water clusters of primary ions. It is therefore highly unlikely that any humidity dependence in both modes would be the same due to the different binding energies of water molecules to H_3O^+ or NH_4^+. The comparison of Br^--FIGAERO$_{(g)}$-CIMS with NH_4^+ and H_3O^+-CIMS in Fig. S 7 suggests either a stronger fragmentation at dryer conditions or a weaker ionization efficiency of Br^- compared to (H_2O) Br^-. For Br^-, the calibration of MSIA with the MSA calibration factor gives slightly lower values, but the ratio to the other two ionization modes is again independent of the humidity and thus suggests that the ionization occurs as well at the kinetic limit, but the signal might be underlying some fragmentation or other unaccounted losses in the instrument or inlet. Therefore, as shown in Fig. S 7, the difference between the absolute MSIA concentrations between H_3O^+-CIMS and Br^--FIGAERO$_{(g)}$-CIMS is ~a factor of 5, which can be explained the overestimated sensitivity of MSIA in Br^--FIGAERO$_{(g)}$-CIMS. In the case of HPMTF, the Br^--FIGAERO$_{(g)}$-CIMS shows lower values than the other ion two modes under very dry conditions, while the applied MSA calibration factor captures the higher-humidity data very well. NH_4^+ and H_3O^+ mode is both ionizing HPMTF at or close to the kinetic limit, as Fig. S 8 suggests, the HPMTF data from the very dry and cold experiments from the H_3O^+ ionization scheme is however very uncertain because the HPMTF signal is divided onto many different masses in the H_3O^+ CIMS (as can be seen in Fig. S 8) so that it can easily be affected by neighboring peaks, especially when its concentration is very low.

MSIA is a wall-loss species, like MSA and H_2SO_4, and is easy to be destroyed on a metal surface, as shown in Fig. S 9. We apply a total inlet and instrumental loss correction factor of 5.25 for compounds destroyed on metal surfaces because the PTR3 has metal (from the tripol) directly at the ionization zone, where the sample gas flow is also deviated by about 40° from its original flow direction and thus somewhat turbulent. While we apply the maximum loss correction factor for MSIA, we only apply a correction of 66% of the maximum factor for HPMTF since we determined

the loss of HPMTF in a metal tube to be only 66%, while MSIA is fully lost (see the details in Fig. S 9). The uncertainties of MSIA and HPMTF concentrations from H_3O^+-CIMS come from calibration and loss correction from the inlet and instrument, which are around 28% and 54%, respectively. The higher uncertainty for HPMTF is caused by the high background correction. Compared to the H_3O^+-CIMS, the NH_4^+-CIMS has a higher uncertainty for MSIA and HPMTF due to the larger uncertainty of the collision limit.

S2. Chemistry mechanism for Box model

In this study, the basic gas-phase chemistry mechanism was based on MCMv3.3.1 [23] and Hoffmann et al., 2016 [24]. Additionally, new reactions such as pathway 2b, the reaction of CH_3SOH with O_3; and pathway 1b in which methylthiomethylperoxy radical (CH_3SCH_2OO, MSP) undergoes a fast isomerization (Fig. 1); and the updated rate constants were implemented from previous studies [21, 25, 26, 27]. In summary, the DMS oxidation chemistry mechanism includes 75 species and 156 reactions listed in Table S7.

As shown in Fig. 1, the first primary attack of OH radicals towards DMS proceeds via (1) a hydrogen abstraction channel or (2) a reversible OH addition channel. The branching ratio of the addition/abstraction channel decreases with increased temperature. Besides, we include (3) heterogeneous reactions of DMS including O_3 on the wall with semi-empirical reaction rate coefficients. Heterogeneous reactions are the predominant source of $DMSO_2$. Their contribution to oxidation products (except $DMSO_2$) is negligible compared to gaseous reactions.

In the hydrogen abstraction channel (1), after the H abstraction and consecutive O_2 addition, MSP is formed. It reacts further with hydroperoxyl radicals (HO_2), peroxy radicals (RO_2), or NO_x [28]. The reaction with NO_x forming methyl thiomethoxy radical (CH_3SCH_2O) dominates the removal of MSP [23, 29]. In the previous studies [28], the major primary oxidation products from MSP are CH_3SCHO and CH_3SCH_2O. Then the decomposition of CH_3SCH_2O and the reaction of CH_3SCHO with OH lead to methyl thiyl radicals (CH_3S). CH_3S is a critical compound in the following reaction steps to form MSA and H_2SO_4. In the absence of O_3 and NO_x, O_2 represents the possible reaction partner for CH_3S producing methyl thioperoxyl radical (CH_3SO_2), which undergoes an isomerization step forming methyl sulfonyl radicals ($CH_3S(O)_2$). The O-atoms in $CH_3S(O)_2$ are directly bound to the sulfur atom by a double bond. Further reactions of $CH_3S(O)_2$ have strong temperature dependence, which either decomposes to SO_2 or reacts with O_3 forming CH_3SO_3. Interestingly, CH_3SO_3 also involves a thermal decomposition forming SO_3 or reacts with HO_2 forming MSA. With the presence of O_2, $CH_3S(O)_2$ reacts to methyl sulfonyl peroxyl radicals ($CH_3S(O)_2O_2$), forming methane sulfonic peroxide ($CH_3S(O)_2OOH$) with HO_2.

The application of a high-sensitivity detection instrument – chemical ionization mass spectrometers – improves our understanding of DMS chemistry kinetics. For example, a new intermediate product hydroperoxymethyl thioformate ($HOOCH_2SCHO$, HPMTF), was recently observed in the marine atmosphere [21] and then simulated in laboratories [22, 26]. These observations support the mechanism (pathway 1b) proposed by Wu et al., 2015 [25]. This mechanism starts from a fast intramolecular hydrogen shift in MSP. Afterward, the new peroxy radical $O_2CH_2SCH_2OOH$ undergoes another isomerization and decomposes to a stable intermediate product HPMTF. HPMTF further reacts with OH or O_3 to form SO_2. The isomerization pathway (1b) changes the fate of MSP by competing with biomolecular chemistry, especially at high temperatures. For example, the MSP isomerization is predominant ($\geq$ 95%) at +25 °C. This suggests that the hydrogen abstraction channel plays an important role at high temperatures. The isomerization reaction rate coefficient of MSP has been determined to be $0.23 \pm 0.12\ s^{-1}$ at 295 ± 2 K [26] through flow tube experiments, $2.2 \times 10^{11} \times \exp(-9.8 \times 10^3 / T) \times \exp(1.0 \times 10^8 / T^3)$ from multi-conformer transition state theory (MC-TST) calculation [21], $0.09\ s^{-1}$ (0.03–$0.3\ s^{-1}$) from a chamber study [22], and $0.1 \pm 0.05\ s^{-1}$ from another flow reactor study [30]. The isomerization reaction rates predicated by quantum chemical methods account for the temperature dependency which is critical in this study. At 295 K, the theoretical calculation gives a rate of $0.04\ s^{-1}$, which is around 2 to 5 times smaller than the experimental values. In this study, we apply $2.2\times10^{11} \times \exp(-9.8 \times 10^3 / T) \times \exp(1.0 \times 10^8 / T^3) \times f$ ($f = 2$–5) for the isomerization reaction rate coefficient of MSP in the box model. Additionally, the overall error is presented by grey shade in Fig. 3 by applying $f = 1$ to 5.

In the OH addition channel, the formation of OH-DMS adduct ($CH_3S(OH)CH_3$) is O_2-dependent and is well understood. DMSO and $DMSO_2$ are formed from $CH_3S(O_2)(OH)CH_3$ via $CH_3S(OH)CH_3$ reaction with O_2. The production of DMSO and $DMSO_2$ is sensitive to NO_x levels due to the reaction of $CH_3S(O_2)(OH)CH_3$ with NO_x. Previous studies [31] also suggest that the $CH_3S(O)(OH)CH_3$ adduct from DMSO and OH yields $DMSO_2$ by reacting with O_2. In this study, we propose a small reaction rate coefficient for $DMSO_2$ that is produced from DMSO oxidation with OH, which is about 90 times lower than that of forming methanesulfinic acid ($CH_3S(O)OH$, MSIA). The further oxidation of $DMSO_2$ is low (with OH, O_3, or other oxidants), indicating its long lifetime. MSIA is the primary oxidation product formed from DMSO that reacts with OH in the absence and presence of NO_x [32]. The previous studies [33, 34] suggest that the reaction of MSIA and OH in the gas phase forms SO_2 only. However, the abstraction of an acidic H-atom by OH radicals is typically slow because acidity implies a deficiency in electron density [35]. It is likely that OH adds to the S-atom in MSIA, producing an intermediate product. It may decompose rapidly to form

sulfurous acid (H_2SO_3) and CH_3 [36] or react with O_2 producing MSA. But this pathway and its reaction rate coefficients are not fully studied. Therefore, we treat the reaction of MSIA with OH forming $CH_3S(O)_2$ in our box model; it either decomposes to SO_2 or reacts with O_3 yielding MSA [31, 37]. Consequently, both the addition and abstraction pathways contribute to MSA production via the reaction of MSIA with OH. The formation of DMSO and MSIA is the dominant pathway in the OH addition channel. Also, the DMSO will react with OH producing aqueous MSIA in the aqueous phase, which can further form aqueous MSA via reacting with OH [24, 38]. However, both the gaseous MSIA and MSA show wall loss lifetime in the CLOUD chamber, so we would suggest that they are unlikely to be produced from the wall. But we cannot completely rule out the aqueous-phase reactions of DMSO with OH and MSIA with OH. We also include pathway 2b for the OH addition channel, in which the oxidation of methane sulfenic acid (CH_3SOH) by OH or O_3 forms SO_2 or CH_3SO. CH_3SOH is an intermediate product formed from the decomposition of OH-DMS adduct ($CH_3S(OH)CH_3$) [31]. In this study, the reaction with O_3 is the dominant sink for CH_3SOH because the O_3 concentration is much higher than that of OH (a factor of thousands), but the difference between these two reaction rates is small and does not exceed a factor of 25, while the reaction with OH is faster.

As discussed above, HO_2 influences the DMS oxidation pathway via several reactions; and it is critical at low temperatures when the isomerization reaction slows down. In this case, the increased HO_2 can reduce HPMTF formation by enhancing bimolecular reactions. Besides, HO_2 enhances MSA formation in the final reaction step of CH_3SO_3 with HO_2 via competing with thermal decomposition. These determine the importance of HO_2 simulation, which will be discussed in the next section.

The adopted mechanism here introduces an additional way via DMSO oxidation to MSIA, which increases MSA production. MSA is produced almost exclusively from the reaction of CH_3SO_3 with HO_2 through abstraction pathway 1a and addition pathway 2a. The fraction of MSA can be presented by the production rates of $CH_3S(O)_2$ formation. $CH_3S(O)_2$ formed from 1) CH_3SO_2 or CH_3SO in the abstraction pathway 1a, MSIA produced from the addition pathway further reacts with 2) OH and NO_3 radicals, and 3) $DMSO_2$ reacts with OH. At –10 °C, MSIA reacts with OH is the dominant pathway to form $CH_3S(O)_2$. Although $DMSO_2$ is high, the reaction rate coefficient of $DMSO_2$ with OH is low, 1×10^{-14} molecule^{-1} cm^3 s^{-1}, leading to a production rate of $\sim 1 \times 10^3$ s^{-1} cm^{-3}. While it still needs to undergo two more steps to form $CH_3S(O)_2$, thus, the formation of $CH_3S(O)_2$ from $DMSO_2$ is tiny. The production rate for $CH_3S(O)_2$ formed from the abstraction pathway is around 3×10^3 s^{-1} cm^{-3}. While the production rate for MSIA and OH forming $CH_3S(O)_2$ is $\sim 1\times 10^5$ s^{-1}. Therefore, around 90% of the MSA is formed from addition pathway 2a at –10 °C.

In the model, H_2SO_4 is formed from SO_3 and the oxidation of SO_2 with OH. Our model suggests that the dominant source of H_2SO_4 is SO_3, which is produced by the thermal decomposition of CH_3SO_3 and the reaction of MSIA with OH. There has been no report of any direct experimental evidence for the formation of SO_3 in DMS oxidation. However, field observation notes that the measured H_2SO_4 could not be explained by oxidation of SO_2 with OH only [39]. In this study, we do not have experimental evidence to prove the occurrence of reactions, but if occurring, it would be important. SO_2 is formed mainly through three pathways: 1) pathway 1a, decomposition of $CH_3S(O)_2$; 2) pathway 2b, the reaction of CH_3SOH with O_3; 3) pathway 1b, the further oxidation of HPMTF.

S3. HO_2 and OH in the box model

As mentioned before, HO_2 and OH are important, which were not measured but simulated in the box model based on the chamber parameters of temperature, relative humidity, DMS, O_3, CO, NO_x concentration, and O_3 photolysis rate. HO_2 is produced from the reactions of OH with O_3, CO, H_2, NO_3, and loss by reacting with NO_2, O_3, and DMS oxidation products. Considering the reaction rate constants and the conditions of our experiments, OH, O_3 and CO are the primary parameters that determine HO_2 concentration and its uncertainty. In this study, we applied the direct measurements of O_3 and CO to our box model, which increases the accuracy of HO_2 simulation. The employed Br^--MION-CIMS and Br^--FIGAERO$_{(g)}$-CIMS in different experiment sets can measure HO_2. However, HO_2 is not calibrated, and its detection efficiency strongly depends on relative humidity therefore, direct HO_2 measurement is not available.

Although OH measurement was not available, the estimated concentration in the box model is well simulated. In the box model, OH is believed to be a wall loss species according to the mobility calculation from He et al., 2021 [40]. Besides, the most critical parameter for estimated OH concentration is the photolysis rate from UV lights; we calibrated them between different campaigns via the calibration experiment of H_2SO_4 formed from the oxidation of SO_2 by OH. We always used the same amount of SO_2, O_3, H_2O, CO, and lights and employed the same NO_3^--CIMS measuring H_2SO_4. The OH production (Q_{OH}) can be calculated by the following equations (Eq 9-11) and agree with each other within certain uncertainty, as shown in the previous campaign. The OH production is used to estimate the photolysis rate of O_3 that is applied in the box model. Therefore, we would like to assume the OH concentrations are well evaluated in the box model.

$$\frac{d[H_2SO_4]}{dt} = k_{SO_2+OH}[SO_2] \times [OH] - [H_2SO_4] \times k_{wall-loss} \quad \text{Eq9}$$

$$\frac{d[OH]}{dt} = Q_{OH} - k_{SO_2+OH}[SO_2] \times [OH] - k_{O_3+OH}[O_3] \times [OH] - k_{CO+OH}[CO] \times [OH] \quad \text{Eq10}$$

$$Q_{OH} = \left(k_{SO_2+OH}[SO_2] + k_{O_3+OH}[O_3] + k_{CO+OH}[CO]\right) \times \frac{[H_2SO_4] \times k_{wall-loss}}{k_{SO_2+OH}[SO_2]} \quad \text{Eq11}$$

S4. Heterogeneous wall reactions of DMS and O_3

The negligible decay of DMSO and $DMSO_2$ during the dark stage in Fig. 2 strongly suggests a dark source producing DMSO and $DMSO_2$, which requires only DMS, O_3, and H_2O. This occurs on walls [41, 42]. Fig. S 11 shows the wall reactions of DMS and O_3 in the first 155 minutes of an experiment starting with clean conditions and turn-off UV lights (before the vertical dotted line). In this case, DMSO appears when DMS is injected (with a nearly constant O_3 mixing ratio at ~ 34 ppbv) and increases rapidly from 8×10^6 to 2.3×10^8 cm^{-3}. $DMSO_2$ appears 15 minutes later than DMSO and increases from 2.3×10^7 to 1.6×10^9 cm^{-3}. The different overall shapes versus time (linear and quadratic) and the time delay indicate that $DMSO_2$ is a second-generation product from the first-generation product DMSO. We plot time sequences of DMSO and $DMSO_2$ after DMS injection on a linear scale in Fig. S 11 to show the appearance time differences. The appearance times of DMSO and $DMSO_2$ are distinctly longer than the oxidation products formed in the gas-phase reactions in Fig. 2. Based on these observations, we invoke two wall reactions and two semi-empirical rate coefficients (see next section for details of the calculation) for the heterogeneous formation of DMSO and $DMSO_2$:

$$\mathrm{DMS} + \mathrm{O_3} \xrightarrow{k_{\mathrm{DMS+O_{3wall}}}} \mathrm{DMSO} \quad \text{Eq 1}$$

$$\mathrm{DMSO} + \mathrm{O_3} \xrightarrow{k_{\mathrm{DMSO+O_{3wall}}}} \mathrm{DMSO_2} \quad \text{Eq 2}$$

where $k_{\mathrm{DMS+O_{3wall}}} = 3.4 \times 10^{-18}$ molec^{-1} cm^3 s^{-1} and $k_{\mathrm{DMSO+O_{3wall}}} = 3 \times 10^{-15}$ molec^{-1} cm^3 s^{-1}, at + 25 °C and 24 % RH in the CLOUD chamber. This is likely aqueous-phase oxidation of DMS [24, 42], in this case with wall-adsorbed water. A similar process may well occur in aerosol water and droplets in the marine atmosphere, and the implication of the reaction rate constants needs to be modified depending on the conditions. However, the reaction rate constant for the DMSO reaction is about 103 times higher than for the DMS reaction with O_3, which is inconsistent with the results in Gershenzon et al., 2001[43]. This opposite behavior might be explained by other chemical reactions besides ozonolysis or a catalytic effect of stainless steel on the chemical reactions as has been observed previously for hydroperoxides [44]. This is, however, speculation as we have no clear evidence for such a process. Except for O_3, halogen species like Br, Cl, and I_2 and their oxidation species can also react with DMS. However, none of them were introduced in the chamber and neither has been detected by our mass spectrometers. Therefore, these two semi-empirical reaction rate constants are surrogates for the possible reactions noted above and should only be used for

accounting for DMS and DMSO wall reactions in this study specifically and should not be used for atmospheric simulations. In this study, we are not able to investigate the temperature or RH dependence for these two semi-empirical parameters with only one experiment. While the constant $DMSO_2$ concentration in the temperature ramping experiment 2 (Fig. S 14) shows that the heterogeneous reactions have a weak temperature dependence over that range. Aside from DMSO and $DMSO_2$, we see no evidence that wall reactions have influenced the other oxidation products in the dark experiment. DMSO and $DMSO_2$ are the only two oxidation products showing production in the dark, indicating there is no direct formation of other oxidation products from wall reactions with DMS and O_3 only. Fig. 2 also shows that all the other products exhibit either rapid wall loss or in the case of CH_3SCHO, ventilation loss only. The CLOUD walls present an effective condensation sink of 2×10^{-3} s^{-1}, which is 5–7 times higher than those reported for the pristine Marine Boundary Layer [45] but is within the range typically observed in coastal areas [46, 47]. With lights on (after the dotted line), the OH-initiated DMS gas-phase oxidation commenced, leading to higher production rates for both DMSO and $DMSO_2$. In this case, subtracting the wall reactions, the production from OH-initiated DMS oxidation and the loss to OH radicals control the appearance time of DMSO and $DMSO_2$. We find $k_{\mathrm{DMS+OH \to DMSO_{gas}}} = 1.2 \times 10^{-12}$ molec^{-1} cm^3 s^{-1} and $k_{\mathrm{DMSO+OH \to DMSO_{2gas}}} = 1 \times 10^{-12}$ molec^{-1} cm^3 s^{-1} by fitting the time series of DMSO and $DMSO_2$. These two values are not included in our box model. The modeled time evolution of DMSO and $DMSO_2$ (solid lines in Fig. S 11B) includes chamber ventilation loss, the semi-empirical coefficients for wall reactions, OH-initiated DMS oxidation, and further oxidation reactions with OH. With the presence of OH, the further oxidation of DMSO in the gas phase is processed rapidly to form MSIA [33]. But the oxidation of $DMSO_2$ with OH is complex and slow [24]. Therefore, the subtle increase in the DMSO time trace when UV lights were turned on in Fig. S 11B is a consequence of substantially increased production from OH + DMS in the gas phase and substantial loss, again via OH (~ 5×10^{-4} s^{-1}). The modeled DMSO and $DMSO_2$ concentrations closely follow the measured values, demonstrating that the fitted semi-empirical wall reaction coefficients work well.

S4.1. Calculation of the semi-empirical reaction rate coefficient

$k_{\mathrm{DMS+O_{3wall}}}$ and $k_{\mathrm{DMSO+O_{3wall}}}$ were determined by fitting DMSO and $DMSO_2$ time series in the dark experiment using the following equations:

$$\frac{d[\mathrm{DMSO}]}{dt} = \frac{d[\mathrm{DMSO_w}]}{dt} = k_{\mathrm{DMS+O_{3wall}}} \times [\mathrm{DMS}] \times [\mathrm{O_3}] - k_{\mathrm{loss}} \times [\mathrm{DMSO_w}] \qquad \text{Eq 3}$$

$$\frac{d[\mathrm{DMSO_2}]}{dt} = \frac{d[\mathrm{DMSO_{2,w}}]}{dt} = k_{\mathrm{DMSO+O_{3}}_{\mathrm{wall}}} \times [\mathrm{DMSO_w}] \times [\mathrm{O_3}] - k_{\mathrm{loss}} \times [\mathrm{DMSO_{2,w}}] \qquad \text{Eq 4}$$

where $DMSO_w$ and $DMSO_{2,w}$ refer to gaseous DMSO and $DMSO_2$ formed from heterogeneous wall reactions. To determine these two rate coefficients, it is assumed that the adsorbed DMS and O_3 on the walls are proportional to gaseous DMS and O_3, respectively. In this case, we simulated the time series of DMSO and $DMSO_2$ by solving the differential equations Eq 3 and Eq 4 with an initial random guess for the parameters ($k_{\mathrm{DMS+O_{3}}_{\mathrm{wall}}}$, $k_{\mathrm{DMSO+O_{3}}_{\mathrm{wall}}}$) using the function ODEINT in Python. After that, we evaluated the simulated results by applying the least-squares - the sum squared difference between the simulated $f(x_i;p)$ and measured data points ξ_i.

$$S = \sum_i \left(\xi_i - f(x_i;p)\right)^2 \qquad \text{Eq 5}$$

Both ξ_i and $f(x_i;p)$ are normalized to the measured data. Simulated annealing [48], a probabilistic technique for approximating the global optimization in a large search space for an optimization problem, is applied to find the best solution (here referring to the parameters). A basic iteration includes updating the parameter p' randomly in the range of (0,1) and calculating its corresponding S'. Then the system considers the neighboring S and decides between moving the system to S'or staying in S.

$$l = S' - S \qquad \text{Eq 6}$$

We apply a loss l to evaluate the parameters: when $l < 0$, S and p are replaced by S' and p', when $l > 0$, S and p will be replaced within certain possibilities (Metropolis probability).

The same analysis was applied to determine $k_{\mathrm{DMS+OH\rightarrow DMSO}_{\mathrm{gas}}}$and $k_{\mathrm{DMSO+OH\rightarrow DMSO_{2}}_{\mathrm{gas}}}$ in the experiment which includes wall and gas-phase reactions. Here, the equations change to:

$$\frac{d[\mathrm{DMSO}]}{dt} = \frac{d[\mathrm{DMSO_w}]}{dt} + \frac{d[\mathrm{DMSO_g}]}{dt}$$
$$= k_{\mathrm{DMS+O_{3}}_{\mathrm{wall}}} \times [\mathrm{DMS}] \times [\mathrm{O_3}] + k_{\mathrm{DMS+OH\rightarrow DMSO}_{\mathrm{gas}}} \times [\mathrm{DMS}] \times [\mathrm{OH}]$$
$$- k_{\mathrm{DMSO+OH}_{\mathrm{gas}}} \times [\mathrm{OH}] \times [\mathrm{DMSO}] - k_{\mathrm{loss}} \times [\mathrm{DMSO}] \qquad \text{Eq 7}$$

$$\frac{d[\mathrm{DMSO_2}]}{dt} = \frac{d[\mathrm{DMSO_{2,w}}]}{dt} + \frac{d[\mathrm{DMSO_{2,g}}]}{dt}$$
$$= k_{\mathrm{DMSO+O_{3}}_{\mathrm{wall}}} \times [\mathrm{DMSO_w}] \times [\mathrm{O_3}] + k_{\mathrm{DMSO+OH\rightarrow DMSO_{2}}_{\mathrm{gas}}} \times [\mathrm{DMSO}] \times [\mathrm{OH}]$$
$$- k_{\mathrm{DMSO_2+OH}_{\mathrm{gas}}} \times [\mathrm{OH}] \times [\mathrm{DMSO_2}] - k_{\mathrm{loss}} \times [\mathrm{DMSO_2}] \qquad \text{Eq 8}$$

where $DMSO_g$ and $DMSO_{2,g}$ refer to gaseous DMSO and $DMSO_2$ formed from gas-phase and heterogeneous wall reactions. $k_{DMSO+OH_{gas}}$ = 8.9 ×10^{-11} cm^3 s^{-1}, $k_{DMSO_2+OH_{gas}}$ = 4.4 ×10^{-14} cm^3 s^{-1}, taken from MCMv3.3.1, and k_{loss} is ventilation loss, 1.6 ×10^{-4} s^{-1} in experiment set 3.

S5. Temperature effect on DMS oxidation products

MSA has a stronger temperature dependence than H_2SO_4. As shown in Fig. S 14, when the temperature decreases from +25 to +10 °C (Exp 2), the MSA concentration increases by an order of magnitude from 8 × 10^5 to 1.4 × 10^7 cm^{-3}, while the H_2SO_4 concentration increases only by a factor of 1.5 compared to its initial concentration. The formation of H_2SO_4 from SO_2 reacting with OH radical depends on temperature; however, this effect is small compared to the exponential temperature function of thermal decomposition. At the same time, various intermediates increase by various factors: MSIA 60 %, CH_3SCHO a factor of 1.9, CH_3SOH 87 %, $CH_3S(O)_2OOH$ a factor of 6.8, and DMSO a factor of 1.3. This is consistent with the mechanism in Fig. 1. As temperature decreases, the DMS oxidation initiated by OH favors OH addition rather than hydrogen abstraction, leading to high yields of DMSO, MSIA, and CH_3SOH. Therefore, the oxidation product $CH_3S(O)_2$, produced by MSIA reaction with OH, increased by decreasing temperature. The increase of $CH_3S(O_2)OH$ with temperature is direct evidence to support the enhancement of $CH_3S(O)_2$. Further, thermal decomposition rates of $CH_3S(O)_2$ and CH_3SO_3 to SO_2 slow down at the decreased temperature. On the other hand, the degradation of $CH_3S(O)_2$ and CH_3SO_3 increases the yields of MSA. Because the isomerization rate constants of MSP decreased by 66 % from 25 to 10 °C, as shown in Fig. S 14, the modeled HPMTF decreases slightly with temperature in Exp 2. This is inconsistent with our observation of essentially constant HPMTF concentrations, which could connect to the low detection efficiency of HPMTF in the NO_3^--CIMS. In Exp 1, however, the HPMTF concentration (detected by Br^--FIGAERO$_{(g)}$-CIMS) dropped intensively with the decreased temperature in Exp 1. The constant $DMSO_2$ concentration in Exp 2 shows that the temperature dependence of the wall reactions is low. Overall, decreasing temperature (from +25 to –10 °C) enhances the yields of CH_3SCHO, $CH_3S(O)_2OOH$, CH_3SOH, and MSA. As shown in Fig. S14, the simulated time series for the oxidation products show similar trends in different mechanisms. However, the absolute concentrations vary from species to species. For example, the simulated H_2SO_4 concentrations from this study are closer to the experimental results than the other two mechanisms. The simulated MSA concentrations from Hoffman et al., 2016 show a better simulation result for Exp 1, while the results from MCMv3.3.1 show a better simulation result for Exp 2. The limited time for the temperature ramping experiment and uncertainties for the

temperature function of reaction rate constants likely explain the discrepancy between the simulated and experimental results for the oxidation products.

Figures and Tables

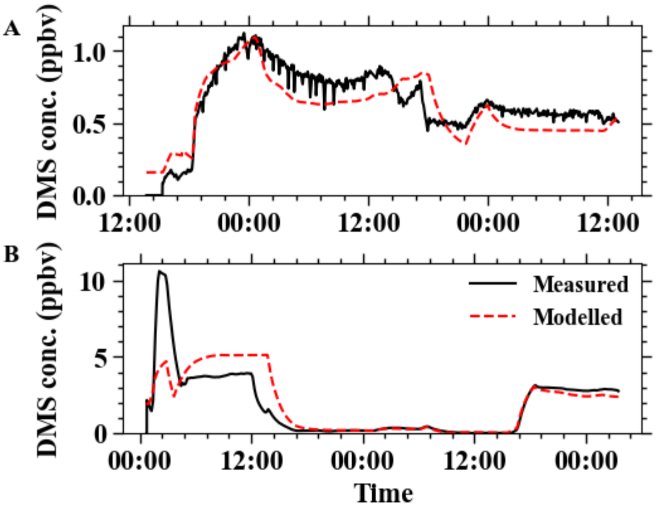

Fig. S 1. Measured and modeled DMS concentration in (A) experiment set 1 and (B) experiment set 2. The measured DMS concentration is detected by SRI-TOF-MS or H_3O^+-CIMS (black solid line). The modeled DMS concentration is calculated considering the injection rate of DMS and the ventilation loss. The modeled values agree with the trend of measured values, and the difference is within 40 % (A) and 75 % (B).

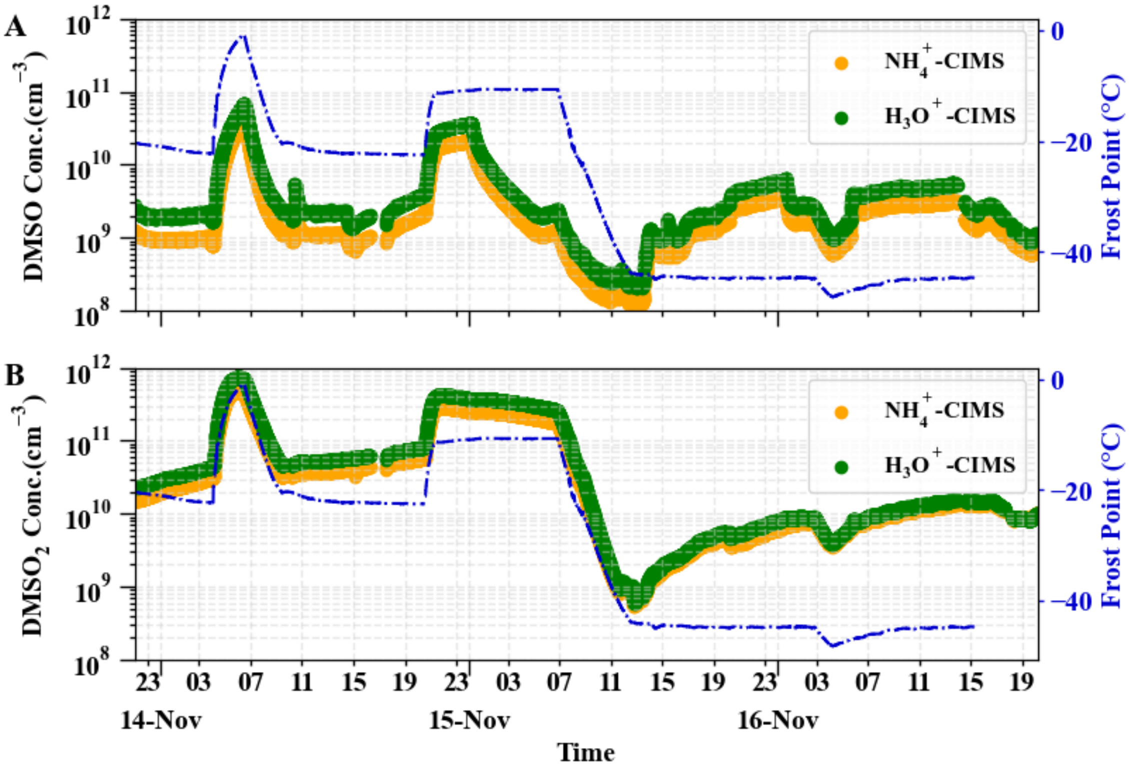

Fig. S 2. The collision limit calibrated A) DMSO and B) $DMSO_2$ from H_3O^+-CIMS, and NH_4^+-CIMS. The humidity independent ratio between the two ionization methods suggests a collision limited ionization efficiency for both compounds using both ionization methods. The uncertainty of the calibration factor can explain the small difference between the determined concentrations.

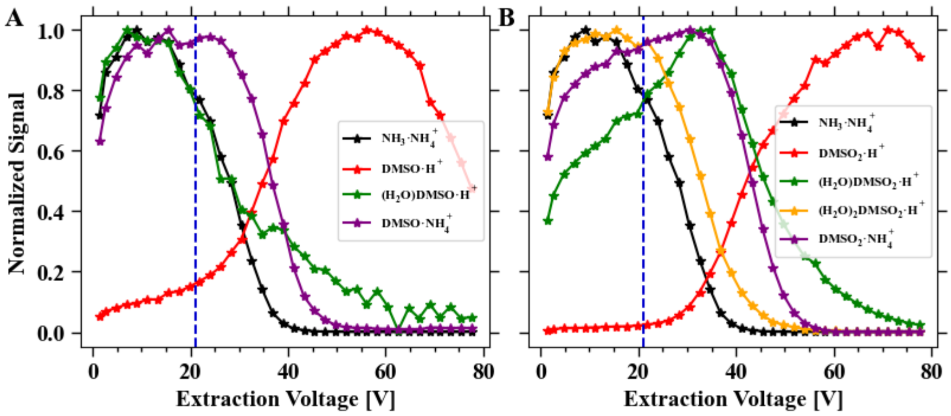

Fig. S 3. The signals for DMSO (A) and $DMSO_2$ (B) cluster ions observed, normalized to their respective maximum signals vs. the voltage difference in the transition region from the ion-molecule reaction region to the low-pressure mass spectrometer in H_3O^+-CIMS and NH_4^+-CIMS. The used voltage setting during all experiments was 21V, shown as a vertical line.

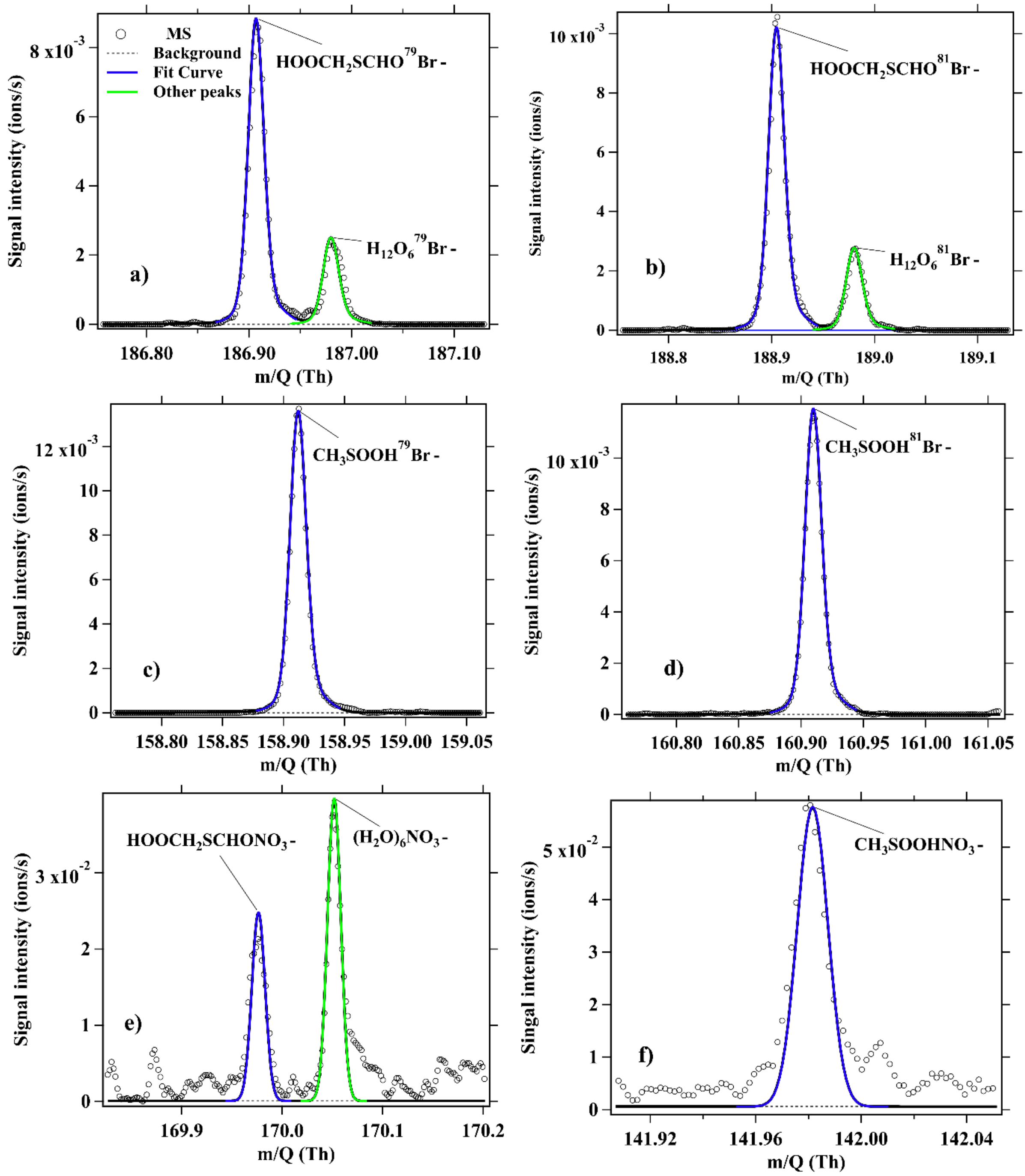

Fig. S 4. High-resolution peak fittings for HPMTF and MSIA measured by Br^--MION-CIMS and NO_3^--CIMS. a) and b) The peaks of HPMTF that cluster with $^{79}Br^-$ and its isotope ($^{81}Br^-$). c) and d) The peaks of MSIA that cluster with $^{79}Br^-$ and its isotope ($^{81}Br^-$). e) and f) The peaks of HPMTF and MSIA that cluster with NO_3^-. The blue-solid lines are HPMTF and MSIA peaks. The green-solid lines are other peaks.

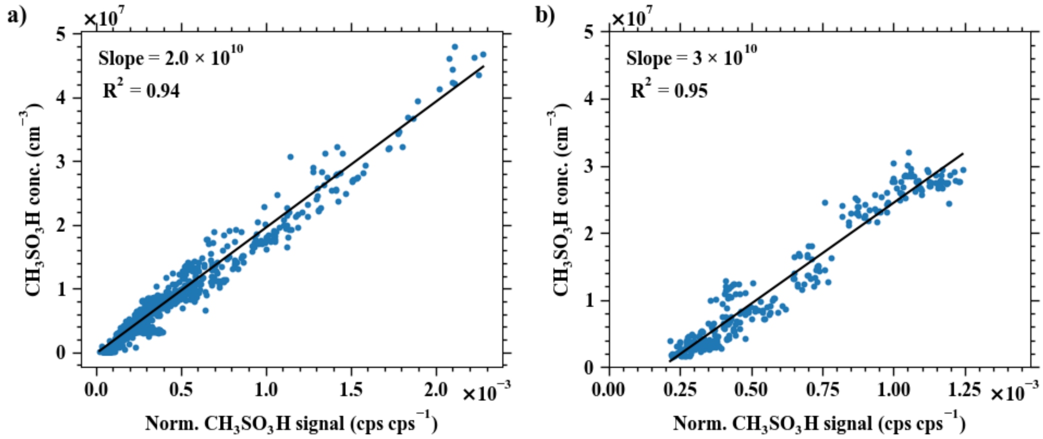

Fig. S 5. Calibration of MSA measured by Br⁻-MION-CIMS and Br⁻-FIGAERO$_{(g)}$-CIMS by comparing with NO_3^--CIMS data. a) Absolute MSA concentrations (cm^{-3}) measured by NO_3^--CIMS vs. the normalized signals (cps cps^{-1}) measured by Br⁻-MION-CIMS. b) The absolute MSA concentrations (cm^{-3}) measured by NO_3^--CIMS vs. the normalized signals (cps cps^{-1}) measured by Br⁻-FIGAERO$_{(g)}$-CIMS. Black solid lines are linear fittings.

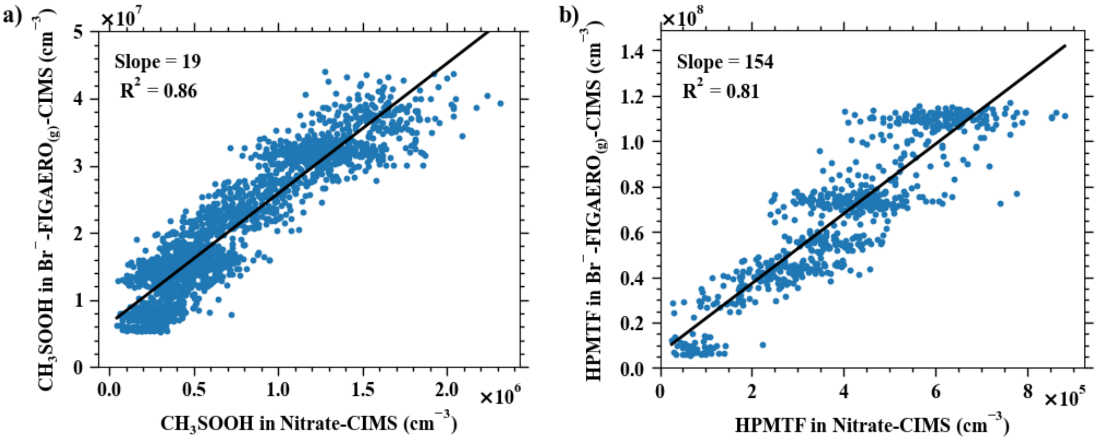

Fig. S 6. Intercomparison between Br⁻-FIGAERO$_{(g)}$-CIMS and NO_3^--CIMS for (a) MSIA and (b) HPMTF. The black solid lines are linear fittings.

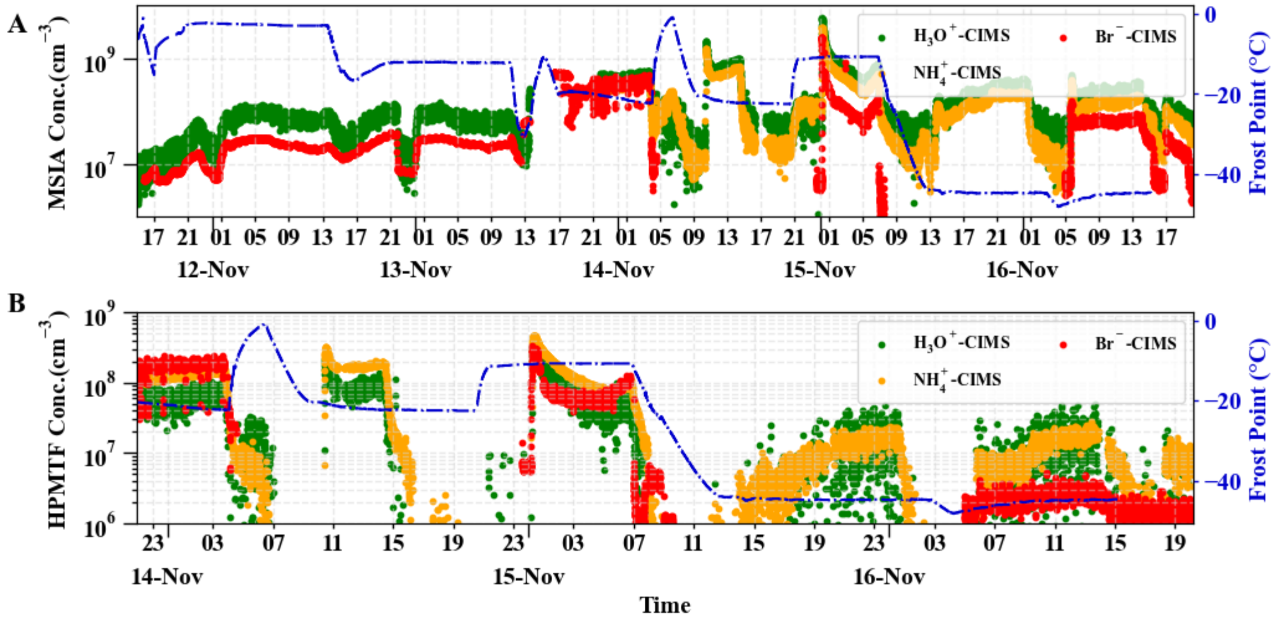

Fig. S 7. The measured MSIA and HPMTF concentrations from Br^{-}-FIGAERO$_{(g)}$-CIMS, H_3O^+-CIMS, and NH_4^+-CIMS.

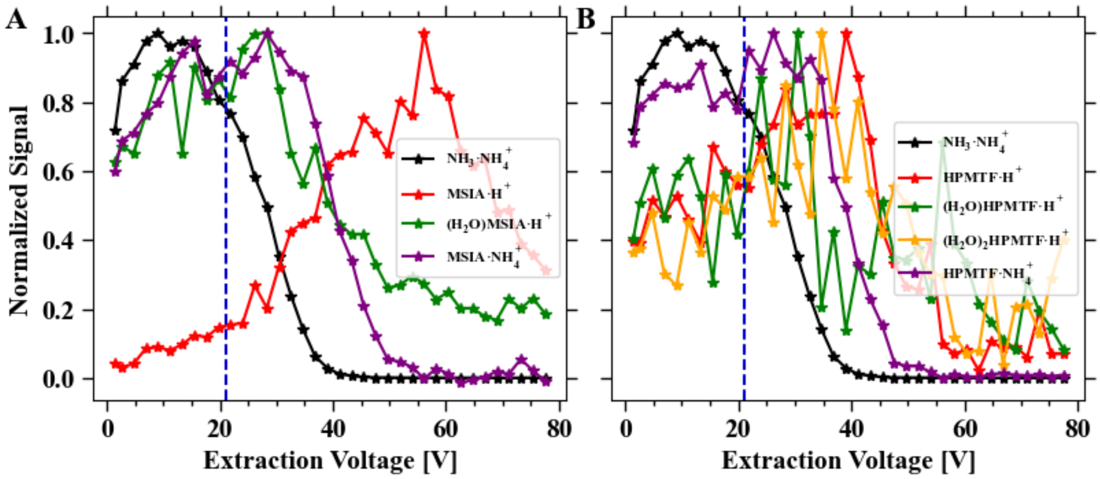

Fig. S 8. The signals for MSIA (A) and HPMTF (B) clusters observed, normalized to their respective maximum signals vs. the voltage difference in the transition region from the ion-molecule reaction region to the low-pressure mass spectrometer in H_3O^+-CIMS and NH_4^+-CIMS. The used voltage setting during all experiments was 21V, shown as a vertical line. The collision-induced dissociation of the MSIA clusters sets in at higher voltages than the used 21V and the dissociation of the $NH_3NH_4^+$ cluster, suggesting that the MSIA cluster ions are strongly bound and thus ionized at the kinetic limit, furthermore the gas-phase basicity appears strong enough for MSIA to keep the proton upon collision-induced dissociation at high voltages. This is not the case for HPMTF, but it also dissociates at higher voltages than the used 21V in both ionization schemes.

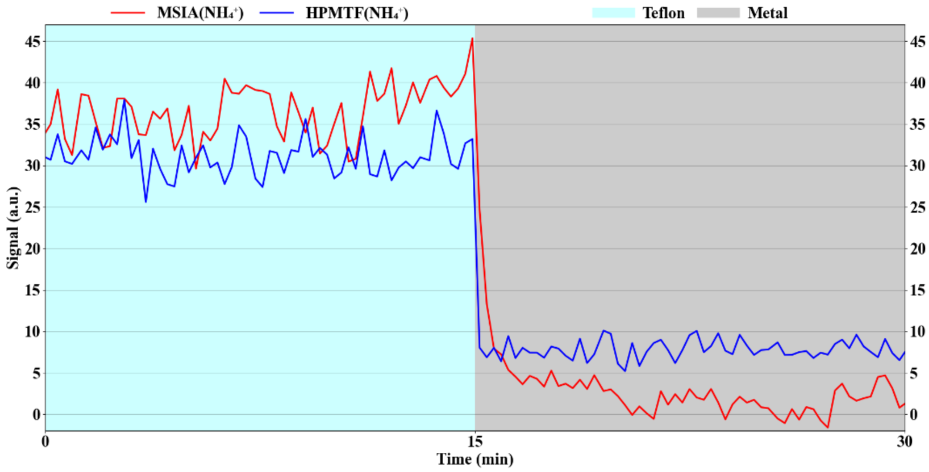

Fig. S 9. The loss of MSIA and HPMTF in a metal tube. The metal tube has a diffusional theoretical wall loss of 97.5%, which allows a maximum of 2.5% of a perfect wall loss species to get through, considering only sampled center fraction for the whole tube diameter. After going through a metal line (155cm long, 0.39cm diameter) with a total flow of 3.7 slpm, MSIA decreased to background values, while HPMTF only decreases to approximately 33% of its original value.

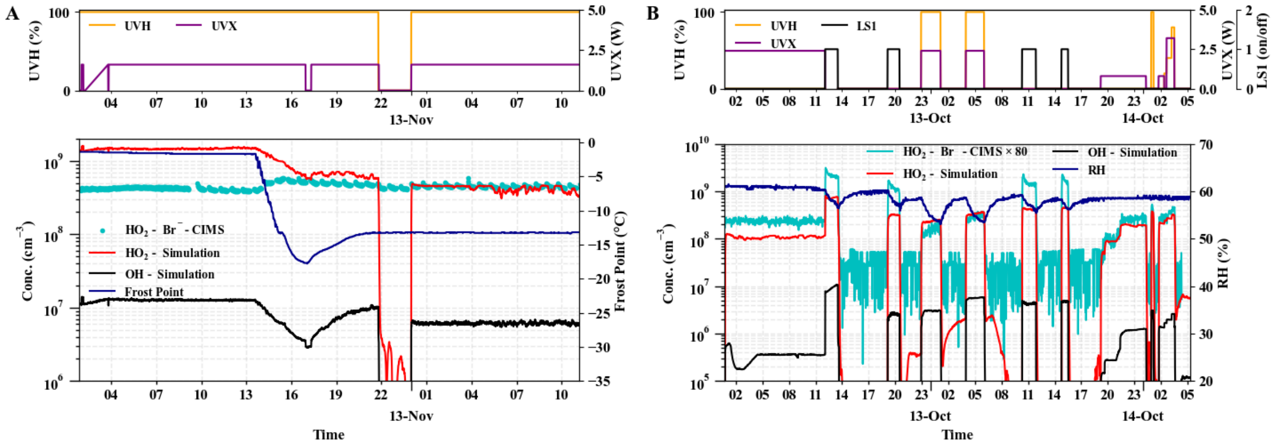

Fig. S 10. The simulated HO_2 and OH concentrations and measured HO_2 from (A) Br^--FIGAERO$_{(g)}$-CIMS in the experiment set 1, and (B) Br^--MION-CIMS in the experiment set 3. The HO_2 concentrations measured in Br^--MION-CIMS and Br^--FIGAERO$_{(g)}$-CIMS are applied with the MSA calibration factors due to the lack of direct calibration.

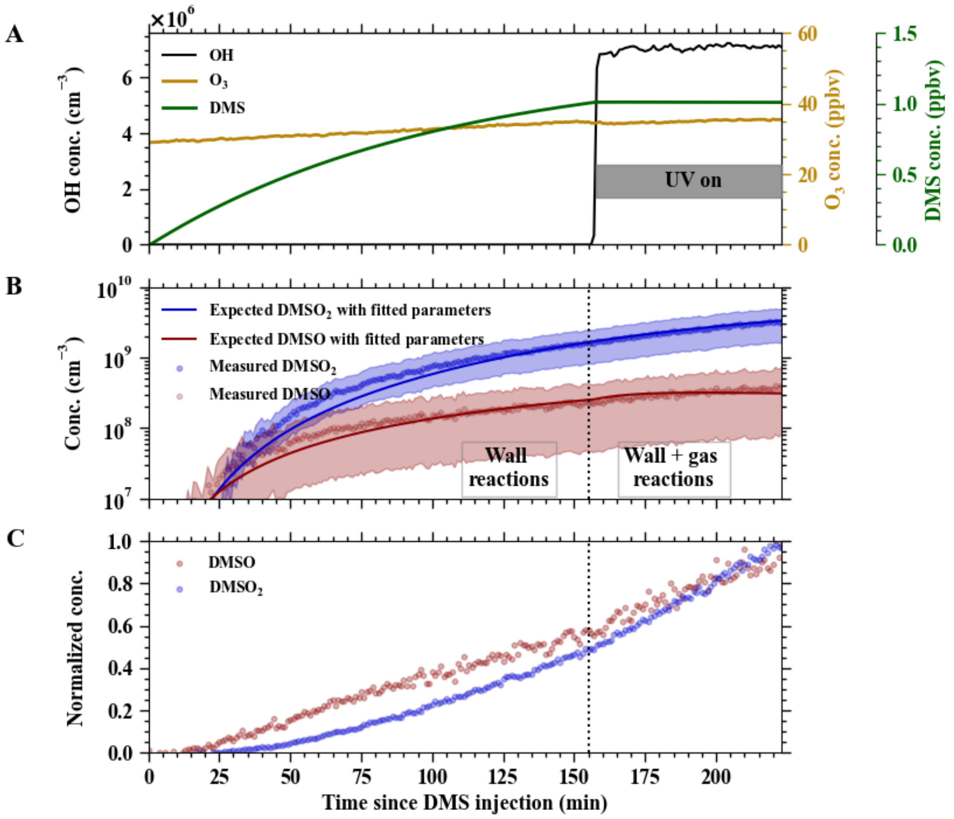

Fig. S 11. Dark experiment on wall reactions involving DMS and O_3 and photo-oxidation experiment of OH-initiated DMS oxidation. (A) OH (dark line, left axis), O_3 (dark goldenrod line, first right axis), and DMS (green line, second right axis) concentrations in the dark and photo-oxidation experiments. The OH and DMS concentrations are modeled values. (B) Measured (dots) and modeled (solid line) time series of DMSO (brown line, left axis) and $DMSO_2$ (blue line, right axis) in the dark and the photo-oxidation experiments. The dots are DMSO and $DMSO_2$ concentrations measured by NH_4^+-CIMS. (C) Appearance time of normalized concentration of DMSO and $DMSO_2$ after DMS injection. The dashed line marks turning on UV light. $\boldsymbol{k}_{\mathbf{DMS+O_{3}}_{\mathbf{wall}}} = 3.4 \times 10^{-18}$ molec^{-1} cm^{3} s^{-1} and $\boldsymbol{k}_{\mathbf{DMSO+O_{3}}_{\mathbf{wall}}} = 3 \times 10^{-15}$ molec^{-1} cm^{3} s^{-1} are used here for the simulation of the dark and photo-oxidation experiment. The subtle increase in DMSO time trace when UV lights were turned on is a consequence of substantially increased production from OH + DMS in the gas phase and substantial loss, again via OH (~ 5×10^{-4} s^{-1}).

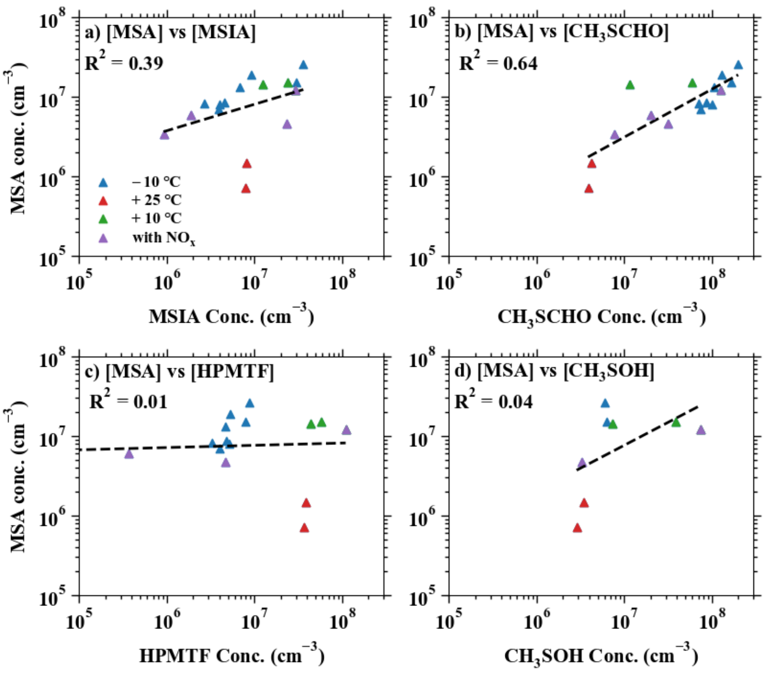

Fig. S 12. The correlation between [MSA] and a) [MSIA], b) [CH_3SCHO], c) [HPMTF], d) [CH_3SOH]. The triangles present the data collected from steady-state conditions of different OH-initiated DMS oxidation experiments listed in Table S1. The dashed lines are linear fits to the data. Blue triangles are experiments conducted at –10 °C; red triangles are experiments conducted at +25 °C; green triangles are experiments conducted at +10 °C; purple triangles refer to experiments with the presence of NO_x. As shown in a), MSA shows a positive and moderate correlation (R^2 = 0.39) with MSIA, especially for low temperatures. CH_3SCHO shows a strong and positive correlation with MSA in b).

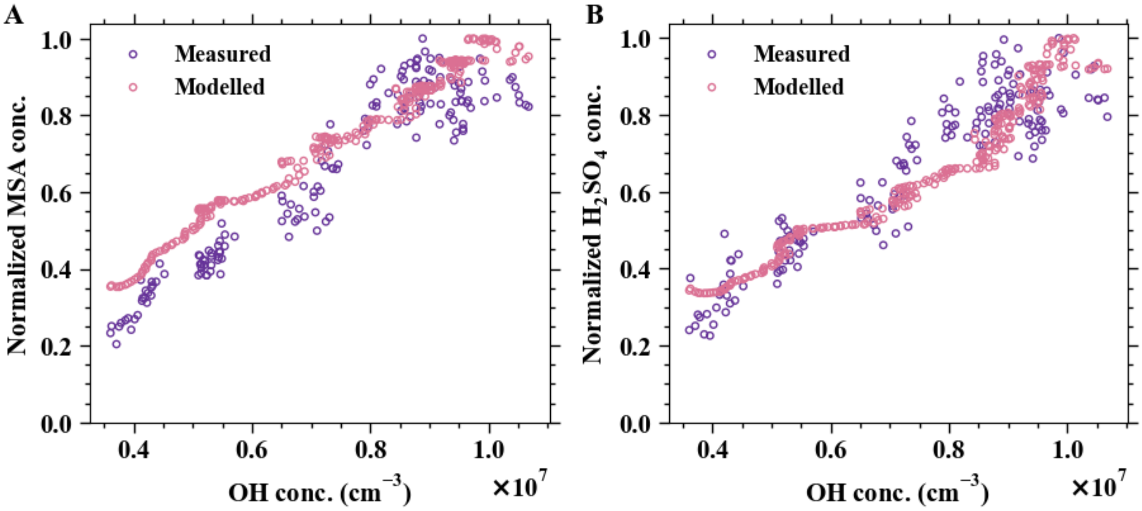

Fig. S 13. The correlation between OH and A) normalized MSA concentration, B) normalized H_2SO_4 concentration. We normalized the ratio of MSA:MSIA and H_2SO_4:MSIA to their maximum values during the OH ramping (by purging CO concentration, thus, increasing OH concentration) experiment at –10 °C. Normalized MSA and H_2SO_4 increase with the elevated OH concentration in both experiment and simulation when DMS is kept constant.

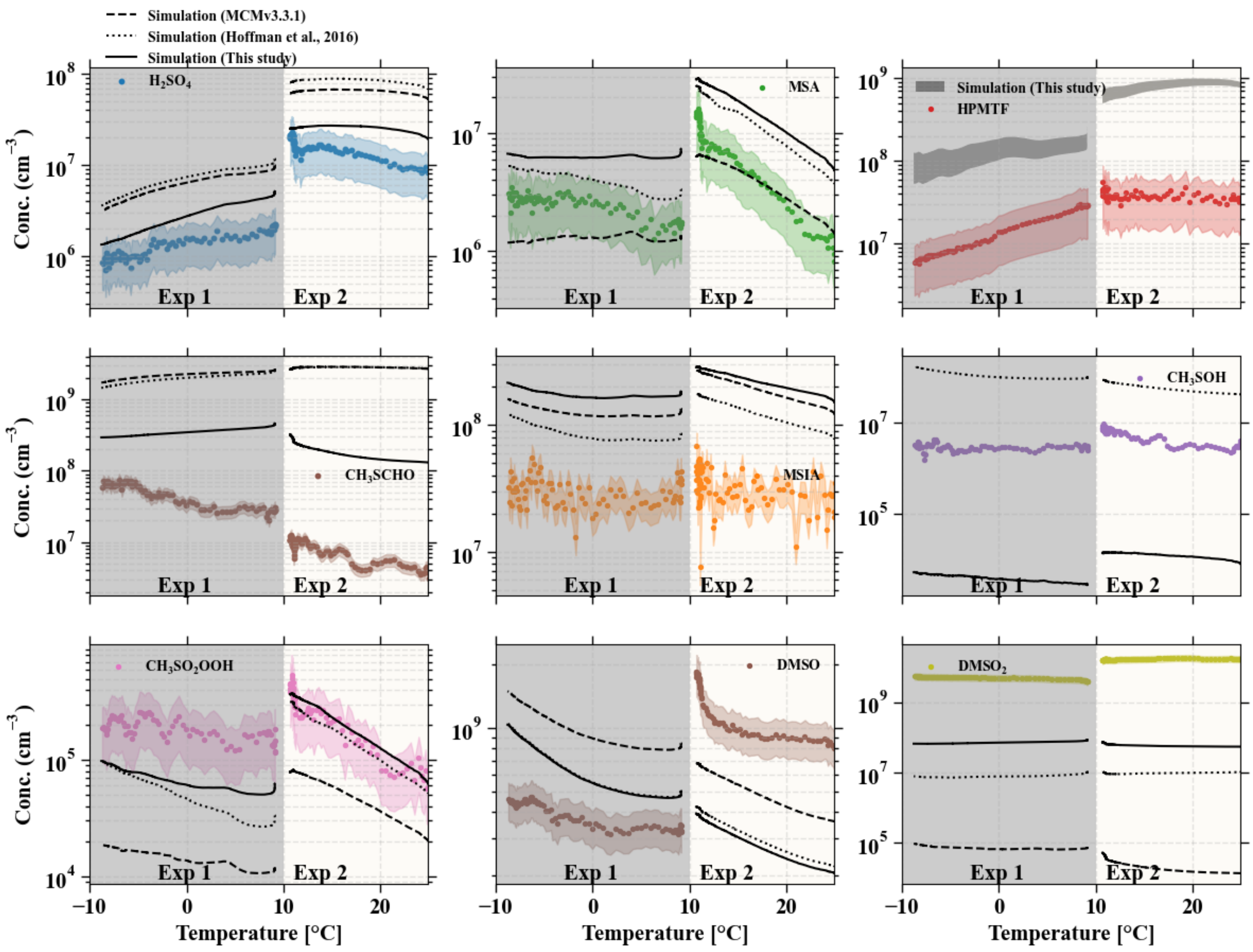

Fig. S 14. **Temperature dependence of identified species with their measured and modeled values.** Circles are identified species measured by various instruments. Experiment (Exp 2) shows the temperature dependence in the range of +25 °C to +10 °C with a light orange shade; Experiment (Exp 1) presents the temperature dependence in the range of +10 °C to –10 °C with a light grey shade. Lines represent the simulation results using the OH-initiated gas-phase oxidation mechanism from MCMv3.3.1 (dashed line, with constant NO_x concentration) and Hoffman et al., 2016 [25] (dotted line). Note that these two experiments were conducted separately with different initial concentrations of DMS, OH, and CO. Therefore, unlike the typical oxidation experiment, the temperature ramping experiment can't reach steady states for each temperature. The colored shades for different species present the uncertainties except for HPMTF, $CH_3S(O_2)OOH$, and CH_3SOH whose concentrations are lower-limit estimates; their uncertainty only includes the instrumental loss without the correction from sensitivity.

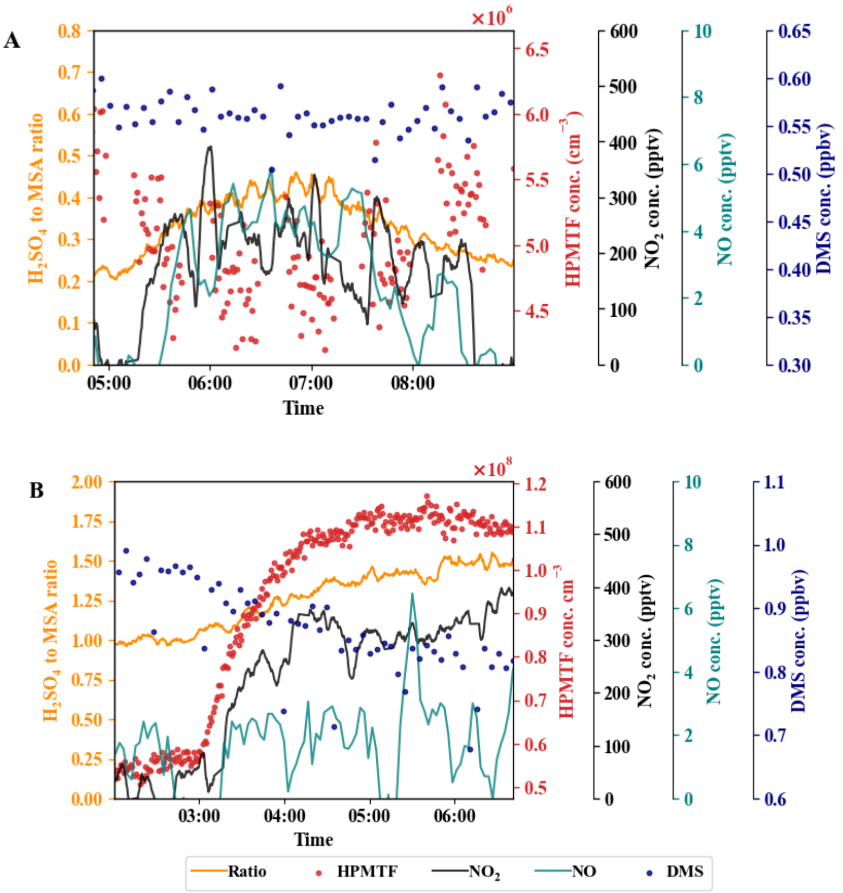

Fig. S 15. The effect of NO_x (NO_2 and NO) on the H_2SO_4:MSA. (A) Time series of H_2SO_4:MSA, NO_2, and NO at an OH-initiated DMS oxidation with increasing and decreasing NO_x at –10 °C with 120 ppbv O_3. NO_2 and NO do enhance H_2SO_4:MSA. (B) Time series of H_2SO_4:MSA, NO_2, and NO at an OH-initiated DMS oxidation with increasing NO_x at +10 °C with 280 ppbv O_3.

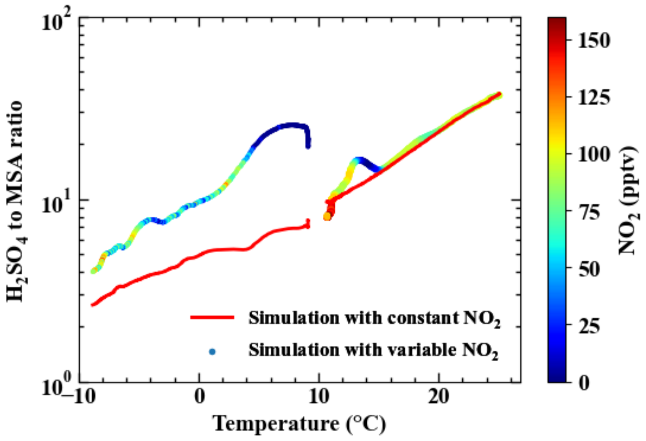

Fig. S 16. The effect of NO_2 on the modeled ratio of H_2SO_4 to MSA using MCMv3.3.1. The solid line represents the modeled H_2SO_4 to MSA with constant NO_2 (160 pptv), circles represent the modeled H_2SO_4 to MSA with varied NO_2 concentrations. It shows that the modeled H_2SO_4:MSA is sensitive to the NO_x concentration in the MCMv3.3.1 mechanism.

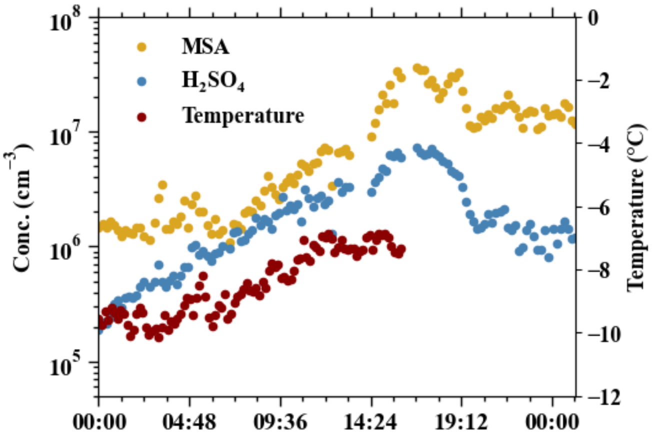

Fig. S 17. The time evolution of H_2SO_4, MSA (left axis), and temperature (right axis) on 8 May 2017 at Ny-Ålesund station.

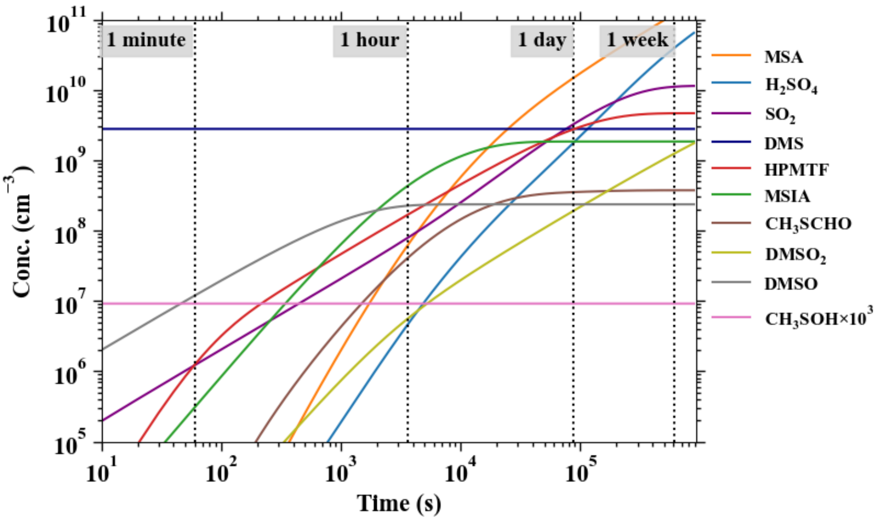

Fig. S 18. DMS oxidation simulation without wall and ventilation loss at –10 °C. We run the model without any condensation sink, wall production, wall loss, and ventilation loss. The DMS and OH concentrations are kept constant at 100 pptv and 7×10^6 cm^{-3}. NO_2 and NO are kept constant at zero. Note that, in the atmosphere, these representative total concentrations of MSA, H_2SO_4, and other species will at least partially condense and/or partition into the atmospheric aerosol, reducing gas phase concentrations.

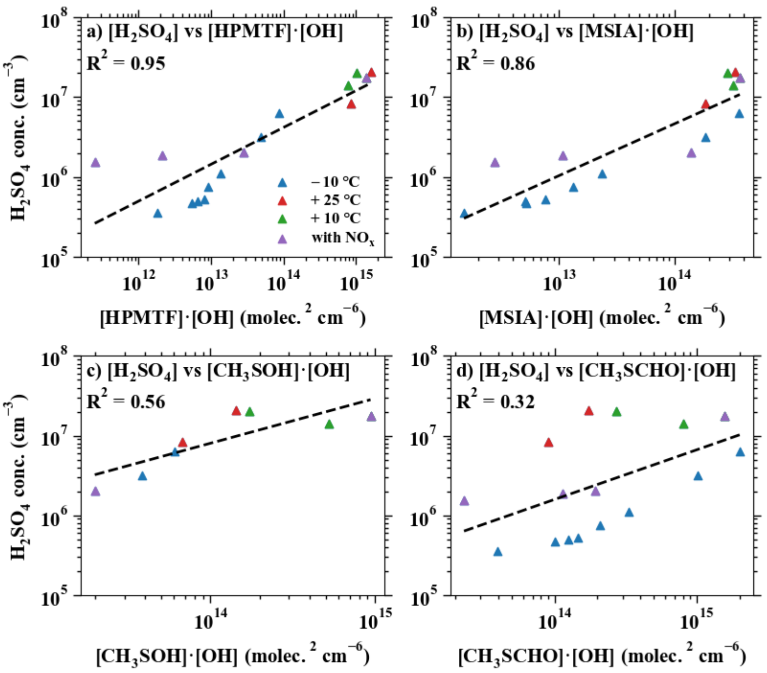

Fig. S 19. The correlation between [H_2SO_4] and a) [HPMTF] · [OH], b) [MSIA] · [OH], c) [CH_3SOH] · [OH], d) [CH_3SCHO] · [OH]. The triangles present data collected from steady-state OH-initiated DMS oxidation experiments listed in Table S1. The dashed lines are linear fits. Intermediate oxidation products such as MSIA, CH_3SCHO, and CH_3SOH are expected to be precursors of H_2SO_4. The results show strong and positive correlations between [MSIA] · [OH], [HPMTF] · [OH], [CH_3SOH] · [OH] with H_2SO_4, and a moderate correlation between [CH_3SCHO] · [OH] and H_2SO_4. These correlations agree with the mechanism in Fig. 1 (e.g., pathways 1b, 2a, and 2b).

Table S 1. Initial conditions of all experiments.

Experiment sets	Exp. no	[DMS]	Temp	$[O_3]$	[NO]	$[NO_2]$	[CO]	RH	[OH][b]	Type	Instruments
		pptv	°C	ppbv	pptv	pptv	ppbv	%	cm^{-3}		
Experiment set 1	2121.03	3914	–10	45	0	0	Bkg[a]	60	4×10^5	Steady-state oxidation	NO_3^--CIMS, Br^--MION-CIMS, H_3O^+-CIMS
	2122.01	180	–10	39	10	66	Bkg	58	3×10^6	Steady-state oxidation	
	2122.03	291	–10	109	7.5	169	Bkg	58	5.7×10^6	Steady-state oxidation	
	2123.11	2779	–10	41→ 116	0	17.8	Bkg	58	5×10^5 $\rightarrow1.2\times10^6$	OH ramping	
	2123.15	2892	–10	122	0	0	Bkg	58	1.3×10^6	Steady-state oxidation	
	2123.16	2885	–10	122	0	0	Bkg	58	1.6×10^6	Steady-state oxidation	
	2123.17	2852	–10	123	0	10.1	Bkg	58	1.8×10^6	Steady-state oxidation	
	2123.18	2818	–10	123	0	19.3	Bkg	58	2.4×10^6	Steady-state oxidation	

Experiment set 2	2262.06	1001	10	100→ 282	0	0	545	43	$1.9\times10^{6} \rightarrow 1.3\times10^{7}$	OH ramping	NO_3^--CIMS, Br^--FIGAERO$_{(g)}$-CIMS, H_3O^+-CIMS, SRI-TOF-MS
	2262.07	947	10	257	0	48.1	548	44	1.2×10^{7}	Stable from ramping	
	2262.13	689	10 → –10	254	0	0	542	18–62	$6.5\times10^{6} \rightarrow 3.5\times10^{6}$	T ramping	
	2263.01	481	–10	244	0	146	550→146	55–70	$4.3\times10^{6} \rightarrow 1.0\times10^{7}$	CO purging	
	2262.03	572	–10	122	0	0	124	70	6×10^{6}	Steady-state oxidation	
	2263.05	560	–10	121	0→6	95	116	67	6×10^{6}	NO ramping	
Experiment set 3	2294.05	650[c]	25	286	0	0	Bkg	25	4×10^{7}	Steady-state oxidation	NO_3^--CIMS, NH_4^+-CIMS
	2294.07	560[c]	25	135	0	0	Bkg	25	2.3×10^{7}	Steady-state oxidation	
	2294.09	560[c]	25→10	149	0	0	Bkg	66	2.3×10^{7}	T ramping	

[a] refers to the background level of CO concentration, where the CO comes from the ultrapure synthetic air, but there is no instrument to measure. We set 120 ppbv as the background value for simulation when the measurement is missing. [b] refers to the estimated OH concentration from the box model. [c] the DMS concentrations here are modeled values.

Table S 2. Measured decay loss in the CLOUD chamber, detection limit and uncertainty for identified species.

**Species**	**Measured loss in the CLOUD chamber (s^{-1})** Dark decay (Lights off, fan 12 %)	Cleaning stage (Lights off, fan 100 %)	**Detection limit (cm^{-3})**	**Uncertainty**	**Instruments**	**Inlet loss**
**H_2SO_4**	$(2.4 \pm 0.1) \times 10^{-3}$	5.5×10^{-3}	2×10^5	Below 50%	NO_3^--CIMS	A factor of 1.8
**MSA (CH_3SO_3H)**	$(2.2 \pm 0.03) \times 10^{-3}$	8.8×10^{-3}	2×10^5	Below 50%	NO_3^--CIMS	A factor of 1.8
**MSIA (CH_3SOOH)**	$(3.3 \pm 0.2) \times 10^{-3}$	4.9×10^{-3}	4×10^6	28%	H_3O^+-CIMS	A factor of 5.2
			3.5×10^5	58%	NH_4^+-CIMS	A factor of 5.2
			1×10^5	Lower limit estimates	Br^--MION-CIMS	A factor of 1.4
			7.5×10^6	Lower limit estimates	Br^--FIGAERO$_{(g)}$-CIMS	A factor of 2.5
**HPMTF ($HOOCH_2SCHO$)**	$(1.9 \pm 0.01) \times 10^{-3}$	3.0×10^{-3}	1.8×10^7	54%	H_3O^+-CIMS	A factor of 3.3
			4×10^6	84%	NH_4^+-CIMS	A factor of 3.3
			1×10^6	Lower limit estimates	Br^--MION-CIMS	A factor of 1.4

			1×10^{6}	Lower limit estimates	Br^{-}-FIGAERO$_{(g)}$-CIMS	A factor of 2.5
**CH_3SCHO**	$(2.4 \pm 0.1) \times 10^{-4}$, ventilation loss	3.9×10^{-4}, higher than ventilation loss	3×10^{6}	20%/ Lower limit estimates	H_3O^{+}-CIMS/NH_4^{+}-CIMS	No
**CH_3SOH**	n/a	n/a	5×10^{6}	Lower limit estimates	H_3O^{+}-CIMS	No
**$CH_3S(O)_2OOH$**	$(1.8 \pm 0.1) \times 10^{-3}$	5.5×10^{-3}	1×10^{5}	Lower limit estimates	NO_3^{-}-CIMS	No
**DMSO**	ventilation loss[a]	higher than ventilation loss	8×10^{6}	20%/80%	H_3O^{+}-CIMS/NH_4^{+}-CIMS	No
**$DMSO_2$**	ventilation loss	higher than ventilation loss	2×10^{7}	20%/50%	H_3O^{+}-CIMS/NH_4^{+}-CIMS	No

[a]Here, we cannot measure the loss for DMSO and $DMSO_2$ since the heterogeneous reactions happened on the wall all the time. We assume they are ventilation loss products in this study because they are not able to be lost to the wall rapidly due to the wall reactions.

Table S 3. Cluster formation enthalpies of different species with bromide ion and nitrate ion.

**Cluster formation pathway**	**Formation enthalpies (kcal mol^{-1})**
$CH_3SO_3H + Br^- \rightarrow CH_3SO_3H{\cdot}Br^-$	–31.8
$CH_3SOOH + Br^- \rightarrow CH_3SOOH{\cdot}Br^-$	–25.5
$HOOCH_2SCHO + Br^- \rightarrow HOOCH_2SCHO{\cdot}Br^-$	–27.1
$CH_3S(O)(O)OOH + Br^- \rightarrow CH_3S(O)(O)OOH{\cdot}Br^-$	–29.7
$H_2SO_4 + Br^- \rightarrow H_2SO_4{\cdot}Br^-$	–41.1
$CH_3SO_3H + NO_3^- \rightarrow CH_3SO_3H{\cdot}NO_3^-$	–34.4
$CH_3SOOH + NO_3^- \rightarrow CH_3SOOH{\cdot}NO_3^-$	–27.2
$HOOCH_2SCHO + NO_3^- \rightarrow HOOCH_2SCHO{\cdot}NO_3^-$	–27.5
$H_2SO_4 + NO_3^- \rightarrow H_2SO_4{\cdot}NO_3^-$	–44.7
$CH_3S(O)(O)OOH + NO_3^- \rightarrow CH_3S(O)(O)OOH{\cdot}NO_3^-$	–30.2
$HNO_3 + NO_3^- \rightarrow HNO_3{\cdot}NO_3^-$	–29.8

Table S 4. Fragmentation reaction enthalpies of different species with bromide ion and nitrate ion.

Cluster fragmentation pathway	Fragmentation enthalpies (kcal mol^{-1})
$CH_3SO_3H \cdot Br^- \rightarrow CH_3SO_3H + Br^-$	31.8
$CH_3SO_3H \cdot Br^- \rightarrow CH_3SO_3$– + HBr	25.3
$CH_3SOOH \cdot Br^- \rightarrow CH_3SOOH + Br^-$	25.5
$CH_3SOOH \cdot Br^- \rightarrow CH_3SOO^- + HBr$	35.9
$HOOCH_2SCHO \cdot Br^- \rightarrow HOOCH_2SCHO + Br^-$	27.1
$HOOCH_2SCHO \cdot Br^- \rightarrow OHCSCH_2OO^- + HBr$	55.7
$H_2SO_4 \cdot Br^- \rightarrow H_2SO_4 + Br^-$	41.1
$H_2SO_4 \cdot Br^- \rightarrow HSO_4^- + HBr$	27.9
$H_2O \cdot Br^- \rightarrow H_2O + Br^-$	13.2
$HO_2 \cdot Br^- \rightarrow HO_2 + Br^-$	23.1
$CH_3S(O)(O)OOH \cdot Br^- \rightarrow CH_3S(O)(O)OOH + Br^-$	29.7
$CH_3S(O)(O)OOH \cdot NO_3^- \rightarrow CH_3S(O)(O)OOH + NO_3^-$	30.2
$CH_3SO_3H \cdot NO_3^- \rightarrow CH_3SO_3H + NO_3^-$	34.4
$CH_3SO_3H \cdot NO_3^- \rightarrow CH_3SO_3^- + HNO_3$	28.5
$CH_3SOOH \cdot NO_3^- \rightarrow CH_3SOOH + NO_3^-$	27.2
$HOOCH_2SCHO \cdot NO_3^- \rightarrow HOOCH_2SCHO + NO_3^-$	27.5
$H_2SO_4 \cdot NO_3^- \rightarrow H_2SO_4 + NO_3^-$	44.7
$H_2SO_4 \cdot NO_3^- \rightarrow HSO_4^- + HNO_3$	32.0
$HNO_3 \cdot NO_3^- \rightarrow HNO_3 + NO_3^-$	29.8

Table S 5. MSA and H_2SO_4 concentrations from OH-initiated DMS oxidation experiments and field measurement.

		DMS (ppbv)	OH[f] (cm^{-3})	O_3 (ppbv)	NO (pptv)	MSA (cm^{-3})	H_2SO_4 (cm^{-3})	$DMSO_2$ (cm^{-3})	DMSO (cm^{-3})	Temp (°C)	Location
This study	1	0.2	3×10^{6}	40	6	3.3×10^{6}	1.5×10^{6}	5.2×10^{9}	9.2×10^{8}	–10	CLOUD chamber
	2	0.3	6×10^{6}	101	10	6×10^{6}	2×10^{6}	3.7×10^{9}	7×10^{8}	–10	CLOUD chamber
	3	0.6	6×10^{6}	124	0	1.7×10^{7}	3×10^{6}	6.5×10^{9}	1×10^{9}	–10	CLOUD chamber
Field measurement	4[a]	n/a	n/a	n/a	n/a	1×10^{6}–4×10^{7}	2×10^{5}–7×10^{6}	n/a	n/a	–10 to –6	Ny-Ålesund
	5[b]	0.1–0.5	n/a	n/a	n/a	n/a	n/a	2.5×10^{8}–3×10^{9}	3×10^{9}–5×10^{9}	n/a	Arabian Sea
	6[c]	n/a	n/a	n/a	n/a	3.5×10^{7} – 6.8×10^{7}	n/a	n/a	n/a	−10 to 5	over Southern Ocean
	7[d]	n/a	0–2×10^{6}	n/a	n/a	0–6.5×10^{6}	0–1.4×10^{7}	n/a	n/a	Above 10	Mace Head
	8[e]	n/a	n/a	n/a	n/a	2×10^{6}–1.9×10^{7}	1.1×10^{6}–4.7×10^{6}	n/a	n/a	−8.3 to 7	Southern Ocean

[a] Beck et al., 2021 [26] (8 May 2017), [b] Edtbauer A et al., 2020 [49], [c] Yan et al., 2019 [50], [d] Berresheim, et al., 2002 [51] on 17 June 1999. [e] Jokinen et al., 2018 [52] (16 December 2014), [f] OH concentrations are simulated results. Note: n/a means data are not available.

Table S 6. The ratio of analytes' detection sensitivity compared to maximum sensitivity in Br^--MION-CIMS and Iodide-CIMS.

**Species**	**Br^--MION-CIMS**	**Iodide-CIMS**
H_2SO_4	1[a]	
N_2O_5		1[c]
Cl_2	0.11[a]	1[c]
HOI	0.12[a]	
I_2	1[a]	
Br_2		1[c]
HPMTF	*<1 (assumed)*	1[c], 0.0078[d]
HNO_3	0.2[b]	0.5e

a. Wang et al., 2021 [16]; b. Wang et al., 2020 [53]; c. Veres et al., 2020 [21]; d. Ye et al., 2021 [22]; e. Lee et al., 2014 [54].

Table S 7. Reactions of DMS oxidation chemistry used in this study.

Reaction	k (Reaction rate coefficients)	Reference
DMS + OH → $CH_3SCH_2O_2$	$1.12 \times 10^{-11} \times \exp(–250 / T)$	a
DMS + OH → CH_3SOHCH_3	$9.5 \times 10^{–39} \times O_2 \times \exp(5270 / T) / (1 + 7.5 \times 10^{–29} \times O_2 \times \exp(5610 / T))$	a
CH_3SOHCH_3 → DMS + OH	$1.7 \times 10^{–42} \times O_2 \times \exp(7810 / T) / (1 + 5.5 \times 10^{–31} \times O_2 \times \exp(7460 / T)) / (8.3 \times 10^{–29} \times T \times \exp(5136 / T))$	f
CH_3SOHCH_3 → $HODMSO_2$	$8.50 \times 10^{–13} \times O_2$	g
CH_3SOHCH_3 → CH_3SOH + CH_3O_2	5.00×10^{5}	a
DMS + NO_3 → $CH_3SCH_2O_2$ + HNO_3	$1.9 \times 10^{–13} \times \exp(520 / T)$	a
$CH_3SCH_2O_2$ + HO_2 → CH_3SCH_2OOH	$KRO_2HO_2 \times 0.387$	a
$CH_3SCH_2O_2$ + NO → CH_3SCH_2O + NO_2	$4.9 \times 10^{–12} \times \exp(260 / T)$	a
$CH_3SCH_2O_2$ + NO_3 → CH_3SCH_2O + NO_2	$2.3 \times 10^{–12}$	a
$CH_3SCH_2O_2$ → 0.8 CH_3SCH_2O + 0.1 CH_3SCH_2OH + 0.1 CH_3SCHO	$3.74 \times 10^{–12} \times RO_2$	a
$HODMSO_2$ + NO → $DMSO_2$ + HO_2 + NO_2	$2.7 \times 10^{–12} \times \exp(360 / T)$	a
$HODMSO_2$ → DMSO + HO_2	$8.90 \times 10^{10} \times \exp(–6040 / T)$	a
CH_3SCH_2OOH + OH → CH_3SCHO + OH + H_2O	$7.03 \times 10^{–11}$	a
CH_3SCH_2O → CH_3S + HCHO	1.00×10^{6}	a
CH_3SCH_2OH + OH → CH_3SCHO + HO_2	$2.78 \times 10^{–11}$	a
CH_3SCHO + OH → CH_3S + CO + H_2O	$1.11 \times 10^{–11}$	a
$DMSO_2$ + OH → $DMSO_2O_2$ + H_2O	$4.40 \times 10^{–14}$	a
DMSO + OH → MSIA + CH_3O_2	$6.10 \times 10^{–12} \times \exp(800 / T)$	a
DMSO + OH → $DMSO_2$	$6.10 \times 10^{–12} \times \exp(800 / T) / 90$	k
DMSO + NO_3 → $DMSO_2$ + NO_2	$2.9 \times 10^{–13}$	g
$CH_3SOCH_2O_2$ + NO → CH_3SO + HCHO + NO_2	$7.5 \times 10^{–12}$	a
$CH_3SOCH_2O_2$ + HO_2 → CH_3SOCH_2OOH	$1.5 \times 10^{–12}$	a
CH_3SOH + OH → CH_3SO + H_2O	$5.00 \times 10^{–11}$	a
CH_3S + NO_2 → CH_3SO + NO	$6.00 \times 10^{–11} \times \exp(240 / T)$	a
CH_3S + O_3 → CH_3SO	$1.15 \times 10^{–12} \times \exp(430 / T)$	a
CH_3S → CH_3SOO	$1.20 \times 10^{–16} \times \exp(1580 / T) \times O_2$	a
$DMSO_2O_2$ + HO_2 → $DMSO_2OOH$	$1.13 \times 10^{–13} \times \exp(1300 / T)$	a
$DMSO_2O_2$ + NO → $DMSO_2O$ + NO_2	$2.7 \times 10^{–12} \times \exp(360 / T)$	a
$DMSO_2O_2$ + NO_3 → $DMSO_2O$ + NO_2	$2.3 \times 10^{–12}$	a
$DMSO_2O_2$ → 0.2 CH_3SO_2CHO + 0.6 $DMSO_2O$ + 0.2 $DMSO_2OH$	$2.0 \times 10^{–12} \times RO_2$	a
MSIA + OH → CH_3SO_2 + H_2O	$1.60 \times 10^{–11}$	h
MSIA + NO_3 → CH_3SO_2 + HNO_3	$1.00 \times 10^{–13}$	h

$CH_3SO + NO_2 \rightarrow 0.25\ CH_3O_2 + 0.25\ SO_2 + 0.25\ NO + 0.75\ CH_3SO_2 + 0.75\ NO$	1.20×10^{-11}	a
$CH_3SO + O_3 \rightarrow CH_3O_2 + SO_2$	6.00×10^{-13}	d
$CH_3SO \rightarrow CH_3SOO_2$	$3.12 \times 10^{-16} \times \exp(1580 / T) \times O_2$	a
$CH_3SOO + NO \rightarrow CH_3SO + NO_2$	1.1×10^{-11}	a
$CH_3SOO + NO_2 \rightarrow CH_3SO + NO_3$	2.2×10^{-11}	a
$CH_3SOO + HO_2 \rightarrow CH_3SOOH$	4.00×10^{-12}	a
$CH_3SOO \rightarrow CH_3S$	$3.50 \times 10^{10} \times \exp(-3560 / T)$	a
$CH_3SOO \rightarrow CH_3O_2 + SO_2$	$5.6 \times 10^{16} \times \exp(-10870 / T)$	a
$CH_3SOO \rightarrow CH_3SO_2$	1.00	I
$DMSO_2OOH + OH \rightarrow CH_3SO_2CHO + OH + H_2O$	1.26×10^{-12}	a
$DMSO_2OOH + OH \rightarrow DMSO_2O_2 + H_2O$	3.60×10^{-12}	a
$DMSO_2O \rightarrow CH_3SO_2 + HCHO$	1.00×10^{6}	a
$CH_3SO_2CHO + OH \rightarrow CH_3SO_2 + CO + H_2O$	1.78×10^{-12}	a
$DMSO_2OH + OH \rightarrow CH_3SO_2CHO + HO_2 + H_2O$	5.23×10^{-13}	a
$DMSO_2OH + OH \rightarrow DMSO_2O + H_2O$	1.40×10^{-13}	a
$CH_3SO_2 + OH \rightarrow MSA$	5.00×10^{-11}	h
$CH_3SO_2 + NO_2 \rightarrow CH_3SO_3 + NO$	2.20×10^{-11}	g
$CH_3SO_2 + O_3 \rightarrow CH_3SO_3 + O_3$	3.00×10^{-13}	a
$CH_3SO_2 \rightarrow CH_3O_2 + SO_2$	$8.80 \times 10^{14} \times \exp(-9673 / T)$	c
$CH_3SO_2 \rightarrow CH_3SO_2O_2$	$1.03 \times 10^{-16} \times \exp(1580 / T) \times O_2$	a
$CH_3SOO_2 + HO_2 \rightarrow 0.44\ CH_3SO_2 + 0.44\ OH + 0.41\ CH_3SOOOH + 0.15\ MSIA + 0.15\ O_3$	$5.2 \times 10^{-13} \times \exp(980 / T)$	a
$CH_3SOO_2 + NO \rightarrow CH_3SO_2 + NO_2$	1.00×10^{-11}	a
$CH_3SOO_2 + NO_2 \rightarrow CH_3SOO_2NO_2$	$1.20 \times 10^{-12} \times (T / 300)^{-0.9}$	a
$CH_3SOO_2 + NO_3 \rightarrow CH_3SO_2 + NO_2$	4.002×10^{-12}	a
$CH_3SOO_2 \rightarrow CH_3SO$	$9.10 \times 10^{10} \times \exp(-3560 / T)$	a
$CH_3SOO_2 \rightarrow 0.7\ CH_3SO_2 + 0.3\ MSIA$	$1.00 \times 10^{-11} \times RO_2$	a
$CH_3SO_3 + HO_2 \rightarrow MSA$	5.00×10^{-11}	a
$CH_3SO_3 \rightarrow CH_3O_2 + SO_3\text{-}H_2O$	$5.00 \times 10^{13} \times \exp(-9946 / T)$	a
$CH_3SO_2O_2 + HO_2 \rightarrow 0.41\ CH_3SO_2OOH + 0.44\ CH_3SO_3 + 0.44\ OH + 0.15\ MSA + 0.15\ O_3$	$5.2 \times 10^{-13} \times \exp(980 / T)$	a
$CH_3SO_2O_2 + NO \rightarrow CH_3SO_3 + NO_2$	1.00×10^{-11}	a
$CH_3SO_2O_2 + NO_2 \rightarrow CH_3SO_4NO_2$	$1.20 \times 10^{-12} \times (T / 300)^{-0.9}$	a
$CH_3SO_2O_2 + NO_3 \rightarrow CH_3SO_3 + NO_2$	4.002×10^{-12}	a
$CH_3SO_2O_2 \rightarrow CH_3SO_2$	$3.01 \times 10^{10} \times \exp(-3560 / T)$	a
$CH_3SO_2O_2 \rightarrow 0.7\ CH_3SO_3 + 0.3\ MSA$	$1.00 \times 10^{-11} \times RO_2$	a
$CH_3SOOOH + OH \rightarrow CH_3SOO_2 + H_2O$	9.00×10^{-11}	a

$CH_3SOO_2NO_2 + OH \rightarrow MSIA + NO_2$	1.00×10^{-11}	a
$CH_3SOO_2NO_2 \rightarrow CH_3SOO_2 + NO_2$	$5.40 \times 10^{16} \times \exp(-13112 / T)$	a
$MSA + OH \rightarrow CH_3SO_3 + H_2O$	2.24×10^{-14}	a
$CH_3SO_2OOH + OH \rightarrow CH_3SO_2O_2 + H_2O$	3.60×10^{-12}	a
$CH_3SO_4NO_2 + OH \rightarrow CH_3SO_2O_2 + HNO_3$	3.60×10^{-13}	a
$CH_3SO_4NO_2 \rightarrow CH_3SO_2O_2 + NO_2$	$5.40 \times 10^{16} \times \exp(-13112 / T)$	a
$O \rightarrow O_3$	$5.6 \times 10^{-34} \times N_2 \times (T / 300)^{-2.6} \times O_2$	a
$O + O_3 \rightarrow$ dummy	$8.0 \times 10^{-12} \times \exp(-2060 / T)$	a
$O + NO \rightarrow NO_2$	KMT01	a
$O + NO_2 \rightarrow NO$	$5.5 \times 10^{-12} \times \exp(188 / T)$	a
$O + NO_2 \rightarrow NO_3$	KMT02	a
$O^1D \rightarrow O$	$3.2 \times 10^{-11} \times \exp(67 / T) \times O_2$	a
$NO + O_3 \rightarrow NO_2$	$1.4 \times 10^{-12} \times \exp(-1310 / T)$	a
$NO_2 + O_3 \rightarrow NO_3$	$1.4 \times 10^{-13} \times \exp(-2470 / T)$	a
$NO + NO \rightarrow NO_2 + NO_2$	$3.3 \times 10^{-39} \times \exp(530 / T) \times O_2$	a
$NO + NO_3 \rightarrow NO_2 + NO_2$	$1.8 \times 10^{-11} \times \exp(110 / T)$	a
$NO_2 + NO_3 \rightarrow NO + NO_2$	$4.50 \times 10^{-14} \times \exp(-1260 / T)$	a
$NO_2 + NO_3 \rightarrow N_2O_5$	KMT03	a
$O^1D \rightarrow OH + OH$	$2.14 \times 10^{-10} \times H_2O$	a
$OH + O_3 \rightarrow HO_2$	$1.70 \times 10^{-12} \times \exp(-940 / T)$	a
$OH + H_2 \rightarrow HO_2$	$7.7 \times 10^{-12} \times \exp(-2100 / T)$	a
$OH + CO \rightarrow HO_2$	KMT05	a
$OH + H_2O_2 \rightarrow HO_2$	$2.9 \times 10^{-12} \times \exp(-160 / T)$	a
$HO_2 + O_3 \rightarrow OH$	$2.03 \times 10^{-16} \times (T / 300)^{4.57} \times \exp(693 / T)$	a
$OH + HO_2 \rightarrow$ dummy	$4.8 \times 10^{-11} \times \exp(250 / T)$	a
$HO_2 + HO_2 \rightarrow H_2O_2$	$2.20 \times 10^{-3} \times KMT06 \times \exp(600 / T) + 1.90 \times 10^{-33} \times M \times KMT06 \times \exp(980 / T)$	a
$OH + NO \rightarrow HONO$	KMT07	a
$OH + NO_2 \rightarrow HNO_3$	KMT08	a
$OH + NO_3 \rightarrow HO_2 + NO_2$	2.0×10^{-11}	a
$HO_2 + NO \rightarrow OH + NO_2$	$3.45 \times 10^{-12} \times \exp(270 / T)$	a
$HO_2 + NO_2 \rightarrow HO_2NO_2$	KMT09	a
$OH + HO_2NO_2 \rightarrow NO_2$	$3.2 \times 10^{-13} \times \exp(690 / T) \times 1.0$	a
$HO_2 + NO_3 \rightarrow OH + NO_2$	4.0×10^{-12}	a
$OH + HONO \rightarrow NO_2$	$2.5 \times 10^{-12} \times \exp(260 / T)$	a
$OH + HNO_3 \rightarrow NO_3$	KMT11	a
$O + SO_2 \rightarrow SO_3$	$4.0 \times 10^{-32} \times \exp(-1000 / T) \times M$	a
$OH + SO_2 \rightarrow HSO_3$	KMT12	a
$HSO_3 \rightarrow HO_2 + SO_3$	$1.3 \times 10^{-12} \times \exp(-330 / T) \times O_2$	a
$HNO_3 \rightarrow NA$	6.00×10^{-6}	a

$N_2O_5 \rightarrow NA + NA$	4.00×10^{-4}	a
$SO_3 \rightarrow SA$	$1.20 \times 10^{-15} \times H_2O$	a
$O_3 \rightarrow O^1D$	J(1)	a
$N_2O_5 \rightarrow NO_2 + NO_3$	KMT04	a
$HO_2NO_2 \rightarrow HO_2 + NO_2$	KMT10	a
$CH_3SCH_2O_2 \rightarrow HOOCH_2SCH_2O_2$	$2.2 \times 10^{11} \times \exp(-9.8 \times 10^3 / T) \times \exp(1.0 \times 10^8 / T^3) \times 5$	j, e
$HOOCH_2SCH_2O_2 \rightarrow HOOCH_2SCHO + OH$	$6.1 \times 10^{11} \times \exp(-9.5 \times 10^3 / T+1.1 \times 10^8 / T^3)$	j
$HOOCH_2SCH_2O_2 + NO \rightarrow HOOCH_2SCH_2O + NO_2$	$4.9 \times 10^{-12} \times \exp(260 / T)$	c
$HOOCH_2SCH_2O + NO \rightarrow HOOCH_2S + CH_2O$	1.0×10^6	c
$HOOCH_2SCH_2O_2 + HO_2 \rightarrow HOOCH_2SCH_2OOH$	$1.13 \times 10^{-13} \times \exp(1300 / T)$	c
$HOOCH_2SCHO + OH \rightarrow HOOCH_2SCO + H_2O$	1.4×10^{-12}	c
$HOOCH_2SCO \rightarrow HOOCH_2S + CO$	$9.2 \times 10^9 \times \exp(-505.4 / T)$	c
$HOOCH_2SCO \rightarrow OH + CH_2O + OCS$	$1.6 \times 10^7 \times \exp(-1468.6 / T)$	c
$HOOCH_2S + O_3 \rightarrow HOOCH_2SO$	$1.15 \times 10^{-12} \times \exp(430 / T)$	c
$HOOCH_2S + NO_2 \rightarrow HOOCH_2SO + NO$	$6.0 \times 10^{-11} \times \exp(240 / T)$	c
$HOOCH_2SO + O_3 \rightarrow SO_2 + CH_2O + OH$	4.0×10^{-13}	c
$HOOCH_2SO + NO_2 \rightarrow SO_2 + CH_2O + OH + NO$	1.2×10^{-11}	c
$H \rightarrow HO_2$	$4.3 \times 10^{-32} \times (T / 300)^{-1.2} \times M \times O_2$	b
$O + OH \rightarrow H$	$2.4 \times 10^{-11} \times \exp(110 / T)$	b
$O + HO_2 \rightarrow OH$	$2.7 \times 10^{-11} \times \exp(224 / T)$	b
$O + H_2O_2 \rightarrow HO_2 + OH$	$1.4 \times 10^{-12} \times \exp(-2000 / T)$	b
$OH + OH \rightarrow H_2O + O$	$6.2 \times 10^{-14} \times \exp(945 / T) \times (T / 298)^{2.6}$	b
$OH + OH \rightarrow H_2O_2$	$9.0 \times 10^{-31} \times (T / 300)^{-3.2} \times M$	b
$O^1D + O_3 \rightarrow O + O$	2.4×10^{-10}	b
$MSIA + O_3 \rightarrow MSA$	2.00×10^{-18}	f
$CH_3SOH + O_3 \rightarrow CH_3O_2 + HO_2 + SO_2$	2.00×10^{-12}	d
$CH_3O_2 + HO_2 \rightarrow CH_3OOH$	$3.8 \times 10^{-13} \times \exp(780 / T) \times (1-1 / (1 + 498 \times \exp(-1160 / T)))$	b
$CH_3O_2 + HO_2 \rightarrow HCHO$	$3.8 \times 10^{-13} \times \exp(780 / T) \times (1 / (1 + 498 \times \exp(-1160 / T)))$	b
$CH_3O_2 + NO \rightarrow CH_3ONO_2$	$2.3 \times 10^{-12} \times \exp(360 / T) \times 0.004$	b
$CH_3O_2 + NO \rightarrow CH_3O + NO_2$	$2.3 \times 10^{-12} \times \exp(360 / T) \times 0.996$	b
$CH_3O_2 + NO_2 \rightarrow CH_3O_2NO_2$	$2.5 \times 10^{-30} \times (T / 300)^{-5.5} \times M \times 0.78$	b
$CH_3O_2 + NO_3 \rightarrow CH_3O + NO_2$	1.2×10^{-12}	b
$CH_3O_2 + CH_3O_2 \rightarrow CH_3O$	$2 \times 7.4 \times 10^{-13} \times \exp(-520 / T)$	a
$CH_3O_2 + CH_3O_2 \rightarrow CH_3OH + HCHO$	$1.03 \times 10^{-13} \times \exp(365 / T) \times 0.7$	a

$CH_3O_2 + CH_3O_2 \rightarrow CH_3OOCH_3$	$0.06 \times 1.03 \times 10^{-13} \times \exp(365 / T)$	b
$OH + CH_3OOH \rightarrow CH_3O_2$	$5.3 \times 10^{-12} \times \exp(190 / T) \times 0.6$	b
$OH + CH_3OOH \rightarrow CH_2OOH$	$5.3 \times 10^{-12} \times \exp(190 / T) \times 0.4$	b
$CH_3O \rightarrow HCHO + HO_2$	$7.2 \times 10^{-14} \times \exp(-1080 / T) \times O_2$	a
$CH_3O_2NO_2 \rightarrow CH_3O_2 + NO_2$	$9.0 \times 10^{-5} \times \exp(-9690 / T) \times M \times 0.78$	b
$CH_3OH + OH \rightarrow HO_2 + HCHO$	$2.85 \times 10^{-12} \times \exp(-345 / T)$	a
$CH_3O_2 + OH \rightarrow CH_3O + HO_2$	$3.7 \times 10^{-11} \times \exp(350 / T) \times 0.8$	b
$CH_3O_2 + OH \rightarrow CH_2O_2$	$3.7 \times 10^{-11} \times \exp(350 / T) \times 0.05$	b
$NO_3 + HCHO \rightarrow HNO_3 + CO + HO_2$	5.5×10^{-16}	a
$OH + HCHO \rightarrow HO_2 + CO$	$5.4 \times 10^{-12} \times \exp(135 / T)$	a
$DMS + O_3 \rightarrow DMSO_w$	3.4×10^{-18}	k
$DMSO_w + O_3 \rightarrow DMSO_{2w} + DMSO_w$	3×10^{-15}	k
$DMSO_w + OH \rightarrow MSIA + CH_3O_2$	$6.1 \times 10^{-12} \times \exp(800 / T)$	a
$DMSO_w + OH \rightarrow DMSO_2$	$6.1 \times 10^{-12} \times \exp(800 / T) / 90$	k
$DMSO_w + NO_3 \rightarrow DMSO_2 + NO_2$	2.9×10^{-13}	g
$DMSO_{2w} + OH \rightarrow DMSO_2O_2 + H_2O$	4.4×10^{-14}	a

a MCMv3.3.1[23]. b IUPAC. c [25]. d [27]. e [26]. f [37]. g [55]. h [31]. I [24]. J [21]. k is this study.

Notes:

1) $DMSO_{2w}$ and $DMSO_w$ refer to the gaseous $DMSO_2$ and DMSO formed from wall reactions.

2) M is the number of molecules per cubic centimetre.

3) J(1) is the O_3 photolysis rate.

4) The photolysis rate of O_3 for full intensity of UV is around $6 \times 10^{-5}\ s^{-1}$.

5) $RO_2 = CH_3SCH_2O_2 + HODMSO_2 + DMSO_2O_2 + CH_3O_2 + CH_3SOO + CH_3SOO_2 + CH_3SO_2O_2 + HOOCH_2SCH_2O_2$

6) $K10 = 1.0 \times 10^{-31} \times M \times (T / 300)^{-1.6}$; $K1I = 5.0 \times 10^{-11} \times (T / 300)^{-0.3}$; KR1 = K10 / K1I; FC1 = 0.85; $NC1 = 0.75 - 1.27 \times (\log_{10}(FC1))$; $F1 = 10^{(\log10(FC1) / (1 + (\log10(KR1) / NC1)2))}$; KMT01 = (K10 × K1I) × F1 / (K10 + K1I) ; $K20 = 1.3 \times 10^{-31} \times M \times (T / 300)^{-1.5}$; $K2I = 2.3 \times 10^{-11} \times (T / 300)^{0.24}$; KR2 = K20 / K2I; FC2 = 0.6; $NC2 = 0.75 - 1.27 \times (\log_{10}(FC2))$; $F2 = 10^{(\log10(FC2) / (1 + (\log10(KR2) / NC2)2))}$; KMT02 = (K20 × K2I) × F2 / (K20 + K2I) ; $K30 = 3.6 \times 10^{-30} \times M \times (T / 300)^{-4.1}$; $K3I = 1.9 \times 10^{-12} \times (T / 300)^{0.2}$; KR3 = K30 / K3I; FC3 = 0.35; $NC3 = 0.75 - 1.27 \times (\log_{10}(FC3))$; $F3 = 10^{(\log10(FC3) / (1 + (\log10(KR3) / NC3)2))}$; KMT03 = (K30 × K3I) × F3 / (K30 + K3I) ; $K40 = 1.3 \times 10^{-3} \times M \times (T / 300)^{-3.5} \times \exp(-11000 / T)$; $K4I = 9.7 \times 10^{14} \times (T / 300)^{0.1} \times \exp(-11080 / T)$; KR4 = K40 / K4I; FC4 = 0.35; $NC4 = 0.75 - 1.27 \times (\log_{10}(FC4))$; $F4 = 10^{(\log10(FC4) / (1 + (\log10(KR4) / NC4)2))}$; KMT04 = (K40 × K4I) × F4 / (K40 + K4I) ; $KMT05 = 1.44 \times 10^{-13} \times (1 + (M / 4.2 \times 10^{19}))$; $KMT06 = 1 + (1.40 \times 10^{-21} \times \exp(2200 / T) \times H_2O)$; $K70 = 7.4 \times 10^{-31} \times M \times (T / 300)^{-2.4}$; $K7I = 3.3 \times 10^{-11} \times (T / 300)^{-0.3}$; KR7 = K70 / K7I; FC7 = 0.81; $NC7 = 0.75 - 1.27 \times (\log_{10}(FC7))$; $F7 = 10^{(\log10(FC7) / (1 + (\log10(KR7) / NC7)2))}$; KMT07 = (K70 × K7I) × F7 / (K70 + K7I) ; $K80 = 3.2 \times 10^{-30} \times M \times (T / 300)^{-4.5}$; $K8I = 3.0 \times 10^{-11}$; KR8 = K80 / K8I; FC8 = 0.4; $NC8 = 0.75 - 1.27 \times (\log_{10}(FC8))$; $F8 = 10^{(\log10(FC8) / (1 + (\log10(KR8) / NC8)2))}$; KMT08 = (K80 × K8I) × F8 / (K80 + K8I) ; $K90 = 1.4 \times 10^{-31} \times M \times (T / 300)^{-3.1}$;

Reference

1. Wavefunction, I., Spartan’18 version 1.4.5 (Irvine, CA).
2. Chai, J.-D.; Head-Gordon, M., Long-range corrected hybrid density functionals with damped atom–atom dispersion corrections. *Geosci. Model Dev.* **2008,** *10*, (44), 6615-6620.
3. Kendall, R. A.; Jr., T. H. D.; Harrison, R. J., Electron affinities of the first-row atoms revisited. Systematic basis sets and wave functions. *J. Chem. Phys.* **1992,** *96*, (9), 6796-6806.
4. Peterson, K. A.; Figgen, D.; Goll, E.; Stoll, H.; Dolg, M., Systematically convergent basis sets with relativistic pseudopotentials. II. Small-core pseudopotentials and correlation consistent basis sets for the post-d group 16–18 elements. *J. Chem. Phys.* **2003,** *119*, (21), 11113-11123.
5. Feller, D., The role of databases in support of computational chemistry calculations. *J. Comput. Chem.* **1996,** *17*, (13), 1571-1586.
6. Frisch, M. J.; Trucks, G. W.; Schlegel, H. B.; Scuseria, G. E.; Robb, M. A.; Cheeseman, J. R.; Scalmani, G.; Barone, V.; Petersson, G. A.; Nakatsuji, H.; Li, X.; Caricato, M.; Marenich, A. V.; Bloino, J.; Janesko, B. G.; Gomperts, R.; Mennucci, B.; Hratchian, H. P.; Ortiz, J. V.; Izmaylov, A. F.; Sonnenberg, J. L.; Williams; Ding, F.; Lipparini, F.; Egidi, F.; Goings, J.; Peng, B.; Petrone, A.; Henderson, T.; Ranasinghe, D.; Zakrzewski, V. G.; Gao, J.; Rega, N.; Zheng, G.; Liang, W.; Hada, M.; Ehara, M.; Toyota, K.; Fukuda, R.; Hasegawa, J.; Ishida, M.; Nakajima, T.; Honda, Y.; Kitao, O.; Nakai, H.; Vreven, T.; Throssell, K.; Montgomery Jr., J. A.; Peralta, J. E.; Ogliaro, F.; Bearpark, M. J.; Heyd, J. J.; Brothers, E. N.; Kudin, K. N.; Staroverov, V. N.; Keith, T. A.; Kobayashi, R.; Normand, J.; Raghavachari, K.; Rendell, A. P.; Burant, J. C.; Iyengar, S. S.; Tomasi, J.; Cossi, M.; Millam, J. M.; Klene, M.; Adamo, C.; Cammi, R.; Ochterski, J. W.; Martin, R. L.; Morokuma, K.; Farkas, O.; Foresman, J. B.; Fox, D. J. *Gaussian 16 Rev. C.01*, Wallingford, CT, 2016.
7. Neese, F., The ORCA program system. *WIREs Comput.l Mol. Sci.* **2012,** *2*, (1), 73-78.
8. Riplinger, C.; Neese, F., An efficient and near linear scaling pair natural orbital based local coupled cluster method. *J. Chem. Phys.* **2013,** *138*, (3), 034106.
9. Breitenlechner, M.; Fischer, L.; Hainer, M.; Heinritzi, M.; Curtius, J.; Hansel, A., PTR3: An Instrument for Studying the Lifecycle of Reactive Organic Carbon in the Atmosphere. *Anal. Chem.* **2017,** *89*, (11), 5824-5831.
10. Canaval, E.; Hyttinen, N.; Schmidbauer, B.; Fischer, L.; Hansel, A., NH4+ Association and Proton Transfer Reactions With a Series of Organic Molecules. *Front. Chem.* **2019,** *7*, (191).
11. Hansel, A.; Jordan, A.; Holzinger, R.; Prazeller, P.; Vogel, W.; Lindinger, W., Proton transfer reaction mass spectrometry: on-line trace gas analysis at the ppb level. *Int. J. Mass Spectrom. Ion Processes* **1995,** *149-150*, 609-619.
12. Jokinen, T.; Sipilä, M.; Junninen, H.; Ehn, M.; Lönn, G.; Hakala, J.; Petäjä, T.; Mauldin Iii, R. L.; Kulmala, M.; Worsnop, D. R., Atmospheric sulphuric acid and neutral cluster measurements using CI-APi-TOF. *Atmos. Chem. Phys.* **2012,** *12*, (9), 4117-4125.
13. Kürten, A.; Rondo, L.; Ehrhart, S.; Curtius, J., Performance of a corona ion source for measurement of sulfuric acid by chemical ionization mass spectrometry. *Atmos. Meas. Tech.* **2011,** *4*, (3), 437-443.

14. Kürten, A.; Rondo, L.; Ehrhart, S.; Curtius, J., Calibration of a Chemical Ionization Mass Spectrometer for the Measurement of Gaseous Sulfuric Acid. *J Phys. Chem. A* **2012,** *116*, (24), 6375-6386.
15. Iyer, S.; Lopez-Hilfiker, F.; Lee, B. H.; Thornton, J. A.; Kurtén, T., Modeling the Detection of Organic and Inorganic Compounds Using Iodide-Based Chemical Ionization. *J. Phys. Chem. A* **2016,** *120*, (4), 576-587.
16. Wang, M.; He, X. C.; Finkenzeller, H.; Iyer, S.; Chen, D.; Shen, J.; Simon, M.; Hofbauer, V.; Kirkby, J.; Curtius, J.; Maier, N.; Kurtén, T.; Worsnop, D. R.; Kulmala, M.; Rissanen, M.; Volkamer, R.; Tham, Y. J.; Donahue, N. M.; Sipilä, M., Measurement of iodine species and sulfuric acid using bromide chemical ionization mass spectrometers. *Atmos. Meas. Tech.* **2021,** *14*, (6), 4187-4202.
17. Hansel, A.; Scholz, W.; Mentler, B.; Fischer, L.; Berndt, T., Detection of RO_2 radicals and other products from cyclohexene ozonolysis with NH_4^+ and acetate chemical ionization mass spectrometry. *Atmos. Environ.* **2018,** *186*, 248-255.
18. Zaytsev, A.; Breitenlechner, M.; Koss, A. R.; Lim, C. Y.; Rowe, J. C.; Kroll, J. H.; Keutsch, F. N., Using collision-induced dissociation to constrain sensitivity of ammonia chemical ionization mass spectrometry (NH_4^+-CIMS) to oxygenated volatile organic compounds. *Atmos. Meas. Tech.* **2019,** *12*, (3), 1861-1870.
19. Lopez-Hilfiker, F. D.; Mohr, C.; Ehn, M.; Rubach, F.; Kleist, E.; Wildt, J.; Mentel, T. F.; Lutz, A.; Hallquist, M.; Worsnop, D.; Thornton, J. A., A novel method for online analysis of gas and particle composition: description and evaluation of a Filter Inlet for Gases and AEROsols (FIGAERO). *Atmos. Meas. Tech.* **2014,** *7*, (4), 983-1001.
20. Rissanen, M. P.; Mikkilä, J.; Iyer, S.; Hakala, J., Multi-scheme chemical ionization inlet (MION) for fast switching of reagent ion chemistry in atmospheric pressure chemical ionization mass spectrometry (CIMS) applications. *Atmos. Meas. Tech.* **2019,** *12*, (12), 6635-6646.
21. Veres, P. R.; Neuman, J. A.; Bertram, T. H.; Assaf, E.; Wolfe, G. M.; Williamson, C. J.; Weinzierl, B.; Tilmes, S.; Thompson, C. R.; Thames, A. B.; Schroder, J. C.; Saiz-Lopez, A.; Rollins, A. W.; Roberts, J. M.; Price, D.; Peischl, J.; Nault, B. A.; Møller, K. H.; Miller, D. O.; Meinardi, S.; Li, Q.; Lamarque, J.-F.; Kupc, A.; Kjaergaard, H. G.; Kinnison, D.; Jimenez, J. L.; Jernigan, C. M.; Hornbrook, R. S.; Hills, A.; Dollner, M.; Day, D. A.; Cuevas, C. A.; Campuzano-Jost, P.; Burkholder, J.; Bui, T. P.; Brune, W. H.; Brown, S. S.; Brock, C. A.; Bourgeois, I.; Blake, D. R.; Apel, E. C.; Ryerson, T. B., Global airborne sampling reveals a previously unobserved dimethyl sulfide oxidation mechanism in the marine atmosphere. *Proc. Natl. Acad. Sci. U.S.A.* **2020,** *117*, (9), 4505.
22. Ye, Q.; Goss, M. B.; Isaacman-VanWertz, G.; Zaytsev, A.; Massoli, P.; Lim, C.; Croteau, P.; Canagaratna, M.; Knopf, D. A.; Keutsch, F. N.; Heald, C. L.; Kroll, J. H., Organic Sulfur Products and Peroxy Radical Isomerization in the OH Oxidation of Dimethyl Sulfide. *ACS Earth and Space Chem.* **2021,** *5*, (8), 2013-2020.
23. Saunders, S. M.; Jenkin, M. E.; Derwent, R. G.; Pilling, M. J., Protocol for the development of the Master Chemical Mechanism, MCM v3 (Part A): tropospheric degradation of non-aromatic volatile organic compounds. *Atmos. Chem. Phys.* **2003,** *3*, (1), 161-180.
24. Hoffmann, E. H.; Tilgner, A.; Schrödner, R.; Bräuer, P.; Wolke, R.; Herrmann, H., An advanced modeling study on the impacts and atmospheric implications of

multiphase dimethyl sulfide chemistry. *Proc. Natl. Acad. Sci. U.S.A.* **2016,** *113,* (42), 11776-11781.

25. Wu, R.; Wang, S.; Wang, L., New Mechanism for the Atmospheric Oxidation of Dimethyl Sulfide. The Importance of Intramolecular Hydrogen Shift in a CH_3SCH_2OO Radical. *J. Phys. Chem. A* **2015,** *119,* (1), 112-117.

26. Berndt, T.; Scholz, W.; Mentler, B.; Fischer, L.; Hoffmann, E. H.; Tilgner, A.; Hyttinen, N.; Prisle, N. L.; Hansel, A.; Herrmann, H., Fast Peroxy Radical Isomerization and OH Recycling in the Reaction of OH Radicals with Dimethyl Sulfide. *J. Phys. Chem. Lett.* **2019,** *10,* (21), 6478-6483.

27. Berndt, T.; Chen, J.; Møller, K. H.; Hyttinen, N.; Prisle, N. L.; Tilgner, A.; Hoffmann, E. H.; Herrmann, H.; Kjaergaard, H. G., SO_2 formation and peroxy radical isomerization in the atmospheric reaction of OH radicals with dimethyl disulfide. *Chem. Commun* **2020,** *56,* (88), 13634-13637.

28. Orlando, J. J.; Tyndall, G. S., Laboratory studies of organic peroxy radical chemistry: an overview with emphasis on recent issues of atmospheric significance. *Chem. Soc. Rev.* **2012,** *41,* (19), 6294-6317.

29. Jenkin, M. E.; Saunders, S. M.; Pilling, M. J., The tropospheric degradation of volatile organic compounds: a protocol for mechanism development. *Atmos. Environ.* **1997,** *31,* (1), 81-104.

30. Jernigan, C. M.; Fite, C. H.; Vereecken, L.; Berkelhammer, M. B.; Rollins, A. W.; Rickly, P. S.; Novelli, A.; Taraborrelli, D.; Holmes, C. D.; Bertram, T. H., Efficient Production of Carbonyl Sulfide in the Low-NOx Oxidation of Dimethyl Sulfide. *Geophys. Res. Lett.* **2022,** *49,* (3), e2021GL096838.

31. Yin, F.; Grosjean, D.; Seinfeld, J. H. J. J. o. A. C., Photooxidation of dimethyl sulfide and dimethyl disulfide. I: Mechanism development. *J. Atmos. Chem.* **1990,** *11,* (4), 309-364.

32. Arsene, C.; Barnes, I.; Becker, K. H.; Schneider, W. F.; Wallington, T. T.; Mihalopoulos, N.; Patroescu-Klotz, I. V., Formation of Methane Sulfinic Acid in the Gas-Phase OH-Radical Initiated Oxidation of Dimethyl Sulfoxide. *Environ. Sci. Tech.* **2002,** *36,* (23), 5155-5163.

33. Kukui, A.; Borissenko, D.; Laverdet, G.; Le Bras, G., Gas-Phase Reactions of OH Radicals with Dimethyl Sulfoxide and Methane Sulfinic Acid Using Turbulent Flow Reactor and Chemical Ionization Mass Spectrometry. *J. Phys. Chem. A* **2003,** *107,* (30), 5732-5742.

34. Tian, Y.; Tian, Z.-M.; Wei, W.-M.; He, T.-J.; Chen, D.-M.; Liu, F.-C. J. C. p., Ab initio study of the reaction of OH radical with methyl sulfinic acid (MSIA). *Chem. Phys.* **2007,** *335,* (2-3), 133-140.

35. Donahue, N. M., Reaction Barriers: Origin and Evolution. *Chem. Rev.* **2003,** *103,* (12), 4593-4604.

36. Lv, G.; Zhang, C.; Sun, X., Understanding the oxidation mechanism of methanesulfinic acid by ozone in the atmosphere. *Sci Rep* **2019,** *9,* (1), 322.

37. Lucas, D. D.; Prinn, R. G., Mechanistic studies of dimethylsulfide oxidation products using an observationally constrained model. *J. Geophys. Res. Atmos.* **2002,** *107,* (D14), ACH 12.

38. Chen, Q.; Sherwen, T.; Evans, M.; Alexander, B., DMS oxidation and sulfur aerosol formation in the marine troposphere: a focus on reactive halogen and multiphase chemistry. *Atmos. Chem. Phys.* **2018,** *18*, (18), 13617-13637.
39. Berresheim, H.; Adam, M.; Monahan, C.; O'Dowd, C.; Plane, J. M. C.; Bohn, B.; Rohrer, F., Missing SO_2 oxidant in the coastal atmosphere? – observations from high-resolution measurements of OH and atmospheric sulfur compounds. *Atmos. Chem. Phys.* **2014,** *14*, (22), 12209-12223.40.
40 He, X.-C.; Iyer, S.; Sipilä, M.; Ylisirniö, A.; Peltola, M.; Kontkanen, J.; Baalbaki, R.; Simon, M.; Kürten, A.; Tham, Y. J.; et al. Determination of the collision rate coefficient between charged iodic acid clusters and iodic acid using the appearance time method. Aerosol Sci. and Tech. **2021**, 55 (2), 231-242.
41. Berndt, T.; Richters, S., Products of the reaction of OH radicals with dimethyl sulphide in the absence of NO_x: Experiment and simulation. *Atmos. Environ.* **2012,** *47*, 316-322.
42. Wollesen de Jonge, R.; Elm, J.; Rosati, B.; Christiansen, S.; Hyttinen, N.; Lüdemann, D.; Bilde, M.; Roldin, P., Secondary aerosol formation from dimethyl sulfide – improved mechanistic understanding based on smog chamber experiments and modelling. *Atmos. Chem. Phys.* **2021,** *21*, (13), 9955-9976.
43. Gershenzon, M.; Davidovits, P.; Jayne, J. T.; Kolb, C. E.; Worsnop, D. R. Simultaneous Uptake of DMS and Ozone on Water. *J. Phys. Chem. A* **2001**, *105* (29), 7031-7036.
44. Bernhammer, A. K.; Breitenlechner, M.; Keutsch, F. N.; Hansel, A. Technical note: Conversion of isoprene hydroxy hydroperoxides (ISOPOOHs) on metal environmental simulation chamber walls. *Atmos. Chem. Phys.* **2017**, *17* (6), 4053-4062.
45. Beck, L. J.; Sarnela, N.; Junninen, H.; Hoppe, C. J. M.; Garmash, O.; Bianchi, F.; Riva, M.; Rose, C.; Peräkylä, O.; Wimmer, D.; Kausiala, O.; Jokinen, T.; Ahonen, L.; Mikkilä, J.; Hakala, J.; He, X. C.; Kontkanen, J.; Wolf, K. K. E.; Cappelletti, D.; Mazzola, M.; Traversi, R.; Petroselli, C.; Viola, A. P.; Vitale, V.; Lange, R.; Massling, A.; Nøjgaard, J. K.; Krejci, R.; Karlsson, L.; Zieger, P.; Jang, S.; Lee, K.; Vakkari, V.; Lampilahti, J.; Thakur, R. C.; Leino, K.; Kangasluoma, J.; Duplissy, E. M.; Siivola, E.; Marbouti, M.; Tham, Y. J.; Saiz-Lopez, A.; Petäjä, T.; Ehn, M.; Worsnop, D. R.; Skov, H.; Kulmala, M.; Kerminen, V. M.; Sipilä, M., Differing mechanisms of new particle formation at two Arctic sites. *Geophys. Res. Lett.* **2021,** *48*, (4), e2020GL091334.
46. Dal Maso, M.; Kulmala, M.; Lehtinen, K. E. J.; Mäkelä, J. M.; Aalto, P.; O'Dowd, C. D., Condensation and coagulation sinks and formation of nucleation mode particles in coastal and boreal forest boundary layers. *J. Geophys. Res. Atmos.* **2002,** *107,* (D19), PAR 2-1-PAR 2-10.
47. Ranjithkumar, A.; Gordon, H.; Williamson, C.; Rollins, A.; Pringle, K.; Kupc, A.; Abraham, N. L.; Brock, C.; Carslaw, K., Constraints on global aerosol number concentration, SO_2 and condensation sink in UKESM1 using ATom measurements. *Atmos. Chem. Phys.* **2021,** *21*, (6), 4979-5014.
48. Pardalos, P. M.; Mavridou, T. D., Simulated annealing. In *Encyclopedia of Optimization*, Springer US: Boston, MA, 2009; pp 3591-3593.
49. Edtbauer, A.; Stönner, C.; Pfannerstill, E. Y.; Berasategui, M.; Walter, D.; Crowley, J. N.; Lelieveld, J.; Williams, J., A new marine biogenic emission: methane

sulfonamide (MSAM), dimethyl sulfide (DMS), and dimethyl sulfone ($DMSO_2$) measured in air over the Arabian Sea. *Atmos. Chem. Phys.* **2020,** *20*, (10), 6081-6094.

50. Yan, J.; Jung, J.; Zhang, M.; Xu, S.; Lin, Q.; Zhao, S.; Chen, L., Significant Underestimation of Gaseous Methanesulfonic Acid (MSA) over Southern Ocean. *Environ. Sci. Tech.* **2019,** *53*, (22), 13064-13070.

51. Berresheim, H.; Elste, T.; Tremmel, H. G.; Allen, A. G.; Hansson, H.-C.; Rosman, K.; Dal Maso, M.; Mäkelä, J. M.; Kulmala, M.; O'Dowd, C. D., Gas-aerosol relationships of H_2SO_4, MSA, and OH: Observations in the coastal marine boundary layer at Mace Head, Ireland. *J. Geophys. Res. Atmos.* **2002,** *107*, (D19), PAR 5-1-PAR 5-12.

52. Jokinen, T.; Sipilä, M.; Kontkanen, J.; Vakkari, V.; Tisler, P.; Duplissy, E.-M.; Junninen, H.; Kangasluoma, J.; Manninen, H. E.; Petäjä, T.; Kulmala, M.; Worsnop, D. R.; Kirkby, J.; Virkkula, A.; Kerminen, V.-M., Ion-induced sulfuric acid-ammonia nucleation drives particle formation in coastal Antarctica. *Sci Adv* **2018,** *4*, (11), eaat9744.

53. Wang, M.; Kong, W.; Marten, R.; He, X.-C.; Chen, D.; Pfeifer, J.; Heitto, A.; Kontkanen, J.; Dada, L.; Kürten, A.; Yli-Juuti, T.; Manninen, H. E.; Amanatidis, S.; Amorim, A.; Baalbaki, R.; Baccarini, A.; Bell, D. M.; Bertozzi, B.; Bräkling, S.; Brilke, S.; Murillo, L. C.; Chiu, R.; Chu, B.; De Menezes, L.-P.; Duplissy, J.; Finkenzeller, H.; Carracedo, L. G.; Granzin, M.; Guida, R.; Hansel, A.; Hofbauer, V.; Krechmer, J.; Lehtipalo, K.; Lamkaddam, H.; Lampimäki, M.; Lee, C. P.; Makhmutov, V.; Marie, G.; Mathot, S.; Mauldin, R. L.; Mentler, B.; Müller, T.; Onnela, A.; Partoll, E.; Petäjä, T.; Philippov, M.; Pospisilova, V.; Ranjithkumar, A.; Rissanen, M.; Rörup, B.; Scholz, W.; Shen, J.; Simon, M.; Sipilä, M.; Steiner, G.; Stolzenburg, D.; Tham, Y. J.; Tomé, A.; Wagner, A. C.; Wang, D. S.; Wang, Y.; Weber, S. K.; Winkler, P. M.; Wlasits, P. J.; Wu, Y.; Xiao, M.; Ye, Q.; Zauner-Wieczorek, M.; Zhou, X.; Volkamer, R.; Riipinen, I.; Dommen, J.; Curtius, J.; Baltensperger, U.; Kulmala, M.; Worsnop, D. R.; Kirkby, J.; Seinfeld, J. H.; El-Haddad, I.; Flagan, R. C.; Donahue, N. M., Rapid growth of new atmospheric particles by nitric acid and ammonia condensation. *Nature* **2020,** *581*, (7807), 184-189.

54. Lee, B. H.; Lopez-Hilfiker, F. D.; Mohr, C.; Kurtén, T.; Worsnop, D. R.; Thornton, J. A., An Iodide-Adduct High-Resolution Time-of-Flight Chemical-Ionization Mass Spectrometer: Application to Atmospheric Inorganic and Organic Compounds. *Environ. Sci. Tech.* **2014,** *48*, (11), 6309-6317.

55. Sander, S. P.; Abbatt, J. P. D.; Barker, J. R.; Burkholder, J. B.; Friedl, R. R.; Golden, D. M.; Huie, R. E.; Kolb, C. E.; Kurylo, M. J.; Moortgat, G. K.; Orkin, V. L.; Wine, P. H., Chemical kinetics and photochemical data for use in atmospheric studies: Evaluation number 17; *Jet Propulsion Laboratory, Pasadena, CA*, **2011**.
